# Progress in polymers and polymer composites used as efficient materials for EMI shielding

**DOI:** 10.1039/d0na00760a

**Published:** 2020-11-10

**Authors:** Ján Kruželák, Andrea Kvasničáková, Klaudia Hložeková, Ivan Hudec

**Affiliations:** Department of Plastics, Rubber and Fibres, Faculty of Chemical and Food Technology, Slovak University of Technology Radlinského 9 812 37 Bratislava Slovakia jan.kruzelak@stuba.sk +421 02 5932589

## Abstract

The explosive progress of electronic devices and communication systems results in the production of undesirable electromagnetic pollution, known as electromagnetic interference. The accumulation of electromagnetic radiation in space results in the malfunction of commercial and military electronic appliances, and it may have a negative impact on human health. Thus, the shielding of undesirable electromagnetic interference has become a serious concern of the modern society, and has been a very perspective field of research and development. This paper provides detailed insight into current trends in the advancement of various polymer-based materials with the effects of electromagnetic interference shielding. First, the theoretical aspects of shielding are outlined. Then, the comprehensive description of the structure, morphology and functionalization of the intrinsic conductive polymers, polymers filled with the different types of inorganic and organic fillers, as well as multifunctional polymer architectures are provided with respect to their conductive, dielectric, magnetic and shielding characteristics.

## Abstract 2

The work provides a detailed overview of the newest research of polymers and polymer composites being used as efficient EMI shields.

## Graphical abstract

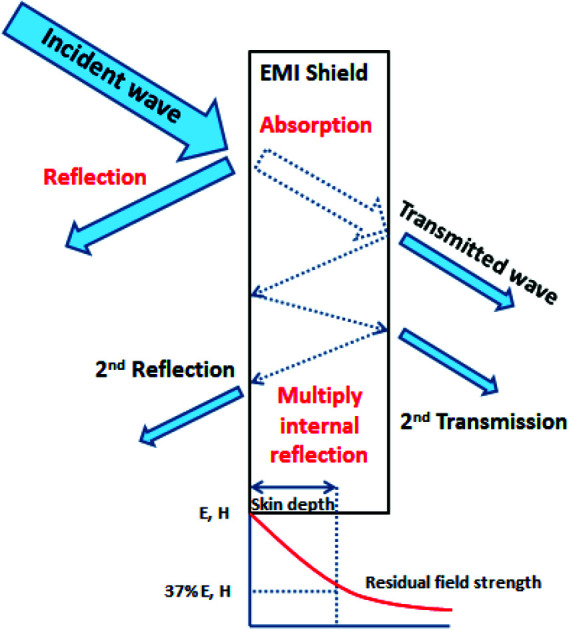

## 1 Introduction

Electromagnetic radiation is a form of energy with wave character that is emitted or absorbed by charged particles. Electromagnetic radiation has existed in the ambient environment within living memory. Although its source is mainly the sun, all animals, plants and human beings also produce weak electromagnetic fields. However, in the modern age of electricity, various electric appliances, mobile phones, satellites, radio and TV towers, the so-called artificial electromagnetic radiation produced by those mechanisms has started to appear and accumulate in the surroundings. This radiation is often termed as electromagnetic smog, or electromagnetic interference (EMI).

Electromagnetic radiation waves from these devices can interfere with other electronic appliances, which leads to the lowering of their efficiency, and in some cases even to their malfunction.^1,2^ These factors can also affect the functions of the human body, mainly when organisms are subjected to their exposure for a longer period of time. The most common symptoms are headaches, irritability, insomnia, fatigue or failure of attention, which may be the reason for more serious illnesses.^3–5^ Therefore, the need electromagnetic radiation shielding has become increasingly pronounced over the last decades, and is a very prospective sphere of research and investigations.

The most common materials for EMI shielding are metals due to their high conductivity. However, their high weight density, low flexibility, propensity to corrosion, heavy processing or manipulation limit the utilization of metals and metal composites in modern devices. To overcome the limitations of metal-based EMI shielding materials, a lot of effort has been paid to the development of polymer materials and composites due to their easy processability, flexibility, low specific weight, chemical and corrosion resistance, or tunable structural and mechanical properties. In addition, in contrast to the reflection dominated EMI shielding of metals, the polymer-based materials exhibit the ability to shield electromagnetic waves primarily through absorption, which has been increasingly preferred in many applications, such as in military or stealth technology. Intrinsically conductive polymers with delocalized π-conjugated electronic structure demonstrate unusual electronic properties, such as low ionization potential, high electron affinity and conductivity, which can be significantly enhanced by chemical or filler doping.^6^ The generally used polymers are typical electrical insulators. Therefore, they are not able to provide shielding effects. However, the introduction of suitable fillers results in the preparation of polymer composites with unique electromagnetic properties. The application of a suitable filler or filler combinations can not only provide the opportunity to tune the physical–mechanical properties of the polymer composites, but also provide the possibility to adjust the permittivity, permeability, thermal and electrical conductivity, or thickness to obtain improved EMI performance. Thus, the polymer composites have become versatile materials with tunable mechanical, dynamic, optic and electromagnetic properties, which helps to expand their application field. The polymers and polymer composites demonstrate great promise as light weight, thermally stable, mechanically strong, ultra-efficient EMI shielding materials in advanced application fields, such as in electronics, radars, flexible portable and wearable electronic devices, aircraft, defense, aerospace applications, military applications or stealth technology.

## 2 Mechanisms of EMI shielding

Three mechanisms contribute to the overall effectiveness of electromagnetic radiation or electromagnetic interference (EMI) shielding, namely absorption, reflection and multiple reflection.^7–9^

The primary mechanism of EMI shielding is usually reflection. The principle is based on the simple reflection of EM radiation from the surface of the shielding material. The material that is able to reflect EMI must be a carrier of free electric charges (electrons or holes), which interact with the electromagnetic field in the radiation.^10^ The shielding material must therefore be conductive, although high conductivity is specifically not required (a volume resistivity of around 1 Ω cm is typically sufficient). There is also no requirement to reach the percolation threshold or the connectivity of conductive fillers incorporated into dielectric matrices in order to provide good reflection efficiency. However, increasing the amount of conductive filler paths in composites leads to the significant increase in the shielding effectiveness. Typically, the materials that have good efficiency to shield EMI by reflection are various metals (copper, nickel, aluminum, silver, gold, *etc.*); this is due to the high amount of free electrons that are able to conduct electric current.^11–13^ Composites containing conductive fillers as metal powders or carbon fibers, and materials that have been surface-treated with conductive layers and coatings are the next examples of materials that provide good shielding efficiency by reflection. In general, it can be stated that over 103 Hz, the efficiency of reflection is improved with increasing shield conductivity, and deteriorates with increasing shield permeability and frequency of electromagnetic radiation.

Absorption is a secondary mechanism of EMI shielding, and depends on the thickness of the shielding material. The efficiency of shielding by absorption increases with the presence of electric and/or magnetic dipoles in the shield that are able to interact with the EM radiation.^7,14^ The sources of the electric dipoles are mainly materials with high values of dielectric constant (permittivity), for instance, BaTiO_3_, Fe_2_O_3_, and ZrO_2_. Magnetic dipoles have materials with high permeability. Fe_3_O_4_, ferrites, super permalloy and mumetal (alloy of iron and nickel) belong to the excellent absorption materials due to their high values of permeability.^15–18^ The efficiency of absorption increases with increasing frequency of EM radiation, with increasing thickness and permeability of the shielding material. In the absence of magnetic properties, EMI shielding is dependent exclusively on the dielectric properties and *vice versa*.^19^

In addition to the reflection and absorption, the third mechanism of EMI shielding is multiple reflection, which refers to the reflection of EM radiation from various surfaces, phase interfaces and in-homogeneities in the shield.^20^ Materials with good multiple reflection ability exhibit high specific surface area (foam or porous materials) or a large phase interface (composites containing fillers with high specific surface areas).^14,21^ The amplitude of the incident radiation is attenuated by multiple internal reflections within the shielding material.

Electromagnetic radiation at high frequencies can only penetrate to the near surface regions of the electric conductors. This is known as the skin effect.^22,23^ The strength of the electromagnetic incident wave declines exponentially when passing through the conductor. The depth at which the electric field drops to 1/*e* (*e* is Euler's number and 1/*e* = 0.37) of the incident value is called the skin depth (*δ*):1
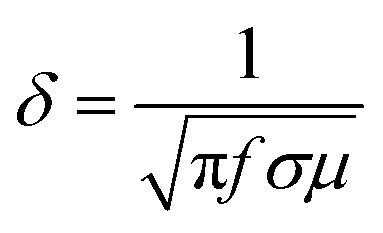
where: *f* – frequency of electromagnetic radiation, *σ* – shield electric conductivity (Ω^−1^ m^−1^) a *μ* – shield magnetic permeability, *μ* = *μ*_o_*μ*_r_, where *μ*_r_ = shield relative magnetic permeability, and *μ*_o_ – permeability of air or free space (*μ*_o_ = 4π × 10^−7^ H m^−1^). It becomes evident that the skin depth decreases with the increase in the EM wave frequency, magnetic permeability and conductivity of the shield. Owing to the skin effect, composites containing conductive fillers with a small unit dimension are more effective for shielding when compared to the fillers with a large unit size. It has been reported that the unit size of the filler should be less or comparable to the skin depth.^7^ Thus, the filler with a unit size of 1 μm or less is typically preferred. However, the dispersion and distribution of small fillers in polymer matrices are often very difficult. They also tend to agglomerate, leading to dispersive non-homogeneity, which may have negative effects not only on the processability and physical–mechanical properties of composite materials, but also on their shielding performance.

Following the transmission line theory and plane-wave theory, the shielding mechanism of the EMI shield can be inferred with respect to the skin depth and thickness as:

(a) When the thickness of the EMI shield is much lower than the skin depth (*t* ≪ *δ*), the attenuation occurs solely by reflection. This condition takes place either at low frequencies, or in the case of thin material with good electrical conductivity. Under these conditions, the total shielding phenomenon is independent of frequency.

(b) When the thickness of the EMI shield is much larger than the skin depth (*t* ≫ *δ*), the attenuation occurs by reflection, absorption and multiple internal sub-phenomenon for good conductors, whose *σ*/*ωε* ≫ 1. In this case, the total shielding phenomenon is dependent on frequency. This condition is fulfilled either at high frequencies, or in the case of an electrically conductive thick sample.^24^

**Fig. 1:**
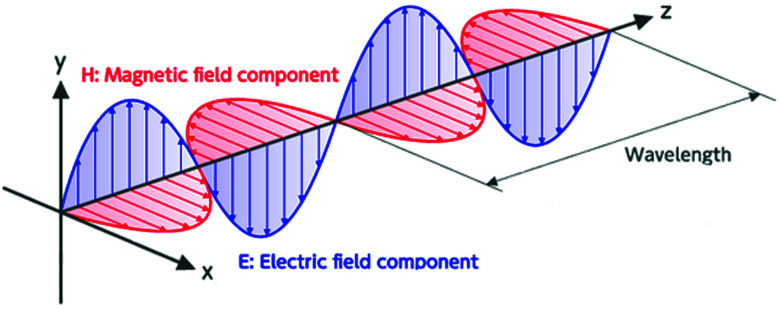
Schematic illustration of an electromagnetic transverse oscillating wave.

**Fig. 2:**
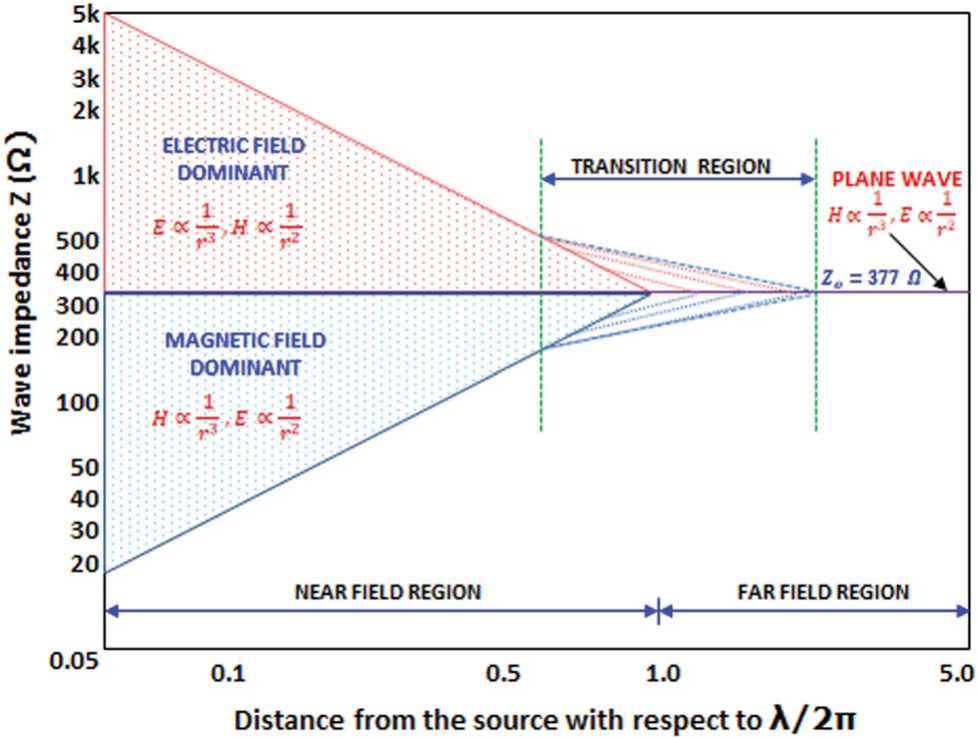
Dependence of the wave impedance on the distance from the source normalized to *λ*/2π (modified from ref. 24 with permission from Intech, copyright 2012).

The electromagnetic radiation can be imagined as a self-propagating transverse oscillating wave. From Fig. 1, it becomes evident that the electromagnetic wave consists of two essential elements, a magnetic field (*H*) and an electric field (*E*). These two fields are perpendicular to each other, and the direction of the wave propagation is at a right angle to the plane containing the two components. The relative magnitude depends on the waveform and its source. The ratio of the amplitudes of the electric and magnetic fields is called wave impedance *Z* (|*E*|/|*H*|). The wave impedance decreases with increasing distance from the source. The magnetic field and the electric field both decrease in amplitude by 20 dB if the distance is increased by ten times. In a certain distance from the source, the transmitted wave is changed into a plane wave with a constant value of impedance *Z*_0_ = 377 Ω (impedance of free space).^25^ The region with a constant value of wave impedance is the far-field shielding region (radiation field or Fraunhofer zone) (Fig. 2).^26^ In the far-field shielding region, the distance between the radiation source and the shield is higher than *λ*/2π (where *λ* is the free-space wavelength of the radiation). The electromagnetic plane wave theory is generally applied for the EMI shielding in this region. When the distance between the source and the shield is lower than *λ*/2π, it is the near-field shielding. The theory based on the contribution of the electric and magnetic dipoles is then used for EMI shielding.^27^ In the near-field region (reactive field or Fresnel zone), (|*E*|/|*H*|) is not constant and the shielding effectiveness must be observed separately for the magnetic field and electric field. The ratio between the fields depends on the distance from the radiation source. For high current low voltage sources, the near-field is mainly magnetic. However, for low current high voltage sources, the electric field dominates.^28^ The magnetic field controls the near-field when the source has low impedance. On the other hand, the electric field takes over when the source has high impedance. Between the near-field and far-field regions, there exists a transition region, in which the distance between the radiation source and the shield is roughly equal to *λ*/2π (Fig. 2).^29^ Electromagnetic waves are categorized based on the frequency as radio waves, microwaves, visible light, ultraviolet waves, X-rays, and gamma rays. Electronic devices operating in diverse frequency ranges constitute several electromagnetic bands. Table 1 presents the applications of the EM wave frequency bands, especially in the RF and MW regions.^30^

**Table 1:** Applications of EM bands in the RF (radio frequency) and MW (medium wave) frequency region, VLF – very low frequency, LF – low frequency, MF – medium frequency, HF – high frequency, VHF – very high frequency, UHF – ultra high frequency

Type of EM wave	Band frequency (Hz)	Band name	Applications
RF wave	3–KHz	VLF	Navigation, time signals, submarine communication, geophysics
	30–300 KHz	LF	Navigation, AM long-wave broadcast
	300 KHz to 3 MHz	MF	AM medium-wave broadcast, amateur radio
	3–30 MHz	HF	AM short-wave broadcast, radiofrequency identifications, marine and mobile radio telephony
	30–300 MHz	VHF	FM radio broadcast, television broadcast
MW	300 MHz to 1 GHz	UHF	Television, microwave oven, mobile phones
	1–2 GHz	L band	Mobile phones, wireless LAN, radars, GPS
	2–4 GHz	S band	Bluetooth, cordless phones, mobile phones, television
	4–8.2 GHz	C band, J band	Satellite communication, cordless telephone, wifi
	8.2–12.4 GHz	X band	Satellite communication, weather monitoring, defence tracking, air traffic control
	12.4–18 GHz	Ku band	Satellite communication
	18–27 GHz	K band	Satellite communication
	27–40 GHz	Ka band	Satellite communication
	40–75 GHz	V band	Military and research
	75–110 GHz	W band	Military and research
	110–300 GHz	mm band	Research

The shielding effectiveness (SE) is the ratio of the impinging energy to the residual energy. The SE of the material can be defined as the function of the logarithm ratio of the incident radiation power to the transmitted power, and is generally expressed in decibel (dB):2
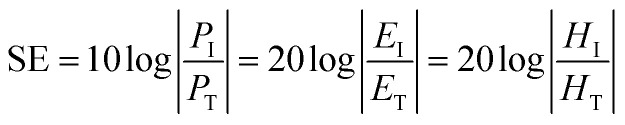
where *P*, *E*, and *H* represent the plane-wave field intensity, electric and magnetic field intensities, respectively. Subscript I designates the incident wave, while T represents the transmitted wave.^27,31,32^ In general, EMI occurs in the frequency range from 10^4^ to 10^12^ Hz of the electromagnetic spectrum. With increasing EMI SE level, there is less energy transmitted through the shield. A shielding effectiveness in the range of 10–30 dB is considered to be the minimum effective range of shielding, and is suitable for most applications. It has been shown that a shielding material reaching 30 dB of EMI SE is able to block 99.9% of the incident radiation, which is the required value for the majority of commercial and industrial applications.^33,34^ EMI shields with SE ≥ 20 dB can attenuate 99% of the impinging EM energy.^35,36^

**Fig. 3:**
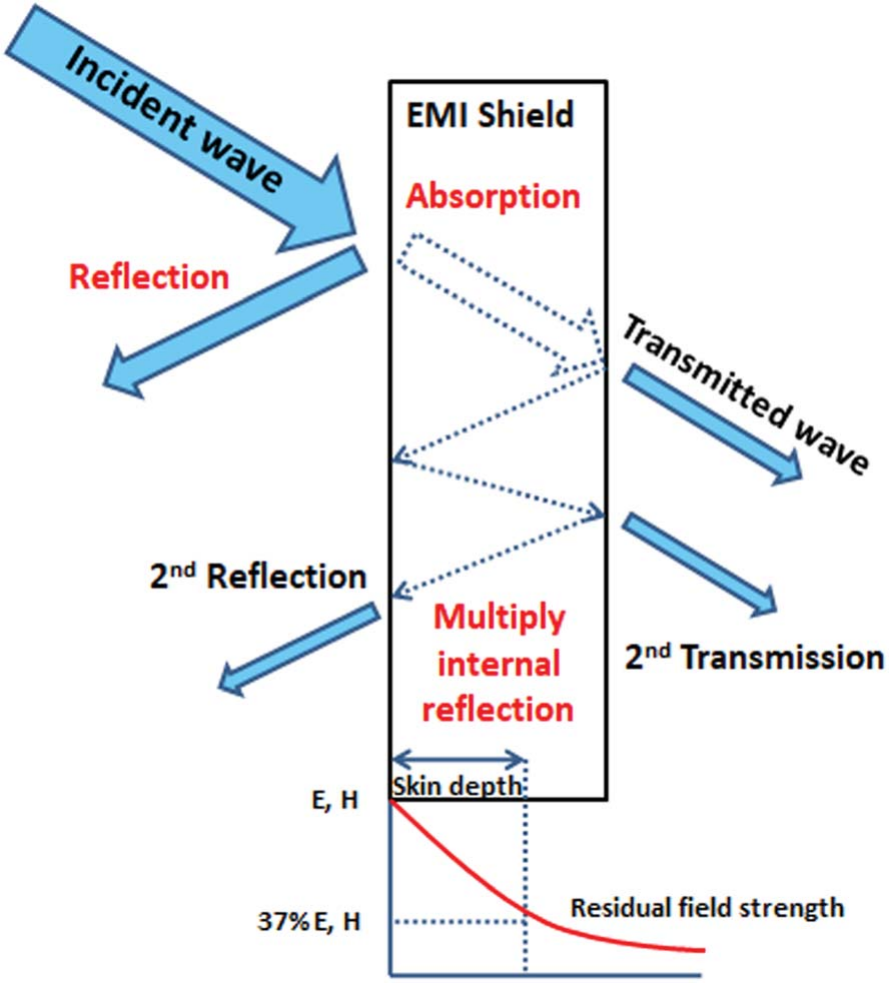
Schematic representation of the EMI shielding mechanisms for the thin plate shield.

**Fig. 4:**
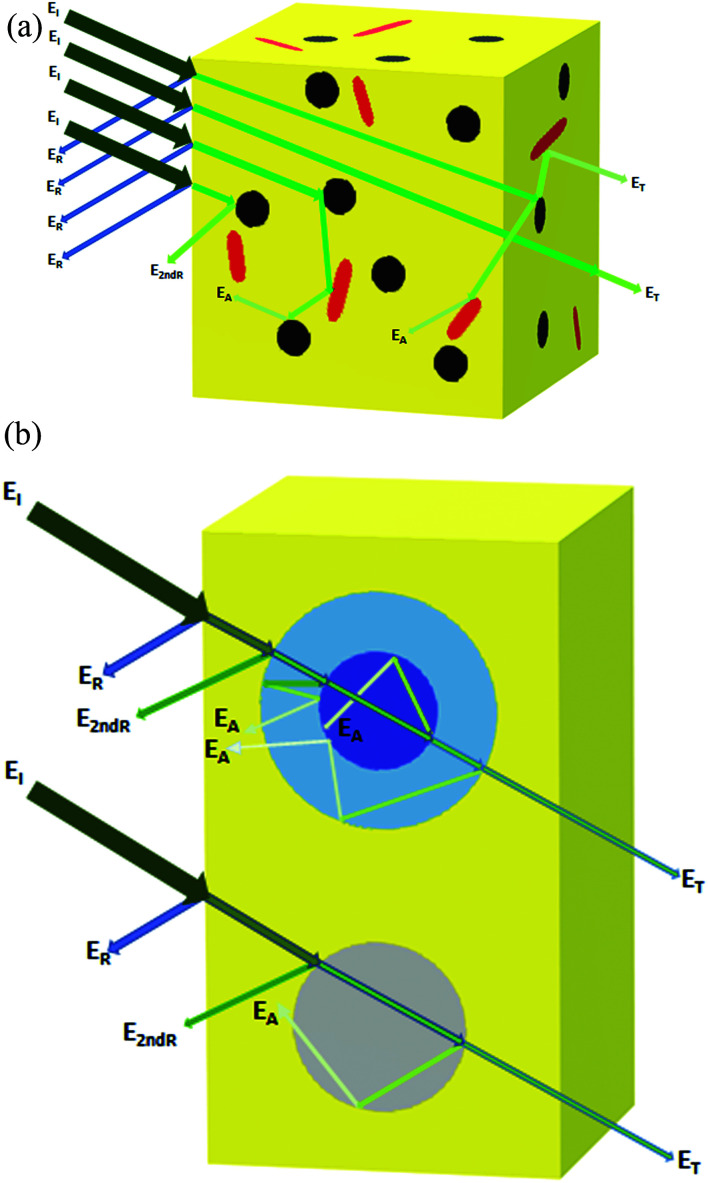
Schematic representation of the EMI shielding mechanisms for the composite materials filled with solid granular and fibrillar particles (a), and the composite materials filled with porous or hollow structures and shell structures (b).

When the electromagnetic radiation wave impacts the surface of the conductive material with an impedance that is different from that of the ambient environment, in which the electromagnetic wave propagates, the two waves are formed at the external surface: the reflected wave and the transmitted wave. The amplitude of both waves depends on the individual impedances of the shield and ambient environment. The strength of the penetrated wave is lowered and weakened due to its absorption by the shield. The absorbed energy is subsequently dissipated in the form of heat. As the attenuated electromagnetic wave reaches the second surface of the shield, a portion of the radiation passes through the shield, while the second portion is reflected back to the shield. If the shield is thicker than the skin depth, the wave reflected from the internal surface is absorbed by the shield, and the multiple reflection does not take place, or the loss due to it can be neglected. On the other hand, if the shield is thinner than the skin depth, the influence of the multiple reflection is significant in decreasing the overall EMI SE.^28^ The schematic representation of the shielding mechanisms for the thin plate shield is illustrated in Fig. 3. However, for porous materials and 3D structures including composite materials, the contribution of the multiple reflection is much more important. Fig. 4 provides a visualization of the shielding mechanisms in those materials. The high specific surface area, large phase interface, vacant space and voids give rise for the scattering, multiple reflection and absorption of the electromagnetic radiation.

The overall shielding efficiency (SE) in dB is the sum of the reflection (SE_R_), absorption (SE_A_) and multiple reflection (SE_MR_) of EMI:

3 EMI SE = SE_R_ + SE_A_ + SE_MR_

Several theories are used to calculate the shielding efficiency of the materials, including the plane-wave theory, near field shielding, metal foil, low-frequency magnetic field source and scattering parameters. Among them, the plane-wave theory is the most commonly used for the calculation of EMI SE based on the properties and thickness of the material.^37^ According to the plane-wave theory, the efficiency of reflection depends on the discrepancy of impedances of the incident electromagnetic wave and shielding material, and can be calculated as:4
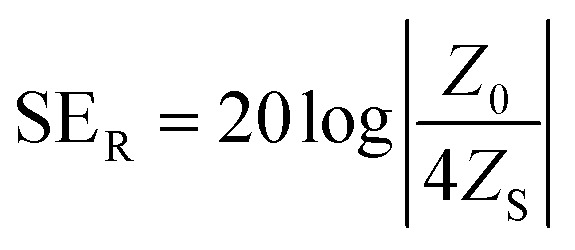
where *Z*_0_ is the impedance of the incident wave and *Z*_S_ is the impedance of the shield. From eqn (4), it can be deduced that the reflection shielding efficiency increases with decreasing impedance of the shield and increasing impedance of the electromagnetic wave. The impedance of the electromagnetic wave can be estimated based on the radiation frequency, distance from the source, and impedance of the source itself. In general, the impedance of the wave is indicated as high or low. As even medium conductive materials have a low value of shield impedance, most of the conductive materials exhibit very good ability to provide EMI shielding by reflection.

For absorption and multiple reflection, the following equations were formulated:

5 SE_A_ = 20 log^*t*/*δ*^

6 SE_MR_ = 20 log(1 − e^−2*t*/*δ*^)

In these equations, *t* is the thickness of the shielding material and *δ* is the skin depth. It becomes apparent that the absorption shielding efficiency increases with increasing thickness of the shielding material, while multiple reflection has a negative influence and decreases the overall shielding efficiency in the thin shield, in which *t* < *δ*. Multiple reflection becomes insignificant and generally neglected when the absorption loss SE_A_ is greater than 10 dB, which generally occurs at very high frequencies (∼GHz or even higher).^28,38–40^

As already outlined, the impedance of the electromagnetic wave *Z* is the ratio of the amplitudes of the electric and magnetic fields:7
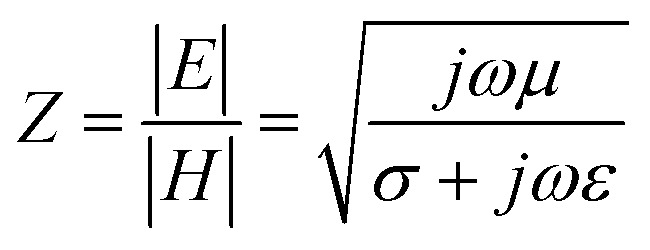
where *ω* is the angular frequency of the radiation, *μ* is the magnetic permeability, *σ* is the electric conductivity and *ε* is the permittivity of the medium. Providing that for the electromagnetic plane wave in the free space or vacuum *σ* = 0, eqn (7) can be rewritten as follows:8
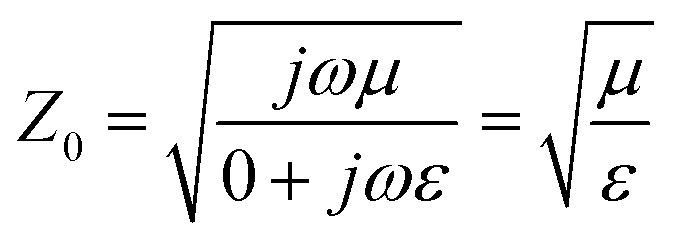


For the conductive materials *σ* ≫ *ωε*, therefore, the impedance of the shield *Z*_S_ can be calculated from eqn (7), in which *f* represents the frequency of the incident electromagnetic wave:9
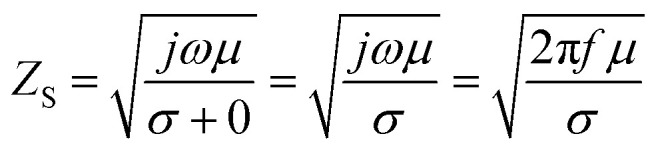


The shielding effectiveness by reflection and absorption can be then described as:10
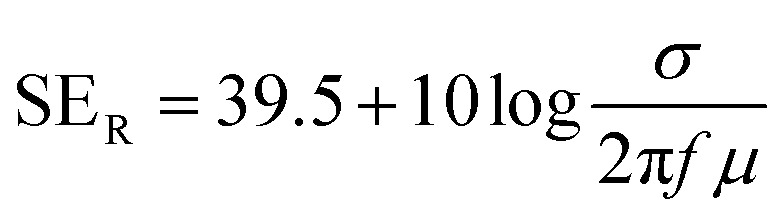
11
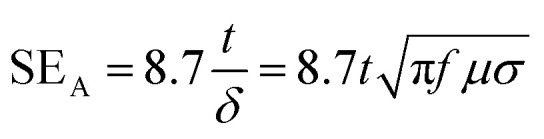


Based upon the outlined formulations, it can be stated that the efficiency of shielding by absorption is a function of *σμ*, whereas shielding by reflection is dependent on the ratio of both parameters *σ*/*μ*.^14,31,41,42^

Paul^43^ reported that the efficiency of the reflection shielding is the highest for highly conductive materials and low frequencies. By contrast, magnetic materials with permeability *μ* > 1 significantly reduced the shielding by reflection, and their dominant shielding mechanism becomes absorption. The efficiency of reflection decreases with increasing frequency equivalent to −10 dB per decade of frequency. For example, the shielding efficiency of conductive material from copper at 1 kHz is 138 dB. Nevertheless, with increasing frequency up to 10 MHz, the shielding efficiency falls down to 98 dB. On the other hand, the efficiency of shielding by absorption increases with increasing frequency according to the graphical dependence √*f* on the decibel scale. However, the situation is different for composite materials with heterogeneous structures, in which the polarization of the matrix, conductive filler and interfacial polarization can occur depending on the frequency range.^44–46^ These polarization sites lead to the formation of local fields, and lag of the displacement current relative to the conduction current.^47,48^ Fillers and matrices exhibit different electromagnetic properties. Therefore, the EMI shielding mechanisms of EMI shields can be understood by measuring their dielectric (relative complex permittivity *ε*_r_ = *ε*′ − *jε*′′) and magnetic (relative complex permeability *μ*_r_ = *μ*′ − *jμ*′′) properties. Parameter *ε*′ (real permittivity or dielectric constant) represents the electric charge storage capacity, whereas *ε*′′ (imaginary permittivity) indicates the dielectric dissipation or losses. Similarly, *μ*′ and *μ*′′ represent the magnetic storage and losses, respectively. The extent of losses can be calculated from the tangent of the dielectric loss (tan *δ*_E_ = *ε*′′/*ε*′) and magnetic loss (tan *δ*_M_ = *μ*′′/*μ*′).^49,50^

The permittivity and permeability have been reported to be crucial parameters that influence the EMI shielding efficiency. The complex permittivity constitutes the real component *ε*′ and imaginary component *ε*′′. The real permittivity of the filled composites is a measure of the amount of micro-capacitors and polarization centers.^45^ It is related to the displacement current and mainly affected by the polarization (localized charges) inside the material, and is correlated with the electrical charge storage within the composite. Imaginary permittivity, which is related to the dissipation of the electrical energy (dielectric loss), is mainly affected by ionic, orientational (dipole orientation), electronic and interfacial polarization. The ionic and orientational polarization is attributed to the bound charges in the material. The interfacial polarization originates from space charges, which accumulate owing to the dissimilarity in the electrical conductivity/dielectric constant at the interface of the two different materials.^51^ The enhanced dielectric permittivity of the composites can increase the dielectric loss contribution due to the difference in conductivity between the non-conductive polymer matrix and conductive filler. Due to this electrical conductivity mismatch between the matrix and the filler, composite materials may have polarization and charge accumulation at their interfaces. Thus, polymer composites with high dielectric values might act as good absorbers of electromagnetic radiation. In nanocomposites, conductive nanofiller networks act as dissipating mobile charge carriers. The increase in the number of conductive networks, as a result of increasing filler content, can lead to higher imaginary permittivity and finally to higher EMI dissipation by absorption.^52–54^ Following the free electron theory, the imaginary permittivity or dielectric loss is given by *ε*′′ = *σ*/2π*ε*_0_*f*, which indicates that the increasing conductivity shifts to the increase in imaginary permittivity. By contrast, the dielectric loss decreases with increasing frequency. The frequency-dependent dielectric response can be explained by the presence of electric dipoles. When the frequency of the applied field increases, the dipoles present in the system are not able to reorient themselves fast enough to respond to the applied electric field, which results in a decrease of the dielectric loss.^55^ The ionic and electronic polarization play a role only at very high frequencies (above 1000 GHz). Thus, their effects can be ignored in low microwave frequency region. Dipole polarization is led mainly by the presence of defects, voids and residual groups in the material, and it is influenced mainly by the production process, material types, and annealing temperature. The polarization ability of the composites can also be improved by the orientation and type of fillers in the matrix.^48,56^

Another important parameter is the material permeability. Materials with high permeability or magnetic loss have been reported to be good materials for shielding by absorption. The permeability of some materials changes with the change in intensity, temperature, and frequency of the applied magnetic field. The overall EMI SE also depends on the radiation frequency. It has been shown that the absorption shielding ability increases with the increase in radiation frequency. The magnetic loss results from hysteresis loss, domain wall loss, eddy current loss and residual loss. The hysteresis loss originates from the hysteresis (time delay of the magnetization vector *M* behind the magnetic field vector *H*), where the magnetic energy is dissipated in the form of heat.^57^ The eddy current loss can be neglected for highly conductive materials, and remains constant with the change in frequency. The magnetic loss also comes from the natural resonance and exchange resonance. The natural resonance usually takes place at lower frequencies, and nano-sized particles enhance the exchange resonance. Magnetic metals and their alloys (Fe, FeCo, FeNi) exhibit high saturation magnetization and good permeability. However, their conductive behavior generates eddy current losses, which results in reduced permeability at lower frequencies (typically in MHz range). On the other hand, ferrites rank among semi-conductive materials and have much lower saturation magnetization. Thus, natural ferromagnetic resonance takes place at the low GHz range. The above outlined aspects limit their utilization in the high GHz range to the maximum bulk ferromagnetic materials. To overcome the problem, research studies have been focused on nano- and micro-sized materials because the low-dimension materials mitigate eddy current loss. It can also be stated that the highly effective magnetic fields favor higher EMI absorption in the higher frequency range. The absorption shielding efficiency improves with lower *μ*′ value (magnetic storage) when compared to *μ*′′ (magnetic loss).^38,58^

Generally, in addition to the thickness of the shielding material, several other factors influence the overall EMI SE, including the electrical conductivity, permeability, permittivity, content, size, shape, aspect ratio and morphology of the filler/fillers applied. Among them, the electrical conductivity is one of the key parameters for effective shielding materials.^59^ In general, the electrical conductivity of composites increases with increasing amount of conductive filler incorporated in the polymer matrix. With increasing content of the filler, an interconnected filler network within the polymer matrix is formed, resulting in the sharp increase of the electrical conductivity. Thus, the content and the shape of the filler are the most important factors that influence the conductivity of composites. In contrast to spherical-type fillers, inclusions with fibrillar or tubular shape with a high aspect ratio can more easily connect together when dispersed within the polymer matrix at higher concentrations, forming a spatial filler network structure, leading to a significant increase of the conductivity. The geometric characteristics and the structure of the filler, such as the particle size, specific surface area, aggregating or agglomerating tendency also play important roles in the conductive network formation and overall EMI SE performance. The specific surface area and thus the aggregation tendency of the filler both increase with decreasing particle size. High structure fillers, for example carbon black, can more easily form a conductive filler network within the polymer matrices. Arjmand and co-workers^60^ synthetized silver nanowires (AgNW) by electrodeposition technique, and incorporated them into a polystyrene (PS) matrix through the solution-processing approach. Polystyrene/multiwalled carbon nanotube (PS/MWCNT) composites were fabricated following the same procedure for comparison. It was shown that AgNW developed into chain nanospheres by the fragmentation phenomenon at molding temperature (240 °C). The results demonstrated that at low filler loading, the electrical conductivity of PS/AgNW was inferior compared to that of the PS/MWCNT composites. This can be attributed to the higher particle size of AgNW (10 nm diameter for MWCNT, 25 nm diameter for AgNW), and thus a lower specific surface area, lower aspect ratio, fragmentation phenomenon and inferior conducting network of AgNW. On the other hand, at high filler loading, the intrinsic conductivity of AgNW overcame the lower aspect ratio and worse conducting network. This resulted in the better electrical characteristics of the PS/AgNW composites. The results also demonstrated a very close correlation between the conductive network formation and EMI shielding performance. Ram *et al.*^61^ investigated the influence of three different nano-carbon based fillers, namely carbon black, types N472, N550 and N774 on the electrical conductivity and EMI SE of poly(vinylidene fluoride) (PVDF) based polymer composites. All three types of carbon black exhibited different particle size and structure, and thus also different aggregating tendency. The results revealed that the electrical conductivity of the composites increased as following: PVDF/N774 < PVDF/N550 < PVDF/N472. The different behavior can be attributed to the different particle size and structure of the tested fillers. Finally, the authors stated that carbon black type N472 with the smallest particle size, the highest specific surface area and the highest structure imparted the highest electrical conductivity and the highest overall EMI SE to the corresponding composites. In addition to the filler size and morphology, the filler synthesis conditions can also affect the electrical properties of the composite materials. For example, in the study performed by Arjmand *et al.*,^62^ it was demonstrated that the synthesis catalyst Co, Fe and Ni strongly influenced the electrical properties and crystalline structure of the nitrogen-doped carbon nanotubes (N-CNT) by chemical vapor deposition method. The synthesized N-CNT were subsequently melt compounded with the PVDF matrix. The application of Ni and Fe resulted in the formation of N-CNT with bamboo-like configurations, whereas N-CNT fabricated by application of Co was open-channel. The filler aspect ratio was the highest by using the Co catalyst, followed by Ni and Fe. On the other hand, the introduction of the Ni catalyst resulted in the highest nitrogen content for N-CNT. The electrical conductivity and EMI SE of the N-CNT composites prepared by application of different catalysts increased in the order: Co > Fe > Ni. The higher EMI shielding performance of the N-CNT nanocomposites fabricated by the Co-catalyst was attributed to the combination of the high synthesis yield, high crystallinity of N-CNT, high aspect ratio of the filler, low structural defects, low nitrogen content, as well as good filler dispersion state in the composite. It was also clearly revealed that the high aspect ratio along with the good filler dispersion state resulted in a low percolation threshold and improved conducting network formation. This was subsequently reflected in the higher shielding efficiency of the nanocomposites.

## 3 Microwave electromagnetic absorption properties

As already outlined, three mechanisms contribute to the overall efficiency of the electromagnetic radiation shielding, namely the reflection, absorption and multiple reflection of EMI. However, in many cases, shielding by reflection is undesirable due to the secondary interference of the reflected EM radiation with the initial radiation emitted from other electromagnetic sources, causing a secondary EMI effect. Therefore, materials that are able to shield EMI by absorption have attracted greater interest over the last decades. The microwave absorption properties of the materials in terms of the reflection loss or return loss RL in dB can be expressed by the equation:12
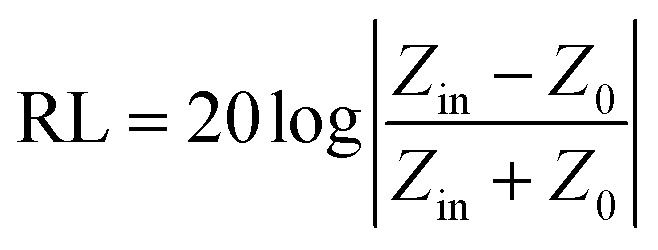
where *Z*_in_ is the input impedance of the shield and *Z*_0_ is the impedance of free space (*Z*_0_ = 377 Ω). The maximum absorption occurs when reaching the minimum reflection loss (RL_min_). This means that the input impedance of the absorber and impedance of free space is matched (*Z*_in_ = *Z*_0_ = 377 Ω). The input shield impedance backed by a metallic reflector can be expressed as follows:13
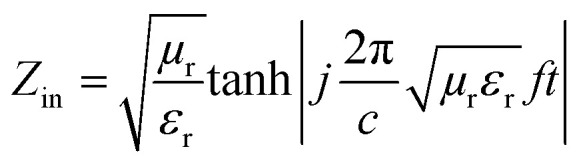
where *μ*_r_ and *ε*_r_ are the complex permeability and complex permittivity of the shield, respectively, *c* is the velocity of light, *f* is the frequency of electromagnetic radiation wave, and *t* is the thickness of the EMI absorber. The EM absorption ability of the EMI shield can be confirmed by calculating the attenuation constant (*α*) using the following equation:14
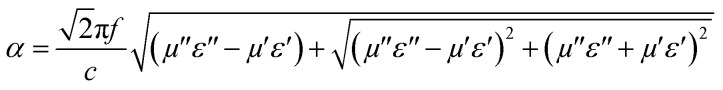


Based upon the outlined aspects, it becomes apparent that the microwave absorption of the shield can be adapted by impedance matching. The impedance mismatch between the shield and free space can be minimized by proper balance between the dielectric loss (tan *δ*_E_) and magnetic loss (tan *δ*_M_). The best electromagnetic matching for zero reflection would be reached under the condition tan *δ*_E_ = tan *δ*_M_. However, this condition cannot be achieved for the shielding material within the whole frequency range, and thus the microwave absorption of the shield is frequency-dependent. It also becomes obvious that the higher values of *μ*′′ and *ε*′′ could induce a higher *α* value. The absorption shielding materials are able to convert the electromagnetic energy into thermal energy trough dielectric loss and/or magnetic loss by the balance outcome integralities between the relative permittivity and/or relative permeability.^48^

It has been reported that the shielding materials with RL at the level of −10 dB can absorb 90% of the incident electromagnetic radiation, and this is considered to be a sufficient effectiveness for the majority of common applications.^63,64^ Materials with a shielding efficiency equivalent to −20 dB are able to absorb 99% of the incident electromagnetic radiation. Thus, the prepared composites must provide a return loss of at least −10 dB and less to be sufficiently effective as EMI absorbers.

The electrical conductivity of highly conducting materials (*σ*) seems to be the only key factor for shielding effectiveness owing to their high tan *δ*_E_ (higher than 1). On the other hand, in materials showing poor conductivity (tan *δ*_E_ is much lower than 1), both permittivity and electrical conductivity are important characteristics for intrinsically conductive polymers and polymer composites.^59^ Therefore, adjusting the electromagnetic properties and thickness of the EMI shields is important to obtain satisfactory shielding and to regulate the appropriate shielding mechanism.

There are also some other requirements for efficient EMI shields, such as lightweight property, corrosion and chemical resistance, having minimum thickness, good elasticity and flexibility, applicable mechanical properties, ease of processing and production, and an acceptable price.^38,48^

## 4 Experimental determination of the EMI shielding efficiency

**Fig. 5:**
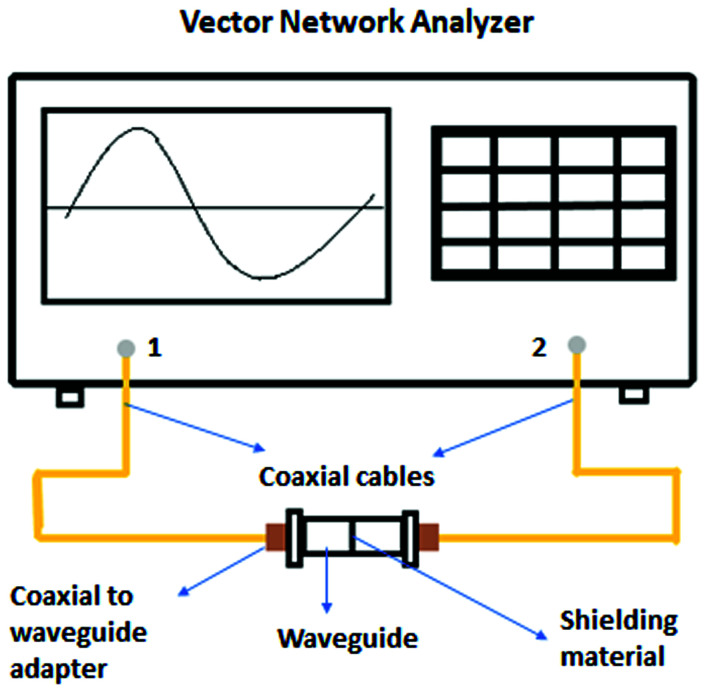
Vector network analyzer.

**Fig. 6:**
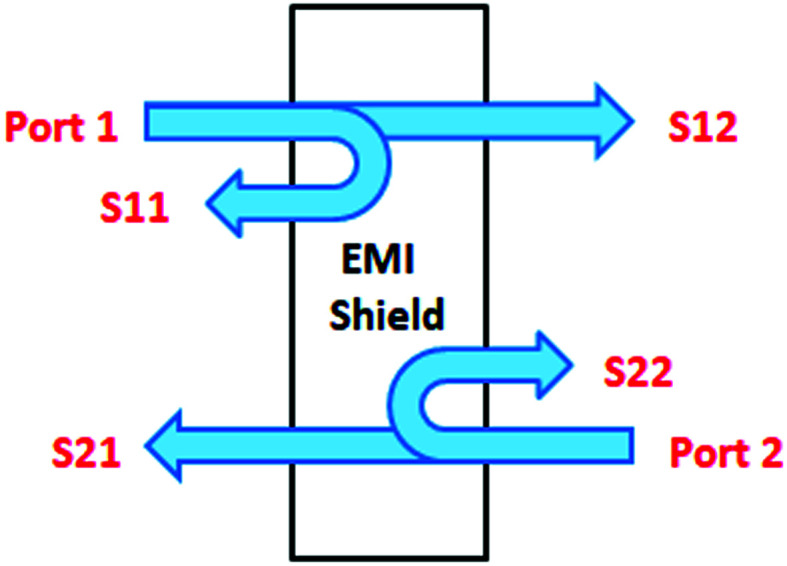
Schematic illustration of the scattering parameters from a two-port VNA.

Experimental instruments called network analyzers are introduced for the evaluation of EMI SE. Two types of network analyzers, which operate on the principle of the waveguide technique, can be applied: a scalar network analyzer (SNA) and vector network analyzer (VNA). The scalar network analyzer (SNA) determines the amplitude of signals, while the network analyzer VNA detects the signal magnitude response, as well as phases of various signals. As SNA cannot be used for the determination of complex signals (complex permeability or permittivity), VNA is the more preferred instrument, despite its higher price.^59^ VNA containing two ports emits electromagnetic radiation in the examined frequency range from both ports and records the reflected radiation, as well as the transmitted radiation obtained from the tested shielding material (Fig. 5). According to the EMI shielding theory, when the electromagnetic propagating wave reaches the surface of the shielding material, the incident power is divided into the reflected, absorbed and transmitted power, and the corresponding power coefficients of absorbance (*A*), reflectance (*R*), and transmittance (*T*). Their sum is always equal to 1, and this means that *R* + *T* + *A* = 1. VNA detects the complex scattering parameters (*S*-parameters). Based on these parameters, information about the permittivity, permeability, as well as EMI SE can be obtained using suitable algorithms and models, such as Nicolson–Ross–Weir (NRW), NIST iterative, new non-iterative, short circuit line (SCL) techniques.^65^ Among them, the Nicolson–Ross–Weir technique is the most widely used regressive/iterative analysis, as it provides the direct calculation of both permittivity and permeability from the *S*-parameters. There are experimentally measured real and imaginary parameters *S*_11_ (or *S*_22_) and *S*_21_ (or *S*_12_), which correlate with the reflection coefficient (*R*) and transmission coefficient (*T*), *R* = |*S*_11_|^2^ = |*S*_22_|^2^ and *T* = |*S*_12_|^2^ = |*S*_21_|^2^, respectively. The absorption coefficient can be calculated as *A* = 1 − *R* − *T*. Fig. 6 illustrates the *S*-parameters from the two-port vector network analyzer (VNA), which represent the incident and transmitted EM waves. The *S* parameters are designated as the forward reflection coefficient (*S*_11_), reverse reflection coefficient (*S*_22_), forward transmission coefficient (*S*_12_) and backward transmission coefficient (*S*_21_). The corresponding parameters *Z* (Ω), RL (dB), SE_A_ (dB), SE_R_ (dB), and SE_T_ (dB) can be calculated as follows:^66–68^15
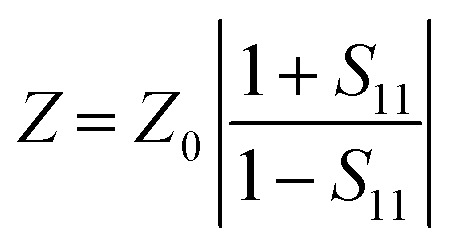

16 RL = 20 log|*S*_11_|

17
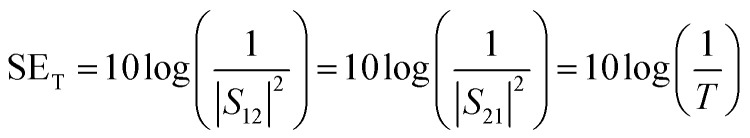
18
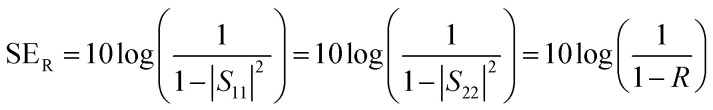
19
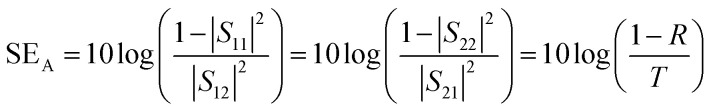


A better understanding of the absorption by the EMI shield can be achieved by calculating its effective absorption (*A*_eff_) percentage using equation:^38,39^20
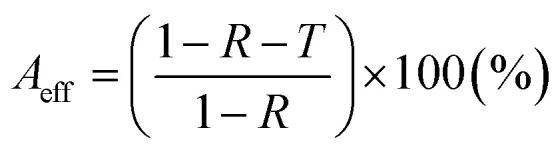
where *A*_eff_ represents the amount of the power absorbed by the shield.

## 5 Materials used for EMI shielding

For designing a material for a particular shielding application, it is important to have an in-depth knowledge of both intrinsic and extrinsic parameters, on which the shielding effectiveness depends, along with suitable theoretical relations correlating them with the reflection, absorption and multiple-reflection loss components. The careful analysis of the theoretical shielding expressions revealed that in order to meet the design requirements and for extending the efficient shielding performance, the shielding material should exhibit a balanced combination of the electrical conductivity (*σ*), dielectric permittivity (*ε*), and magnetic permeability (*μ*), as well as a suitable physical structure and geometry.^69^

### 5.1 Intrinsically conductive polymers (ICP)

**Fig. 7:**
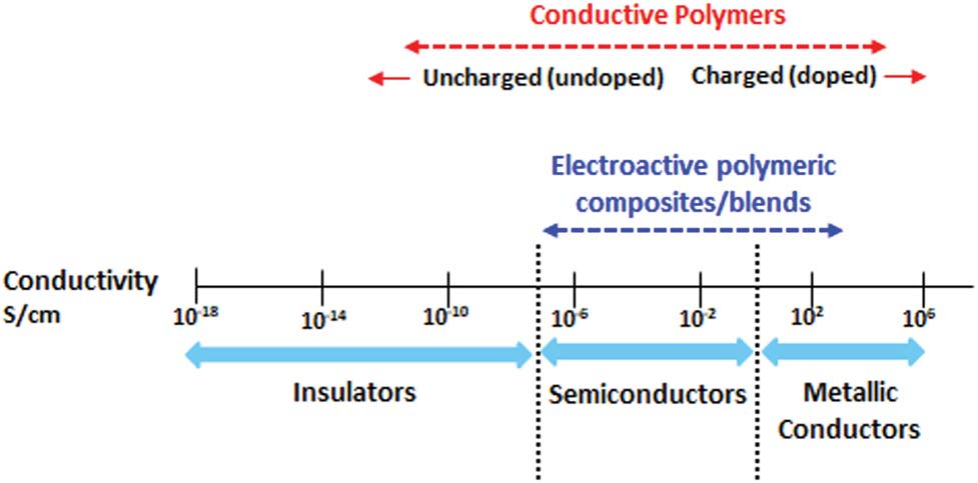
General conductivity range of conductive polymers.

**Fig. 8:**
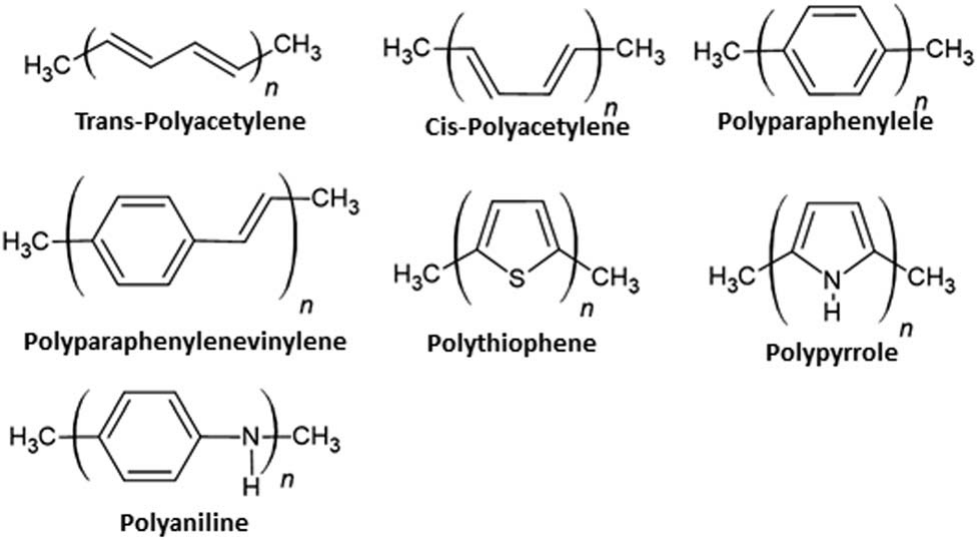
Chemical structures of conductive polymers.

Despite the fact that polymers are generally considered to be an electrical insulator, there are some polymers with increased conductivity in the doped states. In general, ICP possesses alternating single (*σ*) and double (π) bonds, and these conjugated systems with the presence of π-electrons lend the ICP their inherent optical, electrochemical, and electrical/electronic properties.^70^ The π-electrons in the conjugated backbone are available to delocalize into a conduction band. In the idealized situation of a uniform chain, the resulting conduction band would give rise to metallic behavior. However, this system is unstable with respect to the bond alteration, which causes the formation of an energy gap in the electronic spectrum. Dopant ions are incorporated into the structure to overcome the energy gap, and thus to impart conductivity to those polymers. Conductive polymers have been doped using different methods in order to achieve high conductivities. Dopants in the polymers undergo redox processes, in which the charges are transferred with the subsequent formation of charge carriers. The role of the dopant is not only to withdraw electrons from polymer chains, but also to add electrons to the polymer backbone. These oxidation/reduction processes create charge carriers in the form of polarons (radical ions), bipolarons (dications or dianions), or solitons in the polymer. The ability to control their electrical conductivity by adjusting parameters, such as the oxidation state, doping level, dopant ion size, protonation level, morphology and chemical structure makes them suitable candidates for EMI shielding in various technological applications.^71^ The shielding efficiency of intrinsically conductive polymers arises from mobile charges (polarons, bipolarons, solitons) and bound charges (dipoles) at their backbone.^24,72,73^ When compared to solid metals, ICP exhibits low density, ease of processing and preparation conditions, easy-to-control shape and morphology, corrosion resistance, structural flexibility, and tunable electrical conductivity. For instance, iodine-doped polyacetylene shows an electrical conductivity of around 1.7 × 10^5^ S cm^−1^, which is comparable to some metals.^60^ On the other hand, as with many other polymers, they undergo thermo-oxidative aging and other forms of degradation. However, due to the low hydrogen content and aromatic structure, their thermal, chemical and thermo-oxidative stability are very high. They also suffer from swelling in some solvents, cracking or contraction, which may have a negative impact on their electrical and mechanical properties. Those properties can be enhanced by the addition of a second phase, mainly in the form of fillers, such as metallic or magnetic particles, metal oxides or various carbon-based fillers. Those fillers can improve their mechanical properties (mostly carbon based fillers), thermal stability, dielectric or magnetic properties and electrical conductivity, and thus improve their overall shielding efficiency. A lot of nanocomposites containing carbon-based fillers, such as carbon nanotubes, graphene, carbon nanofibers or carbon nanotubes, have been prepared and examined.^74–76^ These carbon fillers improved the structural ordering of the polymer chains and facilitated the delocalization of the charge carriers, resulting in enhanced conductivity. The shielding mechanism of the conductive polymers is usually based on the combination of refection and absorption, rather than reflection, which is the dominant shielding mechanism of metals.^48^ The intrinsic electrical conductivity of the conjugated polymers in the microwave absorption band (100 MHz to 20 GHz) makes them very promising materials.^77^ In addition, conducting polymers belong to the group of few materials, which are able to exhibit dynamic (switchable) microwave absorption behavior. These materials are marked so-called “intelligent stealth materials”, because they exhibit the reversible electrical properties of conducting polymers, which are influenced by redox doping/de-doping processes. Intrinsically conductive polymers mainly include polyaniline (PANI), polypyrrole (PPy), polythiophene (PTH), polyfuran (PF), polyacetylene (PA), polyparaphenylene (PPP), poly(*p*-phenylene vinylene) (PPV), and poly(3,4-ethylenedioxythiophene) (PEDOT). They have been successfully used in many applications, such as chemical sensors, electro-magnetic shielding, electrically conducting fibers, corrosion inhibitors, antistatic coatings and in “smart” windows that can regulate the amount of light passing through them. Some of the most exciting potential utilizations of these materials include compact electronic devices, such as polymer-based transistors, lasers and light-emitting diodes. These electronic devices can find novel applications in the electronic industry, for example in flat flexible television screens or as acceptors in polymeric solar cells. The undoped forms of those polymers exhibit poor conducting characteristics lying in the semiconductive or insulating range (10^−10^ to 10^−5^ S cm^−1^). However, the controlled doping of the poorly conductive and undoped polymers can produce materials that are within the range of those with semiconductive or metallic conductive characteristics (up to 10^5^ S cm^−1^). The conductivity of the majority of conjugated polymers reaches maximally up to 10^3^ S cm^−1^.^24,77^ The conductivity range of the conjugated polymers and conductive polymer composites is displayed in Fig. 7.^78^ The chemical structures of some conductive polymers in their undoped forms are presented in Fig. 8. Among them, polyaniline and polypyrrole are the most studied.

Polyaniline exhibits various configurations, strong environmental stability, ease of synthesis, tunable electrical and optical properties, simple regulation of conductivity by changing of the oxidation and protonation states, and special doping mechanisms.^79^ PANI can exist in various forms based on its oxidation level, as the fully oxidized pernigraniline base, half-oxidized emeraldine base, and fully reduced leucoemeraldine base. The most stable and conductive configuration is the PANI emeraldine form. Therefore, it is used in conductive polymer membranes, conductive adhesives, antistatic coatings, anticorrosive materials, energy storage batteries, microwave absorbers, and as EMI shields.^72,79,80^ PANI in different forms, such as thin films, multilayer films, fabrics, adhesives, nanoparticles or composites, has been investigated for its shielding efficiency in the MHz and GHz frequency ranges. The overall EMI SE ranged from 16 to 50 dB within the 0.1–1 GHz frequency scale. When applying higher frequencies (1–18 GHz), the shielding efficiency within the range from 31.9 to 81 dB has been recorded.^81,82^ Functionalization of the PANI emeraldine base with protonic acids has become the conventional method of doping. Protonic acids oxidize the conducting polymer chain, and polyaniline becomes conductive by the protonation of nitrogen. Sasikumar *et al.* investigated polyaniline doped with camphor sulfonic acid (PANI-CSA) as EMI shields in the S, X and Ku microwave frequencies ranging from 2 to 18 GHz.^83^ The shielding films were produced using the standard procedure with CSA as a dopant and *m*-cresol as a solvent. This film provided nearly 45 dB of shielding in a broad band frequency range, which is favorable for its use in commercial applications. Studies also showed that the shielding efficiency of the film increased by application of *m*-cresol, which behaved not only as a solvent but also as a secondary dopant. Qiu *et al.* revealed that the application of phosphoric acid leads to the formation of nanofiber-like morphology, whereas hydrochloric acid and camphor sulfonic acid form holothurian-like structures for PANI.^84^ Among all three samples, the camphor sulfonic (CSA)-doped PANI exhibited the highest electrical conductivity (about 1.28 S cm^−1^) due to the large oxidation extent, crystallinity and size of the crystallites. An EMI shielding efficiency of around 20 dB of PANI-CSA was obtained with the thickness of only 0.35 mm. All tested samples showed an absorption-dominated shielding mechanism. Chandrasekhar *et al.* examined the microwave shielding capability and absorption of polyaniline doped with two proprietary sulfonate dopants, and they found that bulk polyaniline exhibits good shielding performance (≤15 dB over 4 to 18 GHz) and poor absorption capability (about −5 dB in the X and K bands).^85^

Polypyrrole is a very promising conducting polymer, which shows interesting electrical properties. It is thermally and chemically stable, and easy to prepare. However, PPy suffers from poor mechanical strength and processability problems, together with insolubility and infusibility.^86^ Like many other fully aromatic polymers, polypyrrole is an electrical insulator. However, when oxidized, it becomes an electrical conductor. The conductivity of PPy strongly depends on the preparation procedure. Furthermore, the conductivity of PPy films can achieve up to 10^3^ S cm^−1^, depending on the type and amount of the dopant.^87^ When suitable fillers are applied, the conductivity can be increased by about two orders of magnitude in comparison with a virgin PPy. It can be used as an anti-electrostatic coating, gas sensor, solid electrolytic capacitor, and as a component in many other electronic devices.

Poly(*para*-phenylene) (PPP) is another interesting polymer that exhibits unusual electronic and optical properties, and is electrically conductive when doped. This polymer is very stable up to temperatures of about 500 to 600 °C with a minimal or only slow oxidation. It is insoluble in most solvents and has a very high melting point. It can be processed into a crystalline thin film, for example by vacuum deposition. PPP is photoconductive and has potential for electroluminescence applications, such as light-emitting diodes.

Poly(*para*-phenylene vinylene) (PPV) and its derivatives are another important class of conductive polymers that have been widely studied because of their interesting and potentially useful optical and photo-electronic properties. The phenylene units in polyphenylene vinylenes are connected to one another through carbon–carbon double bonds, resulting in a rigid, rod-like linear polymer consisted exclusively of double bonds and aromatic rings. It can be processed into a highly ordered crystalline thin film that is electrically conductive upon doping. Like PPP, PPV is capable of electroluminescence, and can be used as an emissive layer in polymer-based organic light-emitting diodes such as those used for electroluminescent displays. PPV and its copolymers are also used as efficient acceptors in polymeric solar cells.

Poly(3,4-ethylenedioxythiophene) (PEDOT) is a conductive polymer with the biggest prospects in the field of bioelectronics and microwave applications due to the combination of attributes as conductivity, stability, biocompatibility and transparency. It exhibits a moderate band gap, attractive electrochemical activity and controllable electrical conductivity. It is easy to prepare with good environmental stability. Its dielectric loss characteristics make it promising as a microwave absorbing material.^48^

Another highly examined material is poly(3,4-ethylenedioxythiophene):polystyrenesulfonate (PEDOT:PSS). It is used for the production of films for EMI shielding applications, but also for the production of highly stretchable conductors in electronics. Wang *et al.*^88^ demonstrated a method for making highly stretchable and conductive PEDOT:PSS films (higher than 4100 S cm^−1^ under 100% strain) with cycling stability by the incorporation of ionic additives. Having a conductivity of over 3100 S cm^−1^ under 0% strain and over 4100 S cm^−1^ under 100% strain, they belong to the highest reported for stretchable conductors. They were found to be highly durable under cyclic loading with the conductivity remaining at 3600 S cm^−1^ even after 100 cycles to 100% strain. The conductivity maintained above 100 S cm^−1^ under 600% strain, with a fracture strain of 800%, which is better than even silver nanowires or carbon nanotube-based stretchable conductor films. Highly conductive and stretchable polymer films prepared by blending of PEDOT:PSS with highly stretchable waterborne polyurethane (WPU) were examined by Li *et al.*^89^ They revealed that with increasing content of PEDOT:PSS, the conductivity of the films increased, while the stretchability decreased. At 20 wt% of PEDOT:PSS loading, the films exhibited a conductivity of 77 S cm^−1^ and elongation at a break of around 33%. With a film thickness of only 0.15 mm, they showed high EMI shielding efficiency at about 62 dB over the X-band frequency range. In the study,^90^ a facile dip coating design has been adopted to fabricate the PEG/PEDOT:PSS treated fabric. The high electrical conductivity of 51 S cm^−1^ and EMI SE of 46.8 dB (over the X-band) were achieved by only 20 dipping cycles (thickness ∼0.38 mm), whereas the optimum conductivity and shielding were found to be 82.7 S cm^−1^ and 65.6 dB at 25 dipping cycles. The improvement in the electrical conductivity was shown to be the phase separation between PEDOT and PSS, followed by the charge screening phenomena. The exclusive “void-filler” inclusion in the whole fabric was examined in order to correlate the microstructure (both 2D and 3D), along with the desirable conducting parameters. The conductive network remains flexible even after prolonged sunlight exposure, repeated bending with twisting, thermal air ageing, peeling with single sided tape, boiling with water and different organic solvents, which proves its versatile chemical and mechanical robustness. The results demonstrated that such long-term deformations exhibit above 95% retention of the overall shielding efficiency.

Polyacetylene is a rigid, rod-like polymer, which consists of long carbon chains with alternating single and double bonds between the carbon atoms. It is a well-known conductive polymer as its discovery started the development of (doped) highly conductive polymers. It was synthesized for the first time in 1974 by Hideki Shirakawa, Alan Heeger, and Alan MacDiarmid from acetylene by application of the Ziegler–Natta catalyst. Despite its metallic appearance, the first effort did not lead to the formation of a conductive polymer. However, three years later, they found out that oxidation with halogen vapor resulted in a conductive polyacetylene film, which exhibited much higher conductivity than any other previously known conductive polymers. The conductive polyacetylene polymer was produced by the exposure of polyacetylene to dopant compounds, oxidizing or reducing agents, electron-donor or electron-receptor of electrons. Although the development of the conductive polymers started with the discovery of polyacetylene, it has no practical commercial applications.

### 5.2 Composites based on ICP

In addition to the effort to influence the electrical properties and conductivity of ICP by designing their microstructure, there has been increased interest for the preparation of heterogeneous materials by the incorporation of other additives, mostly different types of carbon-based fillers, as well as inorganic fillers, such as metals, metal oxides, and magnetic ferrites. The incorporation of fillers results in the modification of processing characteristics, physical–mechanical properties, and in the manipulation of electric and electromagnetic properties. On the other hand, the high level of filler loading can be detrimental for the processability, density, surface quality and physical–mechanical properties of composites. The utilization of ICP as a matrix can be attributed to its advantages, such as design flexibility, good filler incorporation-ability, specific interactions with fillers and microwave non-transparency.^69^ When conducting fillers (metal particles, carbon black, CNT or graphite, *etc.*) are incorporated into undoped (poorly conductive) ICP matrices, the electrical conductivity of the composites enhances and follows the typical percolation behavior. On the other hand, the addition of conductive fillers into the doped (intrinsically conductive) ICP matrices results in further increase of the electrical conductivity and higher shielding performance. This may be attributed to the bridging of the metallic islands of ICP (granular metals), as well as better dispersion of ICP-coated fillers within the matrices.^77^ The increase in the conductivity is strongly dependent on the nature, type, concentration and aspect ratio of the filler particles, as well as the type and morphology of ICP matrix.

Metal nanoparticles, such as iron, copper, nickel and their related alloys generally exhibit high saturation magnetization, compatible dielectric loss and distinguishable permeability in the gigahertz frequency range. Polypyrrole (PPy)/polydopamine (PDA)/silver nanowire (AgNW) composites with high electromagnetic interference shielding performance, light weight and good adhesion ability were fabricated *via in situ* polymerization, and were examined by Wang *et al.*^91^ The incorporation of AgNW imparted the functionalized PPy with tunable electrical conductivity and improved EMI shielding efficiency. By increasing the content of AgNW in composites from 0 to 50 wt%, the electrical conductivity significantly increased from 0.01 to almost 1207 S cm^−1^, and the EMI SE of the composites changed from 6.5 to 48.4 dB (8–12 GHz). Dong *et al.* and Xu *et al.* prepared PANI/Ni and PPy/Ni nanocomposites *via* the *in situ* chemical oxidative polymerization of the monomer in the presence of Ni powder, respectively.^92,93^ The resultant PANI/Ni and PPy/Ni composites exhibited natural magnetic resonance and multiple dielectric relaxations. Due to the synergetic consequence of the Ni cores and polymer shells, the multiple relaxations for the enhanced dielectric loss induced by the ICP coatings and proper electromagnetic impedance matching contributed to the improvement of the EMI absorption. The same method can be used for the production of the PPy/Co nanocomposite. The composite with a thickness of 3 mm showed microwave absorption efficiency within the 10–18 GHz frequency range.^94^

Application of metal oxides in ICP matrices leads to the improvement of their dielectric properties. The metal oxide-formed modified phase at the interface restricts the mobility of the charge carriers in the polymer chains of ICP. The accumulation of charges enhances the capacitance of composites, and thus increases the dielectric constant. When compared to metal fillers, metal oxides exhibit lower corrosion resistance and higher thermal stability.^38^ Patil *et al.*^95^ prepared PANI composites by *in situ* polymerization method with the dispersion of lead oxide (PbO). Microwave properties, such as permittivity, return loss and EMI SE of composites were investigated within the frequency range of 8–12 GHz (X-band frequency), and the maximum EMI SE was observed by application of 10 wt% PbO. The *in situ* synthesis was also used for the preparation of PANI/MnO_2_ nanorod composites, and the freestanding films were prepared by solution processing.^96^ The EMI shielding properties of the prepared films were examined in the X-band and Ku-band frequency range. Outstanding EMI SE was observed for the films in both frequency bands. In the X-band, ∼35 dB (169 ± 3 μm) and ∼26 dB (81 ± 3 μm) EMI SE were achieved for the films, and this was increased up to ∼39 dB and ∼27 dB in the Ku band. EMI shielding due to absorption was found to be dominant for both frequency bands. In the work,^97^ PPy/Al_2_O_3_ nanocomposites were fabricated by chemical polymerization of pyrrole in the presence of Al_2_O_3_ particles using iron trichloride (FeCl_3_) as an oxidant. During this process, the aluminium oxide cores were covered by a thin PPy shell. The prepared nanocomposite was mixed with a resin and coated on a textile layer with a thickness of 0.6 mm. The shielding effectiveness of the nanocomposite-coated fabric measured in the 8–12 GHz frequency range was at the level of −3.3 dB (53%). However, this is only moderate attenuation value for industrial applications.

Carbon nanostructures have been frequently used as fillers or a second phase in ICP matrices due to their lightweight property, excellent conductivity, high permittivity, high thermal stability or well-regulated aspect ratio. Incorporation of carbon-based materials into ICP may reinforce the dielectric loss properties, and provide additional dielectric loss mechanisms (*e.g.*, various polarizations). Wu *et al.* examined the influence of the carbon black content on the EMI shielding characteristics of PANI, and demonstrated that PANI/CB (20 and 30 wt%) composites showed the absorption-dominant mechanism over the radar frequency band (2–40 GHz).^98^ Saini *et al.*^69^ prepared highly conductive polyaniline/multiwalled carbon nanotubes nanocomposites by *in situ* polymerization. SEM and TEM analysis demonstrated a uniform coating of PANI on the surface of individual MWCNT particles. The electrical conductivity of PANI/MWCNT composites (19.7 S cm^−1^) was even higher than MWCNT (19.1 S cm^−1^) or PANI (2.0 S cm^−1^), which can be attributed to the synergistic effect of both phases. The absorption-dominated total EMI SE of −27.5 to −39.2 dB of these composites points out the ability of these materials to be used for microwave shielding in the Ku-band (12.4–18 GHz). A similar research study refers to the preparation synthesis and characterization of highly conductive PPy/MWCNT nanocomposites.^99^ The composites were prepared by *in situ* polymerization of pyrrole using 5-sulfoisophthalic acid monolithium salt (lithio-sulfoisophthalic acid) as a dopant and ferric chloride as an oxidant. The electrical conductivity of the composites showed an increasing trend with increasing amount of MWCNT up to 52 S cm^−1^ at 5 wt% content of MWCNT. The EMI shielding efficiency also showed a similar tendency, and an average EMI SE of −108 dB was observed over the X-band frequency range. Absorption was the dominant shielding mechanism (from −93 to −108 dB). Sarvi and Sundararaj^100^ reported that the *in situ* polymerization of aniline in the presence of MWCNT leads to the preparation of core–shell nanostructures, which can be used in polymer conductive composites. The conductivity of the core–shell nanostructures can be adjusted by altering the thickness of the coating. Core–shell PANI/MWCNT nanofibres were dispersed in polystyrene (PS) using solution mixing. In comparison with PS/MWCNT composites, PANI/PS/MWCNT composites showed electrical percolation at much lower concentration. When used as a coating layer, PANI is able to attenuate EM radiation by absorption. Dielectric measurements of the PANI/PS/MWCNT composites showed a one order of magnitude increase in the real part of the electrical permittivity when compared to PS/MWCNT composites, which enables them to be used for charge storage purposes. Zakar *et al.*^101^ investigated the cumulative effects of layering a conductive polymer composite containing 2 wt% of MWCNT in PEDOT:PSS on a Mylar substrate for application in EMI shielding. The optical transmittance of the spin-coated composite layers was 90%, 45%, and 20% with a thickness of 0.05 μm, 0.15 μm, and 0.45 μm, respectively. The addition of isopropyl alcohol to the solution mixture and substrate heating to 40 °C resulted in the increase of conductivity, and subsequently to the significant increase in overall EMI SE (21 dB within a narrow frequency range in the Ku-band). In the work,^102^ highly flexible polyvinyl alcohol/poly(3,4-ethylenedioxythiophene)–polystyrenesulfonate/multiwalled carbon nanotubes (PVA/PEDOT:PSS/MWCNT) freestanding composite films were fabricated by solution mixing process, followed by simple solvent casting technique. PVA/PEDOT:PSS/MWCNT composite films of thickness around 20 μm showed high EMI SE over the X-band frequency range (60 dB with outstanding absorption-dominated shielding mechanism) and extensive mechanical strength. In the study,^76^ highly conductive polypyrrole/graphene (PPy/GN) nanocomposites were prepared by *in situ* polymerization with different contents of functionalised GN (1%, 3% and 5%). The results demonstrated a significant interaction between the phases. The PPy/GN nanocomposites showed a semiconducting behaviour similar to that of PPy, as well as enhanced dielectric and EMI shielding characteristics. The dielectric constant and overall EMI SE of the nanocomposites showed an increasing trend with increasing content of GN, and were found to be absorption-dominated. This indicates that the PPy/GN nanocomposites can be potentially used as lightweight EMI shields for the protection of electronic systems from EMI in the Ku-band. Shakir *et al.*^103^ used thermally reduced graphene oxide (TRGO) as a nano-level reinforcement to improve the EMI SE of the polymer blends of polyaniline and polyvinyl chloride. The EMI SE of PVC, PVC/PANI blend and PVC/PANI/TRGO hybrid nanocomposites was investigated in the microwave frequency region of 11–20 GHz. The PVC/PANI blend reached the shielding effectiveness of almost 29 dB. When 5 wt% of TRGO was added, the EMI SE was enhanced by up to ∼56 dB. In the study,^104^ Shakir *et al.* prepared polymer blends based on PVC and PANI with the inclusion of graphene nanoplatelets (GNP). EMI shielding measurements of the PVC/PANI/GNP composites were evaluated within the frequency range of 10 MHz to 20 GHz. In the case of PVC/PANI (15 wt%) blend, a maximum attenuation of −27 dB was obtained, which was improved by up to −51 dB (max.) by the incorporation of 5 wt% GNP, mostly due to the absorption phenomena. The dispersion state, nature of the fillers and interactions of the fillers with the polymer matrix are reported to be the main reasons for the improvement of the EMI shielding of these hybrid nanocomposites. The influence of the carbon-based fillers on the EMI shielding performance of the conductive polymers was also deeply described in many other works, as for instance.^105–108^ However, some researchers have started to focus on the investigation of untraditional natural fillers, such as halloysite, montorillonite, chitosan or cellulose, as potential additives for the improvement of the shielding effectiveness of conductive polymers. Abdi *et al.*^109^ studied the electrical conductivity and shielding effectiveness of the polypyrrole/chitosan (PPy/CHI) conductive composites. They found out that chitosan can enhance the thermal and electrical properties, and EMI SE of PPy. The calculated theoretical and experimental values demonstrated good correspondence of the shielding efficiency of the tested composite films at high frequency and conductivity. The research^110^ was focused on the preparation of PEDOT:PSS films hybridized with halloysite nanotubes (HNT), which were evaluated for EMI shielding for the first time. The research revealed that the highest EMI shielding efficiency of the hybrid film was −16.3 dB in the tested frequency range from 2 to 13 GHz for the PEDOT:PSS film hybridized with 75 wt% of HNT, using a test specimen with 4.5 mm in thickness. It was mainly a contribution of dielectric loss, rather than magnetic loss, to the overall EMI SE. The authors of the study^111^ revealed that a superior electrical conductivity of 38.5 S cm^−1^ and overall EMI SE of −30 dB (−545 dB mm^−1^) over the 0–15 GHz frequency range were achieved with a thin organic paper of (55 μm) cellulose nanofiber (CNF)/polyaniline doped with (±)-10-camphorsulfonic acid nanohybrid. Application of CNF led to good mechanical properties without lowering the electrical properties of PANI. There have also been published results of investigations of the EMI shielding effectiveness of conductive PPy/montmorillonite composites,^112^ PPy/thermoplastic polyurethane/montmorillonite composites^113^ or PPy/poly(vinylidene flouride)/montmorillonite composites.^114^ The results demonstrated that such composites are promising materials for EMI shielding applications.

In many spheres, such as radar absorbers, the EMI shield should possess electric and/or magnetic dipoles, which can interact with the orthogonally pulsating electric and magnetic fields of the impinging EM radiation. Application of various inorganic dielectric or magnetic fillers with high permittivity or permeability (mainly ferrites or titanates, as for example, Fe_3_O_4_, BaFe_12_O_19_, and BaTiO_3_) can provide a unique combination of properties, like moderate electrical conductivity and good magnetic/dielectric properties. The shielding response can thus be reached by the balanced combination of magnetic, dielectric and ohmic losses.^115,116^ The electric field losses are caused by the dielectric relaxation effect connected with induced and permanent molecular dipoles. As the frequency of the incident EM wave increases (especially in the microwave region), dipoles present in the system fail to maintain in-phase movement with a rapidly pulsating electric vector. Such out-of phase movement of dipoles leads to molecular friction, resulting in energy dissipation in the form of heat. By contrast, magnetic losses are related to the permeability of the material and occur due to phenomena such as domain wall movement, hysteresis, eddy-currents or ferromagnetic resonance. Therefore, microwave absorption is an associated effect of magnetic and dielectric losses, along with matching thickness and finite conductivity.^42,77^ Gairola *et al.*^82^ prepared highly conductive nanocomposites by the mechanical blending of polyaniline and Mn_0.2_Ni_0.4_Zn_0.4_Fe_2_O_4_ ferrite. They investigated the complex permeability, permittivity and shielding effectiveness of the composites with different thicknesses over the X-band frequency range. The result showed that the composite with 2.5 mm in thickness showed high shielding efficiency (49.2 dB) due to absorption. PANI filled with 20 wt% of Y_2_O_3_ showed an overall EMI SE of 19–20 dB over the frequency range of 12.4–18 GHz. The anisotropy formed by the application of Y_2_O_3_ contributed to various polarization losses with a subsequent increase in the EMI shielding by absorption.^117^ In the research,^118^ polyaniline doped with *para*-toluene sulfonic acid (PANI/PTSA) and cobalt ferrite (CoFe_2_O_4_) was synthesized by sol–gel method. The composite exhibited a wider absorption frequency range and maximum reflection loss of −28.4 dB (99.8% power absorption) at 8.1 GHz and −9.6 dB (more than 90% power absorption) at 11.2 GHz. Saini *et al.*^65^ revealed that the incorporation of tetragonal BaTiO_3_ into polyaniline also led to the improvement of EMI absorption. Li *et al.*^80^ investigated PANI/MWCNT/Sr_12_O_19_ nanocomposites for electromagnetic shielding applications. The microwave absorption was outstanding within the 2–18 GHz frequency range. In the range of 2–9 GHz, it depends mainly on the dielectric loss. Finally, in the 9–18 frequency range, it mostly depends on the magnetic loss. The results demonstrated that the composites filled with ferrites and other combined metal oxide-based fillers exhibit great potential in applications, such as microwave absorbers.

### 5.3 Conductive polymer composites

With the exclusion of ICP, the majority of polymers exhibit zero or only a minimal intrinsic conductivity. In general, it can be said that polymer matrices are typical electric insulators, thus they are not able to provide shielding effects. However, when they are filled with suitable materials (mainly different types of fillers in the form of particles, fibers, flakes, ribbons, platelets, sheet or tubes), such composites take part in significant status among materials able to provide protection against EMI. Despite the fact that polymer matrices are electric insulators and do not contribute to shielding, they can influence the dispersion and distribution of fillers, and the formation of a filler network structure within the polymer matrices, and thus they enhance the shielding efficiency. Non-conductive polymers containing conductive fillers, known also as extrinsic conductive polymer composites, have been deeply investigated as potential materials for EMI shielding applications due to their lightweight property, stability, flexibility, extensive absorption, corrosion resistance, and ease of processing. They can be bent, shaped, or coiled without the loss of their electromagnetic properties. Moreover, due to the appropriate electrical and dielectric characteristics, they are able to avert electrostatic discharge, disturbance and interference between the electronic systems. Having the outlined advantages, extrinsic conductive polymer composites have been used in practical applications in the sphere of automobiles, aircraft, aerospace, civil engineering, or commonly used electronic devices. The versatility and easy adaptability to the requirements of different technological applications have ranked the conductive polymer composites among perspective alternatives to metal enclosures used in power electronics, communication systems, generators and electric motors. The easy processing, in line with the good tensile characteristics, and design flexibility provide benefits over traditional materials that are limited by intensive time and labor processes.^119^ Different fillers have been introduced into polymer matrices in order to prepare efficient EMI shielding polymer composites, such as metals, metal oxides, ferrites and other inorganic fillers, as well as various types of carbon-based structures, such as carbon black, carbon nanotubes, carbon nanofibers, graphite, or various forms of graphene. In polymer composites, the two major constituents are the matrix phase and the reinforcement filler phase. However, the interfacial region between the matrix and the filler forms the third material constituent, which significantly affects the macroscopic characteristics of the composite materials. In the interfacial region, composites exhibit substantially different properties when compared to complex composite structures.^59,68^

When conductive or semi-conductive fillers are distributed within the insulating polymer matrix, they act as “micro-capacitors”, enhancing their ability to store the electric energy. This affects the electrical properties of the composites according to the degree of filling and distance between the applied filler particles. When particles are not in contact, the conductivity of the composites only slightly changes, although the dielectric properties may change significantly. However, when the conductive fillers are close to each other, electrons can jump the gap between particles with the formation of a current flow.^13^ When the content of the conductive fillers in the polymer matrix reaches a critical concentration, an interconnected network of the filler particles is made up. This transition is known as the percolation transition, and the critical concentration of the conductive fillers at the percolation transition is termed as the percolation threshold.^38^ At the percolation threshold, a continuous electric current pathway is established. Above this concentration, the electrical conductivity of polymer composites increases significantly. In addition to the filler content, the percolation threshold is dependent on the geometrical factors of the filler, including the size, shape, morphology, orientation, aspect ratio, as well as the electrical conductivity of the filler. The influence of these factors on the electrical conductivity of the polymer composites is described in more detail in Section 2. Moreover, the percolation transition depends also on the dispersion, distribution of the filler within the polymer matrix, mutual adhesion and compatibility between the filler and the matrix, and the processing method.^48^ In general, as the dispersion state of the filler within the polymer matrix and the mutal adhesion between the filler and polymer increases, the percolation threshold decreases. It can also be stated that the high structure fillers (as for instance, high structure carbon black) can form an interconnected filler network at lower concentrations when compared to traditional spherical-type fillers.^61^

**Fig. 9:**
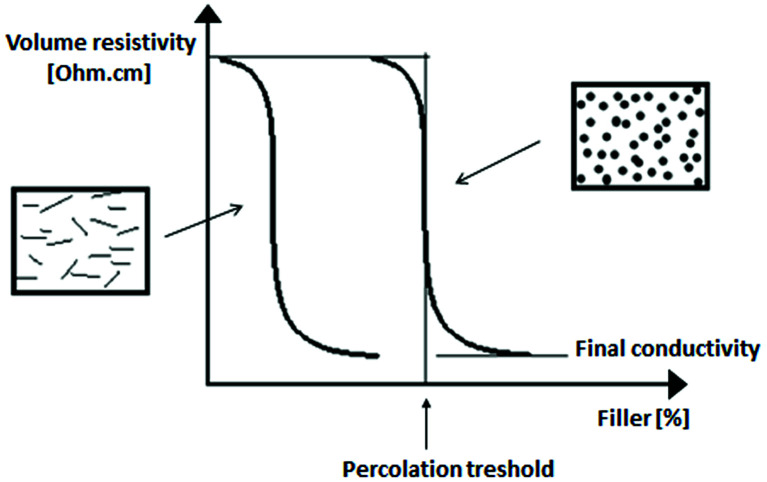
General dependence of the resistivity of composites on the conductive filler content with respect to the filler aspect ratio.

The general dependence of the conductivity of polymer composites on the conductive filler content with respect to the aspect ratio is displayed in Fig. 9. In general, for conventional roughly spherical-shaped fillers with random distribution, around 10–20% has to be added into the polymer matrices in order to reach the percolation threshold. With increasing aspect ratio (length-to-width ratio) of the particles, the concentration decreases for the percolation threshold to be achieved. In polymer composites with conductive fillers below the percolation threshold, the influence of the frequency on their dielectric constant and dielectric loss factor in the tested frequency range is fairly limited, and their values increase with the increase in filler loading. On the other hand, the values of these parameters are frequency-dependent above the percolation threshold.^120^ There are two theories or theoretical models that describe the conductive mechanism of the composites. The first one is the conductive channel theory, and the second one is the tunnel effect theory. The conductive channel mechanism can be applied at high filler content (above percolation threshold) when the particles in contact form conductive channels, and the charge carriers can freely migrate within the system. The tunnel effect theory plays a role at low filler content when the polymer matrix surrounds the filler particles, and thus there is certain spacing between the conductive particles. Electrons in the thermal vibration are under the action of migration with the formation of a conductive network.^121^

On the other hand, it must be taken into account that increasing the amount of filler can have detrimental effects on the processing characteristics, viscosity, thermo-oxidative stability and physical–mechanical properties of the composites, such as the tensile strength, tear strength, flexibility or dynamic characteristics, and heaviness. This is mainly the case of the inorganic metal fillers, metal oxides or ferrites, which are usually heavyweight and have been proved to be mostly inactive fillers from the point of the physical–mechanical properties when incorporated into the polymer matrices. Moreover, fillers based on transition metals and metal oxides (especially iron, copper, manganese) have been proved to be polymer poisoning, lowering the thermo-oxidative stability of polymer composites. On the other hand, carbon-based fillers impart not only good EMI shielding characteristics to the polymer composites, but also contribute to the improved physical–mechanical properties, like high tensile strength and moduli. Among them, carbon black is a cost-affordable and widely used reinforcing filler, mainly in rubber technology. Outlining these facts, not only the conductivity, dielectric or electromagnetic characteristics of fillers, but also their physical and structural characteristics affecting the physical–mechanical properties and stability of composites must be considered when formulating and designing the polymer composites for particular EMI shielding applications.

#### 5.3.1 Metallic fillers

Metals belong to the oldest materials used for electromagnetic interference shielding. Due to their excellent conductivity, they seem to be ideal materials for EMI shielding. However, the main mechanism of their shielding efficiency is based on the reflection of EM radiation. In many cases, the shielding by reflection is undesirable due to the secondary interference of the reflected EM waves with the initial radiation emitted from other electronic sources, causing a secondary electromagnetic radiation effect. Formerly, metals were used mainly in the form of sheets, which is impractical due to their heavy mass and complicated manipulation. Metal shields are also susceptible to corrosion, leading to the nonlinearity and decrease in the shielding effectiveness. Metals are also rigid, expensive and difficult to process in addition to their high production cost. Nowadays, lightweight aluminum sheets are partially applied, but metals are often used in the form of thin layers deposited on a suitable surface, then in the form of powdery fillers, fibers and alloys in composites with different types of matrices. They are also used in combination with other shielding materials as carbon fibers, carbon nanotubes, graphite or graphene. Metal-based shielding materials show the highest efficiency at lower frequencies of electromagnetic radiation, at which the effect of the reflection shielding is the most pronounced. With increasing frequency, the shielding effectiveness of the metal shields decreases. The experimental works revealed that the EMI SE of metals and metal materials in the frequency region between 0.1 GHz and 1.5 GHz ranges from −30 dB up to −60 dB depending on the applied form (sheet, fiber, powdery filler) and on the degree of filling. The most common metals used as EMI shields are copper, cobalt, nickel or iron. The others include aluminium, brass, silver, steel and tin. The electrical conductivity of some metals is shown in Table 2. Copper provides the highest shielding efficiency, as it is the best electrical conductor. However, it also exhibits the highest proportion of reflection shielding. On the other hand, iron shows the lowest shielding efficiency, but its adsorption mechanism was found to be dominant. Jalali *et al.*^122^ investigated the influence of cobalt, nickel, iron and iron oxide (Fe_3_O_4_) as nanoparticle fillers in epoxy thermosetting resin on the shielding effectiveness of the prepared composites over the X-band frequency range (8.2–12.4 GHz). When comparing the tested materials, composites filled with iron particles showed the most stable and predictable shielding and absorption behavior. Nickel, cobalt and iron oxide showed lower absorption shielding efficiency, but also lower reflection shielding. Composites filled with iron oxide exhibited the lowest reflection of the EM waves (∼5%), which can be attributed to the low conductivity of iron oxide (six magnitudes smaller than iron). Then, the authors tested the influence of the particle size of iron and nickel on EMI SE. It was found out that iron nanoparticles with the smallest diameter (25 nm) exhibited the highest adsorption ability from all analyzed materials. Though, again, they showed the highest reflection of the electromagnetic field power. The iron particles with diameters of 50 nm provided the lowest reflection loss, while still showing higher absorption loss than particles of 75 nm. The incorporation of iron nanoparticles into carbon fiber-reinforced polymer composites led to the increase of the total EMI SE up to 15 dB, implying a rise from 30 dB without iron nanofiller up to 45 dB with inclusion, in the considered 8.4–12.4 GHz frequency range. The improvement was moreover formed through a decrease of reflection and increase of adsorption shielding. Wu *et al.*^123^ studied the influence of the iron particle size in epoxy resin on the adsorption shielding efficiency of the prepared composites. Their research revealed that adsorption shielding significantly increased with a decrease in the particle size. The size of the particles also had considerable influence on the shielding efficiency of the tested materials – the smaller were the particles, the higher was the overall EMI SE. Highly conductive copper nanowire (CuNW)/polystyrene (PS) composites were investigated in the work.^124^ The percolation threshold of the composites was reached only at 0.24 vol% of CuNW. The analysis of the shielding effectiveness within the X-band frequency range showed that the composite film with thickness of 210 μm containing 1.3 vol% of CuNW exhibited an EMI SE value of 27 dB. When the content of the copper nanowire in PS was 2.1 vol%, the shielding efficiency increased up to 35 dB. The contribution of the absorption of both types of composites to the overall EMI SE was about 54%. Jiao and his coworkers^125^ presented an easily-operated and scalable method, which included pyrolysis and magnetron sputtering, to fabricate core–shell structured composite comprising nano-copper (nCu) and cotton-derived carbon fibers (CDCF). Outstanding hydrophobicity and excellent antibacterial activity were achieved for the composite material owing to the composition conversion from cellulose to carbon, nano-size effect and strong oxidizing ability of the oxygen-reactive radical sites from the interactions of nCu with the sulfhydryl groups in the enzymes. In addition, the core–shell composite showing strong electrical conductivity induced the conduction loss and interfacial polarization loss, which resulted in a high absorption-dominated EMI SE of 29.3 dB within the X-band frequency range. Yu *et al.*^126^ prepared high electrical conductive films by the blending of silver nanowires (AgNW) and silver nanoparticles (AgNP) with hydrophobic and hydrophilic resins. The silver content in the tested films filled with AgNW could be significantly reduced due to the high aspect ratio of the silver nanowires. The overall EMI SE of the composites increased with increasing content of silver. When the shielding efficiency was under −20 dB (>99%) in the tested frequency range of 3–17 GHz, the AgNW-filled film exhibited higher EMI SE than that filled with AgNP, which can be attributed to fewer requirements in the content. Highly flexible ultralight biopolymer aerogels, consisting of cellular biomimetic microstructures formed from silver nanowires (AgNW) and cellulose nanofibers (CNF), were fabricated *via* the facile and convenient freeze-casting method.^127^ The honeycomb-like, lamellar and random porous scaffolds were obtained by adjusting the freezing techniques to modulate the relationships among the microstructure, macroscopic, mechanical and EMI shielding performance. The lamellar, porous biopolymer aerogels exhibited impressive EMI SE exceeding 70 or 40 dB over the X-band frequency range and normalized specific surface EMI SE of up to 178 235 dB cm^2^ g^−1^. The prepared CNF/AgNW materials exhibited hydrophobicity and antibacterial properties, which extended the application potential of the hybrid biopolymer aerogels. Although there have been several polymer composites with nanostructured silvers exhibiting moderate stretchability, their EMI SE usually deteriorates consistently with stretching. Therefore, Yao *et al.*^128^ developed a highly stretchable polymer composite based on a three-dimensional (3D) liquid-metal (LM) network embedded in polymer matrix, which exhibited significant improvement in EMI SE when stretched. The composite matches the EMI SE of metallic plates within the unexpectedly broad 2.65–40 GHz frequency range. The electrical conductivity of the 3D LM composite is among the state-of-the-art in stretchable conductors under high mechanical deformations. With skin-like elasticity and toughness, the composite material paves the way for emerging soft and human-friendly electronics. Composites having conductive coatings are other common forms of composite shielding materials. The surface-type conductive polymers can be prepared by different methods, such as electroplating, electroless plating, ion plating, vacuum deposition, zinc flame spraying, zinc arc spraying, conductive paints, cathode sputtering, foils, laminates, and tapes.^27,121^ However, the outlined operations for surface treatment are secondary techniques to the mixing and molding procedures, and require additional surface preparation, as well as special construction equipment. Low mechanical properties, high rigidity, poor wear and scratch resistance are the next drawbacks, which limit the application of metalized polymers, such as the shielding materials in many applications.^129^ Also, as metals are prone to corrosion, this effect may lead to the non-linearity in surface compactness, and thus negatively influence the shielding performance. Shipeng *et al.*^130^ examined the effect of various metal fillers on the shielding efficiency of electromagnetic shielding coatings. They revealed differences in the microstructure of coatings prepared with different morphologies of metal powders. Silver powder and the silver-plated copper powder are flakes in the material preparation process. When deposited on the surface, the metals overlap the surface very well. This leads to the reduction of voids on the surface layer, leading to the improvement of high-frequency shielding performance. Nickel powder is spherical with more hollows in the microstructure. As the EM wave frequency increases, the electromagnetic radiation on the coating penetration capacity increases, resulting in lowered EMI shielding ability. The coating of metallic nickel on the activated carbon fiber felt (ACFF) *via* the electrodeposition method and investigation of EMI SE of the prepared materials in the X-band frequency was the main goal of the work.^12^ Ni coating on ACFF lowered the absorbed process, and the main mechanism of EMI shielding was based on reflection (almost 94% at around 8 GHz). ACFF in the felt form contributed to the decrease of the EM wave transmission, while the nickel-coated film contributed to the increase of the EM wave reflection. Yoo-Ji Yim *et al.*^131^ used the electroless plating method for the fabrication of nickel/copper sulfide–polyacrylonitrile (Ni/CuS–PAN) fibers, and investigated their EMI shielding properties. The prolongation in the nickel-plating time led to the increase of EMI SE. The highest overall shielding efficiency was reached at 50 min electro-deposition at 2.05 GHz with 45 dB (99%) of shielding. Electroless plating was also used for the deposition of Ni/Co on the web-like biocarbon nanofibers.^132^ Biocarbon nanofibers (CF) with a novel web-like structure, consisting of interconnected and entangled carbon nanoribbons, were obtained from the pyrolysis of bacterial cellulose at 1200 °C. Paraffin wax used as a matrix was filled with different amounts of CF and Ni–Co coated CF (NCCF). The results revealed that EMI SE of NCCF composites were found to be significantly higher in comparison with those filled with CF at the same loadings. The composites containing 30 wt% of NCCF showed the highest overall EMI SE of 41.2 dB (99.99% attenuation), which can be attributed to the higher permittivity and electrical conductivity of NCCF composites. Hu *et al.*^133^ developed a high-yield synthesis strategy for silver nanowires by an injection polyol method. Then, they prepared sandwich-structured flexible films with poly(ethersulfones) and polyethylene terephthalate, which exhibited outstanding EMI SE. Ma *et al.*^134^ prepared a novel ferro-aluminium-based sandwich composite for EMI shielding by hot pressing, followed by diffusion treatment. The results showed that the shielding efficiency of the sandwich composite reached 70–80 dB over the frequency range 30 KHz to 1.5 GHz, and was higher when compared to that of pure iron plate. The multiple reflection loss was reported to be the primary contribution to the overall EMI SE of the sandwich composite.

**Table 2:** Electrical conductivity of metals

Metals	Conductivity (S m^−1^)
Silver	6.3 × 10^7^
Copper	5.9 × 10^7^
Gold	4.5 × 10^7^
Aluminium	3.8 × 10^7^
Cobalt	1.6 × 10^7^
Brass	1.6 × 10^7^
Nickel	1.4 × 10^7^
Iron	1.0 × 10^7^
Tin	8.7 × 10^6^
Steel	6.2 × 10^6^
Lead	4.8 × 10^6^

#### 5.3.2 Magnetic fillers

In contrast to the highly conductive metals with mostly reflection-dominated shielding mechanism, materials possessing electric and/or magnetic dipoles are suitable for shielding applications, in which the prevailing shielding mechanism is absorption. Electric dipoles exhibit materials with high permittivity, such as Fe_2_O_3_, ZrO_2_, SiO_2_, TiO_2_, MnO_2_, ZnO or BaTiO_3_, whereas magnetic dipoles are present in materials with high permeability (*e.g.*, Fe_3_O_4_, different types of ferrites, mumetal, super permalloy). The high initial permeability of magnetic fillers, for instance ferrites, gives the possibility for the fabrication of polymer composites with absorption-dominated shielding at the broadband frequency range. One of the biggest disadvantages of magnetic fillers is their worse dispersion within polymer matrices, and the fact that magnetic fillers are inactive fillers from the point of their physical–mechanical properties. This means that their incorporation into polymer matrices is usually connected with the deterioration of the tensile strength characteristics of composite materials. The problem can be solved by the combination of magnetic fillers with carbon-based fillers, which facilitates the dispersion of magnetic fillers within the polymer matrices by increasing the viscosity of the polymer melts, and thus increasing the shear stress during compounding on the one hand. On the other hand, carbon-based fillers as reinforcing fillers contribute to the improved physical–mechanical properties of polymer composites. The dispersion of magnetic fillers can also be improved by using the segregated microstructure techniques, followed by the enhanced conductivity characteristics of composites (see Section 5.3.5.2). The combination of magnetic fillers and carbon-based fillers, or the preparation of composites by the segregated microstructure approach, seems to be the future trend in the development of polymer composites with incorporated magnetic fillers.

In the work,^135^ composites based on various transition metal oxides as iron oxide (Fe_2_O_3_), silicon dioxide (SiO_2_), zinc oxide (ZnO), zirconium dioxide (ZrO_2_), and titanium dioxide (TiO_2_) in the polyvinyl alcohol matrix were studied as candidates for EMI shielding materials in the C-band frequency range (4–8 GHz) and X-band frequency range (8–12 GHz). The amount of metal oxides in the composites, which were prepared by solvent casting method, ranged from 0.1 to 10 wt%. At 10 wt% of transition metal oxides in PVA, the minimum reflectivity of the composites was found to be −38.85 dB (10.4 GHz for Fe_2_O_3_), −41.90 dB (10.4 GHz for SiO_2_), −33.65 dB (10.4 GHz for ZnO), −24.90 dB (11.0 GHz for ZrO_2_), and −32.90 dB (9.76 GHz for TiO_2_). The authors revealed that the losses in the PVA composites based on SiO_2_, ZnO, ZrO_2_, and TiO_2_ were dielectric in nature, whereas the PVA/Fe_2_O_3_ composite exhibited both dielectric and magnetic losses. The results also demonstrated that composites based on Fe_2_O_3_ and SiO_2_ exhibited good mechanical strength and high thermal stability, and were reported to be low-cost, efficient EMI shielding materials. Ahmad *et al.*^136^ prepared composites based on polycaprolactone (PCL) and nickel oxide (NiO) using the melt blend technique, and investigated their dielectric properties and shielding effectiveness in the 8–12 GHz frequency range. The results showed that the permittivity of the tested composites showed a decreasing trend with an increase in frequency, but increased with increasing content of NiO. This can be attributed to the interfacial polarization on the boundaries between the two phases, where the space charges of NiO accumulate at the interface. The absorption increment was found to be higher than the reflection increment due to the interaction of the EM waves with electric dipoles. The increase of the absorption shielding was recorded with increasing content of NiO due to interfacial polarization, which increased the absorption increment (from 7.1 dB at 5 vol% to 11.4 dB at 25 vol% loading). The reflection increment also increased with increasing content of NiO as a result of the conductivity increase (from 0.33 dB at 5 vol% to 1.04 dB at 25 vol% loading). As a consequence, the overall EMI SE was also enhanced with increasing content of nickel oxide. The authors of work^137^ investigated the shielding effectiveness of composites based on poly(methyl methacrylate) and silver nanowire/manganese dioxide nanowire (PMMA/AgNW/MnO_2_NW), which were fabricated by solution casting method. The prepared nanocomposites exhibited low dielectric loss (0.31 at 8.2 GHz) and excellent dielectric permittivity (64 at 8.2 GHz), which were reported to be among the best values published, over the X-band frequency range. The outstanding dielectric properties of the composites can be attributed to the dimensionality match between the fillers, higher dispersion state of the fillers, positioning of the ferroelectric MnO_2_NW in-between space of AgNW, which increased the dielectric permittivity of the composites. Furthermore, MnO_2_NW acted as a barrier, which cut off the contact spots of AgNW, resulting in lower dielectric loss. The aligned structure of AgNW increased the specific surface area of AgNW. Bayat *et al.*^138^ examined carbon nanofiber-based composites filled with iron oxide nanoparticles with two different size regimes, ranging from 10–20 nm (superparamagnetic) to 20–30 nm (ferromagnetic). The composites were synthetized using the electrospinning process, followed by heat treatment. The results showed that a higher degree of graphitization, magnetic strength and electrical conductivity were obtained for composites comprising larger iron oxide nanoparticles (20–30 nm). During composite processing, a transition from superparamagnetic to ferromagnetic characteristics was observed. An EMI SE of 68 dB (at frequency 10.4 GHz) was recorded for the composite filled with larger iron oxide nanoparticles carbonized at 900 °C.

Spinel ferrites with different compositions (for instance, Fe_3_O_4_, NiFe_2_O_4_, CoFe_2_O_4_, Mn_1−*x*_Zn_*x*_Fe_2_O_4_ and others) have been used as the most common additives in polymer matrices due to their simple chemical composition and efficient preparation.^139–143^ Li *et al.*^144^ used the ceramic method for the preparation of spinel ferrite with composition Ni_0.32_Cu_0.08_Zn_0.6_Fe_2_O_4_. Ferrite was ball-milled for 2 h, 4 h, 6 h, 8 h, 10 h, 12 h, and 14 h, followed by subsequent drying for 24 h. The complex permeability, complex permittivity and EMI shielding characteristics of the ferrite–polymer composites prepared with an 80 wt% ferrite ratio in the paraffin matrix were investigated in the frequency range of 1 MHz to 1 GHz. The complex permittivity and permeability of the composites were found to decrease with an increase in the ferrite ball milling time. The EMI SE of the composites also showed a decreasing trend with an increase in the ferrite ball milling time, and increased with increasing frequency. The maximum EMI SE value of 3.75 dB was reached at 1 GHz and 10 mm thickness of the composite. The results revealed that the reflection loss and multiple reflection loss were much lower than the absorption loss in the tested frequency range, which indicates that absorption is the dominant mechanism of shielding. Shannigrahi *et al.*^145^ synthetized single phase ferrite powders with the general composition (Ni_*x*_R_1−*x*_)_0.5_Zn_0.5_Fe_2_O_4_, in which R = Mn, Co, Cu, *x* = 0, 0.5 using microwave sintering at 930 °C for 10 min in air atmosphere, and incorporated them into the polymethyl-methacrylate matrix. The composite materials were prepared by melt bending and were studied for their microstructure, thermal and magnetic characteristics, as well as 3D magnetic field space mappings. Among the tested ferrites, cobalt-doped ferrites and their composites exhibited the best EMI shielding effectiveness, and are of great interest as candidates for EMI shielding applications. Jagatheesan *et al.*^146^ investigated the electromagnetic absorption characteristics of the carbon helical yarn fabric-based composites and manganese–zinc (MnZn) ferrite filler-loaded 3 phase fabric composites. The carbon helical yarn fabric composite was fabricated by sandwiching a fabric with polypropylene (PP) films and thermally pressed. The results revealed that the absorption loss of the composites was low in the C-band frequency region (4–8 GHz). To enhance the absorption coefficients, MnZn ferrite particles were dispersed in the PP matrix. The PP/ferrite composites showed higher permeability and permittivity values. Thus, the absorption loss of the composite materials was improved. As the filler content increased, the magnetic permeability and dielectric loss of the PP/ferrite composites increased due to the enhanced magnetic loss. The increasing content of ferrite resulted in the improvement of the absorption loss and total EMI SE. The work^147^ deals with the investigation of the shielding characteristics of the three-phase percolation composites, which were prepared by dry-mixing technique from Ni fillers, microsized NiCuZn ferrites and polymer epoxy matrix. The results demonstrated that Ni fillers occupy the inside of the polymer gap, and act as magnetic bridges to improve the magnetic exchange coupling and effective permeability due to the decreasing demagnetization field. The frequency response of the microwave absorption in composites is attributed to the dielectric and magnetic losses correlated with the spin resonance, domain-wall motion and dipolar relaxation. The increasing content of Ni in the composites caused a significant increase in their microwave absorbing properties. Below 0.7 GHz, the microwave absorption of composites occurred mainly due to dielectric loss. On the other hand, the magnetic loss mainly contributed to the absorption shielding at higher frequency. The authors also stated that the double-layer absorbers, which are laminated with high dielectric/magnetic loss structure, exhibit a higher return loss, lower matching frequency and narrower absorption bandwidth. Sanidi *et al.*^148^ investigated the thermomechanical, magnetic and dielectric properties of the composites consisting of ceramic zinc ferrite nanoparticles with the structural formula ZnFe_2_O_4_ incorporated in the thermoset epoxy resin. The presence of the nanoparticles resulted in the enhancement of the thermomechanical, as well as dielectric response. Three relaxations were observed in the dielectric spectra of the composite systems, originating from the polymer matrix and the filler. These are the interfacial polarization, which is due to the accumulation of free charges at the filler–rubber interface, the glass to rubber transition of the polymer matrix, and the rearrangement of the small polar side groups of the polymer chains. Zinc ferrite imparts magnetic properties to the polymer matrix. As a result, the magnetization of the composites increases with increasing filler content. The results also revealed that the composites exhibit superparamagnetic behavior at room temperature. Sýkora *et al.*^63^ investigated and compared the absorption shielding efficiency of the composites based on acrylonitrile–butadiene rubber (NBR) filled either with magnetic soft lithium ferrite (Li) or manganese-zinc ferrite (MnZn). The results revealed that composites containing 27 vol% of the fillers exhibited sufficient absorption shielding ability. The absorption maxima of the composites were reached at −30 dB at 1.1 GHz frequency and −35 dB at 0.9 GHz frequency for NBR/Li ferrite composites or NBR/MnZn ferrite composites, respectively. Composites filled with Li were reported to be more suitable for absorption shielding than those of NBR/MnZn due to their lower optimal material thickness and wider absorbing frequency bandwidth. Dosoudil *et al.*^149^ investigated the influence of the particle size, content, and fraction ratio of the dual MnZn/NiZn ferrite filler on the complex permeability and EM-wave absorbing properties of composites based on polyvinylchloride (PVC) within the frequency range of 1–3000 MHz. The commercially available ferrites with structural formula Mn_0.52_Zn_0.43_Fe_2.05_O_4_ and Ni_0.33_Zn_0.67_Fe_2_O_4_ were fabricated by using a ceramic route at 1200 °C for 4 h. The relaxation type frequency dispersion of permeability was caused by the resonance of the vibrating domain walls and the ferromagnetic natural resonance of the magnetic moments in the domains. The results also revealed that in the designed single layer PVC absorbers, the absorbing characteristics, such as the return loss, matching thickness, matching frequency, bandwidth for return loss ≤−20 dB, and the minimum value of return loss were found to be strongly dependent on the concentration, particle size and fraction ratio of the dual MnZn/NiZn ferrite filler. The same group of authors also examined the high-frequency EM-wave absorbing characteristics of the metal alloy/spinel ferrite/polymer composite materials (FeSi/NiZn/PVC) with various FeSi particle contents.^150^ The results showed that increasing the amount of metal alloy in the composites resulted in smaller matching thickness and higher matching frequency than that of the NiZn/PVC composite. The return loss calculations revealed that the tested composites are good EM-wave absorbers in the quasi-microwave band (100 MHz to 3 GHz), and thus suitable for EMI noise suppression in the electronic communication systems.

In addition to the spinel ferrites, hexagonal ferrites (including M-type, X-type, Y-type, U-type, Z-type or W-type) are also of significant interest as high-frequency microwave-absorbing materials because of their planar magnetic anisotropy and natural resonance in the gigahertz range.^70,151^ M-type strontium ferrites (SrFe_12_O_19_) and barium ferrites (BaFe_12_O_19_) are typical examples of the hexagonal group that exhibit significant uniaxial anisotropy, strong permeability and high saturation magnetization.^152–154^ Thus, the incorporation of those ferrites into polymer matrices has taken a considerable share in the field of high-performance microwave absorbers over the last decades.^155–157^ Tabatabaie *et al.*^158^ incorporated the M-type strontium hexaferrite SrFe_9_Mn_1.5_Ti_1.5_O_19_ into the PVC matrix at concentrations of 50 wt%, 60 wt%, 70 wt% and 80 wt%. The microwave absorption characteristics of the composites were evaluated over the 12–20 GHz frequency range. The results demonstrated that the filler with wide particle size distribution formed a magnetoplumbite structure inside the polymer matrix. The composite filled with 70 wt% ferrite concentration and having a thickness of 1.8 mm exhibited a reflection loss below −15 dB in the 16.4–19.4 GHz frequency range. This composite also showed good absorption performance within the 8–18 GHz frequency range. Bao-Wen *et al.*^159^ investigated three-phase composites based on Ni/hexagonal-ferrite (Co2Z or Zn2Y barium ferrites)/polymer for EMI shielding. In comparison with the Ni/polymer composites or hexagonal ferrite/polymer composites, those materials showed excellent microwave absorption properties within the X-band frequency range, and were strongly dependent on the combination of the three phases. The high EMI SE of the composites and much larger contribution of absorption to overall shielding effectiveness suggest that such composites could be promising for applications as thinner and lighter EMI absorbing materials.

Titanites are also very promising materials for the construction of EMI shielding polymer composites, mainly due to their high values of dielectric constant (permittivity). Chauchan *et al.*^160^ investigated high-performance barium titanate (BaTiO_3_) filled poly(ether ketone) (PEK) composites for their thermal, thermomechanical and dielectric properties, as well as EMI shielding ability. The results showed that the dielectric constant of the composites increased roughly three times by the incorporation of 18 vol% BaTiO_3_ in the frequency range of 8.2–12.4 GHz. This increment in dielectric constant was reflected in the enhanced EMI shielding properties. The overall EMI SE of −11 dB (∼92% attenuation) at 18 vol% of BaTiO_3_ justifies the use of those composites for the suppression of EM waves. Joseph *et al.*^161^ examined the EMI shielding effectiveness of the composites based on polyvinylidene fluoride filled with micro- and nano-sized BaTiO_3_ powders. The results revealed that the PVDF composites filled with nano-sized filler exhibited better shielding characteristics when compared to those filled with micro-sized BaTiO_3_. The highest overall EMI SE of 9 dB within the X-band frequency range was found to have a composite filled with 40 vol% of nano-sized BaTiO_3_. The results also revealed that the addition of a small amount of silver particles resulted in the increase of the composite conductivity, which was subsequently reflected in the improved shielding performance. The preparation and evaluation of the EMI shielding characteristics of the composites based on the epoxy resin and nanocrystalline strontium titanate (SrTiO_3_) was the main goal of the work.^162^ The investigation of the microwave-absorbing properties of composites over the X- and Ku-bands (8–18 GHz) revealed that the composite with a higher percolation threshold provided up to 48 dB absorption. Moreover, it exhibited a wideband response of 7.1 GHz out of the total band of 10 GHz below −10 dB or less.

#### 5.3.3 MXenes

**Fig. 10:**
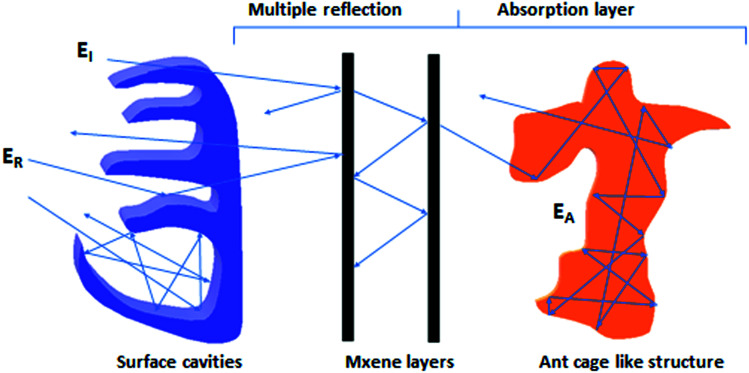
EMI shielding mechanisms of the MXene/PAT-conductive polymer composite (modified from ref. 166 with permission from the Royal Society of Chemistry, copyright 2020).

MXenes are a new family of 2D materials consisting of transition metal carbides, carbonitrides and nitrides. The general formula of MXenes is M_*n*+1_X_*n*_T_*x*_, in which M represents a transition metal element, X is carbon or nitrogen, *n* = 1–3, and T_*x*_ stands for a surface-terminating functional group, such as –OH, –F, –Cl or –O. Those surface functional moieties are directly attached to the M.^163^ MXenes are synthesized by the selective etching of certain atomic layers of sp elements from their corresponding 3D layered precursors, known as the MAX phase. MAX phases are a large family of hexagonal layered ternary transition metal carbides, carbonitrides and nitrides with the general composition of M_*n*+1_AX_*n*_, where M stands for the d-block early transition metals (Ti, Ta, Mo, Zr, Sr, V, Cr, Nb or Hf), A represents the main-group sp elements (mostly IIIA or IVA, as Al, Si, Sn, In), X stands for C and/or N, and *n* = 1, 2, or 3. MAX phases can be described as the inter-growth structures with closely packed A planar atomic layers and alternative stacking of hexagonal MX layers. In these structures, the M–X bond possesses mainly covalent/ionic characteristics, whereas the M–A bond is of a metallic nature. As the M–A bond is weaker than the M–X bond, the A layer can be easily extracted from the layered solid. Due to the different strengths and characteristics between the M–A and M–X bonds, the relatively high reactive A layer can be selectively etched by using suitable chemicals.^164,165^ Since the development of this unique class of materials in 2011, MXenes have been the subject of comprehensive ongoing research, and various strategies to fabricate good quality MXene have been developed. The A layer of the MAX phase is eliminated during the etching process, and surface functionalities are introduced. During exfoliation, the single MXene layer is separated by various processes, such as sonication and intercalation. The sonication is the physical-based method, during which MXene is ultrasonicated in water, while various organic molecules are introduced during intercalation, followed by sonication.^163^ Raagulan *et al.*^166^ developed a method for the massive production of exfoliated MXenes and their byproducts. As the interactions between the layers of MXenes are mainly physical hydrogen bonds and van der Waals bonds, water, cations and organic molecules (such as dimethyl sulfoxide or tetrabutylammonium hydroxide) can be intercalated between the interlayer spacing of MXenes. By subsequent sonication treatment, it is then possible to delaminate MXenes to form single flake suspensions.^165^ MXenes with versatile chemistry properties and 2D lamellar structures exhibit surface hydrophilic properties, high thermal and electrical conductivity, high modulus and strength, excellent volumetric capacitance, low coefficient of friction, non-susceptibility to thermal shock, good oxidation resistance and thermal stability.^167^ In addition, they can be implemented by many different cations in-between the layers. These exceptional properties provide a variety of application fields, such as catalysis, energy storage, hydrogen storage, electronics, optoelectronics, adsorption or sensors. Nevertheless, MXenes have also become highly desirable for EMI shielding applications due to their easy processing, good flexibility and high conductivity with minimal thickness.^168–170^ Sun and his co-workers in their work^171^ demonstrated an electrostatic assembly technique for the preparation of highly conductive polystyrene/MXene nanocomposites. These composites were fabricated by electrostatic assembling of negative MXene nanoplatelets on positive PS microspheres, followed by compression molding. The results showed that the nanocomposites exhibited a low percolation threshold (0.26 vol%), outstanding electrical conductivity (1081 S m^−1^) and excellent EMI shielding characteristics (>54 dB within the whole X-band frequency range). The maximum EMI SE of 62 dB at the MXene loading of 1.9 vol% was reported to be among the best performances for electrically conducting polymer composites by far. The authors stated that the work provided a new methodology to fabricate highly conducting polymer nanocomposites for efficient EMI shielding applications. Li *et al.*^172^ used a simple chemical etching process to synthesize conductive Ti_2_CT_*x*_ MXene from the precursor Ti_2_AlC MAX. The prepared MXene with a sandwich-like structure exhibited a high electrical conductivity of 0.3 S cm^−1^ and a high amount of surface functional groups. The Ti_2_CT_*x*_ MXene mixed with paraffin wax exhibited outstanding EMI shielding performance, which exceeded 70 dB in the X-band frequency range with a composite thickness of only 0.8 mm. The ordered multilayered sandwich-like porous structure enhanced the electromagnetic attenuation and multiple reflection, and thus improved the absorption shielding efficiency. In addition, the unique structure, low density, high conductivity and high amount of functional groups make Ti_2_CT_*x*_ MXene a promising candidate for energy storage and multifunctional composite applications beyond EMI shielding. In the study,^173^ MXenes with hydrophilic surfaces and unique metallic conductivity were uniformly attached to the textile fabrics *via* spray-drying coating method. By tuning the spray-drying cycles, the conductive interconnectivity for the fabrics was precisely adjusted, thus affording highly electrical conductive and breathable textile fabrics with integrated Joule heating, strain sensing and EMI shielding performances. The promising electrical conductivity (5 Ω sq^−1^ for 6 wt% or 0.78 mg cm^−2^ of MXenes decorated fabrics) further endowed the tested materials with improved Joule heating properties with a heating temperature up to 150 °C at the voltage of 6 V, strongly sensitive strain responses to human movement and excellent EMI shielding characteristics. The work introduced a new modern strategy for the versatile design of textile-based multifunctional wearable materials. Porous poly(vinyl alcohol)/MXene composite foams built by few-layered Ti_2_CT_*x*_ MXene and PVA were fabricated *via* freeze-drying method.^174^ The composite foam containing only 0.15 vol% of MXene exhibited a specific EMI SE of 5136 dB cm^2^ g^−1^ with a reflection efficiency of less than 2 dB. The perfect absorption-dominated shielding performance resulted from good impedance matching (derived from multiple highly porous structures), polarization effects (interfacial and dipole polarization) and internal reflections. Jin *et al.*^175^ prepared poly(vinyl alcohol)/transition metal carbide multilayered structured composite films *via* a multilayered casting. The PVA/MXene film containing 19.5 wt% of MXene with a thickness of only 27 μm showed an electrical conductivity of 716 S m^−1^, maximum shielding efficiency of 44.4 dB, and specific EMI SE of 9343 dB cm^2^ g^−1^. The in-plane thermal conductivity for the multilayered film was recorded to be 4.57 W m^−1^ K^−1^, which was almost 23-fold higher when compared to that of neat PVA. In addition, the multilayered architecture imparted the composite film with a remarkable anti-dripping performance. The study provided a modern and feasible approach for the manufacturing of flame-retardant, thermal conductive and EMI shielding polymer composite films. In the scientific study performed by Xie and co-workers,^176^ one-dimensional aramid nanofibers (ANF) were designed as the intermolecular cross-linker between d-Ti_3_C_2_T_*x*_ MXene flakes and ANF/d-Ti_3_C_2_T_*x*_ composite film with a multi-layered architecture, which was fabricated through a vacuum-assisted filtration method. It was found that ANF and MXene showed suitable combination by hydrogen bonding, and the ANF/MXene film displayed high electrical conductivity and outstanding mechanical properties. The ANF/MXene composite paper with an ultra-thin thickness of 17 μm exhibited impressive EMI SE of 28 dB over the 8.2–12.4 GHz frequency range, and showed promising application potential in advanced composites for sensitive electronic products. Raagulan *et al.*^166^ fabricated the Ti_3_C_2_T_*x*_ MXene/PAT–poly(*p*-aminophenol)/polyaniline co-polymer composite by a cost-effective spray coating approach. The new method was introduced to synthesize exfoliated MXene. The prepared composite exhibited good electrical conductivity of 7.813 S cm^−1^ and excellent thermal properties, such as the thermal conductivity (0.687 W m^−1^ K^−1^), heat capacity (2.247 J g^−1^ K^−1^), thermal effusivity (1.330 Ws^1/2^ m^−2^ K^−1^) and thermal diffusivity (0.282 mm^2^ s^−1^). The EMI SE of the composite was recorded to be 45.18 dB at 8.2 GHz with 99.99% shielding efficiency. The reduced form of MXene (r-Ti_3_C_2_T_*x*_) increased EMI SE by 7.26%, and the absorption shielding was significantly improved by the ant cage-like structure (Fig. 10).

#### 5.3.4 Carbon based fillers

Carbon based fillers show unique characteristics, such as high permittivity, excellent conductivity, low density, high mechanical, chemical and thermal stability, as well as outstanding physical properties. Those attributes make them suitable candidates for EMI shielding applications. Moreover, due their diverse structures, they exhibit higher or lower reinforcing character, and contribute not only to the enhanced EMI shielding characteristics of polymer composites, but also to improve their physical–mechanical and dynamic properties. These materials thus offer a great opportunity to manufacture many varieties of polymer materials with tunable electrical, optical, magnetic and physical–mechanical properties.

##### 5.3.4.1 Carbon black (CB)

**Fig. 11:**
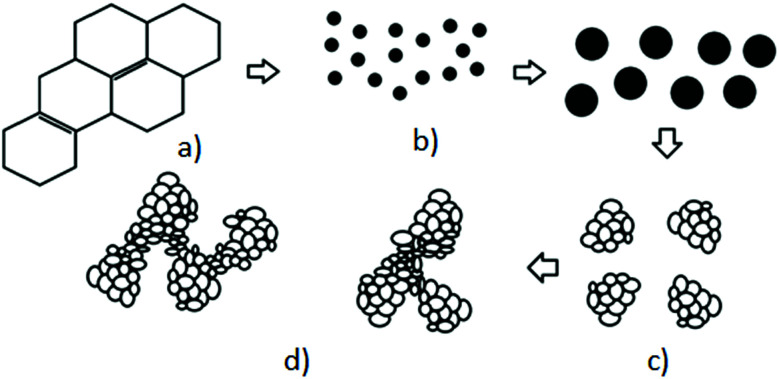
Structure of carbon black: graphitic layer (a), primary particles (b), aggregates (c), agglomerates (d).

Carbon black (CB) is the most frequently used carbon-based filler in polymer industry, especially in rubber products manufacturing due to their affordable price, ease of production and tunable physical properties. Moreover, it is an excellent UV absorber, protecting polymer composites from UV-light exposure. CB is used as a reinforcing filler in a variety of products, such as tyres, conveyor belts, mechanical goods, cables, wires, footwear or shock absorbers. Carbon black is produced by the incomplete combustion of gaseous and liquid hydrocarbons, derived mostly from petroleum. Although carbon black is mainly composed of carbon, it also contains small amounts of chemically combined hydrogen and oxygen groups. Carbon black primary particles have an onion-like paracrystalline structure (Fig. 11b). The bent and fractured crystallites consist of parallel, graphitic layer planes (Fig. 11a). The primary particles connect together to form an aggregated chain-like structure. The primary structure is then the single aggregate (Fig. 11c). A secondary structure is the agglomerate, which is formed from clusters of physically bound and entangled aggregates (Fig. 11d). The particular structure of CB depends mainly on the manufacturing and processing conditions. In general, smaller particles of CB together with higher structure and low volatility (fewer chemisorbed oxygen groups) shift towards higher conductivity in polymer composites.^177^ As the reinforcement filler, conductive CB helps to improve the conductivity of polymer composites even at lower contents in comparison to the conventional CB. Rahaman *et al.*^178^ studied the influence of two types of conductive carbon black (Printex XE2 and Conductex) on the EMI shielding characteristics of composites based on ethylene–vinyl acetate copolymer (EVA), acrylonitrile–butadiene rubber (NBR) and their blends. The results showed that Printex carbon black exhibited higher conductivity and higher EMI SE in comparison with Conductex at the same loading, which can be attributed to the higher structure (aggregating tendency, and thus formation of conductive network in polymer matrices) of Printex carbon black when compared to that of Conductex carbon black. Mondal *et al.*^179^ used a single-step solution mixing to prepare composites based on chlorinated polyethylene (CPE) and conductive Ketjen carbon black (K-CB) with high EMI shielding characteristics associated with absorption-dominant ability by conductive dissipation, as well as reflection of EM waves. The relatively low content of K-CB with respect to other conventional carbon black fillers contributed to a low percolation threshold (9.6 wt% of K-CB) and high EMI SE of 38.4 dB (at 30 wt% loading) in the X-band frequency region. Classical percolation theory demonstrated that the electrical conductivity behavior through the CPE/K-CB composite materials is quasi-two dimensional in nature. The investigation of the DC conductivity, dielectric and microwave absorption properties, and overall EMI SE of silicone rubber filled with 1–15 wt% of carbon black was the main aim of the study.^180^ The results showed that the percolation threshold was reached even at a lower amount of carbon black in the rubber matrix compared to that in the previous case (3 wt%). The complex permittivity values revealed that composites filled with 5 wt% of CB showed more than 90% microwave absorption (reflection loss ≥10 dB) within the 8–18 GHz frequency range and composite thickness of 1.9–2.7 mm. Composites filled with 15 wt% of CB exhibited −40 dB EMI SE in the tested frequency range and thickness of 2.8 mm. The authors stated that the prepared rubber composites can be used as microwave absorbers in stealth applications, as well as for EMI shielding of electronic devices in diverse civilian and military areas. Al-Saleh *et al.*^181^ investigated EMI SE and shielding mechanisms of high structure carbon black (HS-CB)/polypropylene composites in the X-band frequency range. The results revealed that composite filled with 10 vol% of HS-CB with 2.8 mm thickness showed EMI SE of 43 dB. Regardless of the composite conductivity and thickness, absorption was found to be the dominant shielding mechanism, contributing up to 87% of the total EMI SE. In the study,^182^ silicone rubber (Q)/polyolefin elastomer (POE) blends containing ionic liquids (1-vinyl-3-ethylimidazolium bromide IL) modified with carbon black (CB–IL) and multi-walled carbon nanotubes (MWCNT–IL) were fabricated by melt-blending and hot pressing. The results showed that Q/POE/CB–IL and Q/POE/CB–MWCNT–IL composites exhibited co-continuous structural morphologies. The cation π-interactions between IL and MWCNT were stronger than those between IL and CB due to the high length and large surface area of MWCNT, which facilitated better dispersion of the carbon-based fillers. The conductive networks play an important role in the reflection, absorption and scattering multiplication of the EM waves. The prepared composites Q/POE/CB–MWCNT–IL showed outstanding EMI shielding characteristics due to the synergistic effect of IL-modified CB and MWCNT. The composite with composition 60/40/15–5–4 wt% of Q/POE/CB–MWCNT–IL exhibited an overall EMI SE of 36.5 dB at 9 GHz. As carbon black is widely used filler in polymer industry, the investigation of EMI SE of the corresponding polymer composites was the subject of many other scientific works, for example.^183–186^

##### 5.3.4.2 Carbon fibers (CF), carbon nanofibers (CNF)

**Fig. 12:**
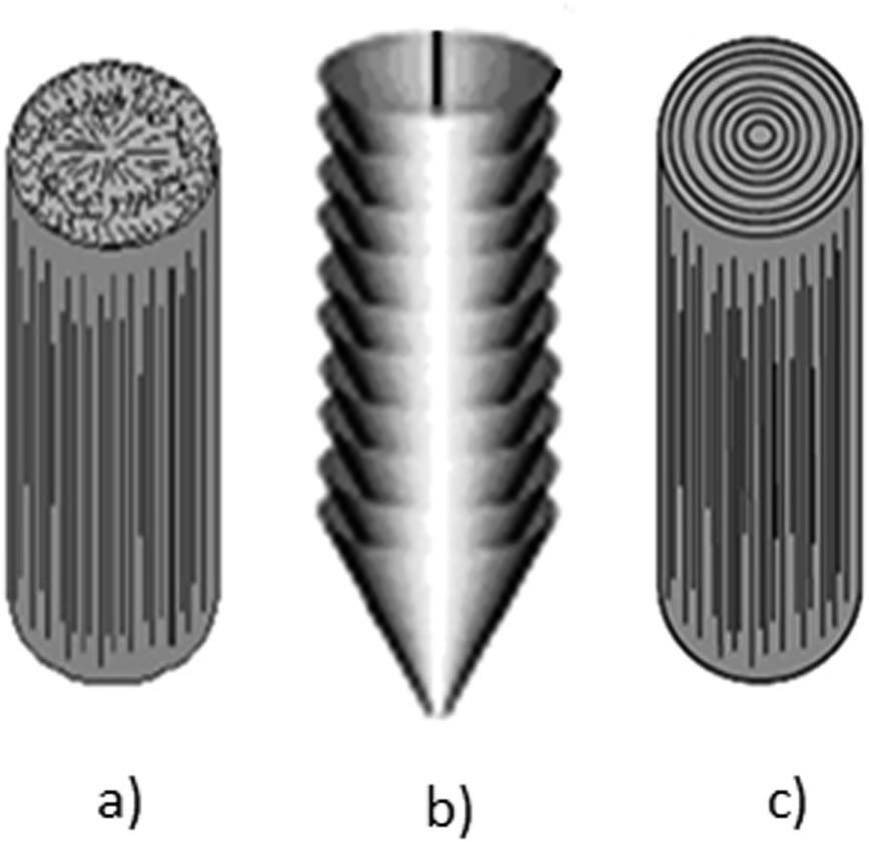
Structure of carbon fibers (a), carbon nanofibers (b), vapor grown carbon nanofibers (c).

Carbon fibers (CF) are described as the materials containing at least 90–92 wt% of carbon produced by the controlled pyrolysis of suitable fiber fabrics consisting of extremely thin fibers of about 5–10 μm in diameter (Fig. 12a). The carbon atoms in CF are bonded together in microscopic crystals that are more or less aligned parallel to the long axis of the fiber. The crystal alignment makes the fibers exceptionally strong for their size. They exhibit low weight and high mechanical strength, but poor thermal expansion coefficient. Their lower magnetism and high electric conductivity increases the impedance mismatching in electromagnetic shielding due to the increase of their skin depth.^48^ It was reported that activation of CF could significantly improve multiple reflections with a longer propagation distance within the polymer composites, which results in the higher absorption of the EM waves.^187^ Carbon nanofibers (CNF) are defined as cylindrical nanostructures with graphene layers constructed in the shape of cups, cones or plates, with an average diameter of 10–500 nm and an average length of 0.5–200 μm (Fig. 12b). They are quasi-one-dimensional carbon materials between carbon fibers and carbon nanotubes. According to the structural characteristics, CNF can be categorized as hollow carbon nanofibers and solid carbon nanofibers. The combination of a low density, large specific surface area, high aspect ratio, good dimensional stability, flexibility, high modulus, mechanical strength, thermal stability and excellent thermal and electrical conductivities allow nanofibers to be used for EMI shielding applications, as well as for the fabrication of composites in vehicles and aerospace. Carbon nanofibers can be manufactured *via* the electrospinning of various polymer materials, followed by thermal treatment, which can influence their final properties, such as composition, diameter, flexibility, and electrical conductivity. The optimal heat treatment conditions are thus extremely important for specific applications. CNF fabricated with the chemical vapor deposition growth method exhibited a small number of defects and compact structure (Fig. 12c).

Dang *et al.*^188^ investigated the influence of carbon fiber reinforced polyacrylamide/wood fiber composite boards, which were manufactured by a mechanical grind-assisted hot-pressing, for EMI shielding. The results demonstrated that CF with an average diameter of 150 nm was distributed on wood fiber, which was encased by polyacrylamide. The CF/polyacrylamide/wood fiber composite (CPW) exhibited the overall EMI SE of 41.03 dB compared to that of polyacrylamide/wood fiber composite (only 0.41 dB), which meets the requirements for commercial applications. Moreover, the CPW composite also exhibited high mechanical strength. In the research work,^189^ the influence of composite preparation methods on the electrical conductivity, electromagnetic parameters and EMI SE of polymer/carbon fiber composites were studied. The composites were prepared by using an internal mixer, injection molding machine and screw extruder. The results revealed that the polymer/CF (70/30 wt%) composites prepared by injection molding exhibited the highest electrical conductivity and overall EMI SE. The reason of the high electrical conductivity of these composites can be attributed to their increased dielectric constants (*ε*′ and *ε*′′). The percolation threshold of the composites was reached at a lower concentration of carbon fibers, which was affected by the increased of CF length in the composites during the injection molding preparation process. The EMI shielding occurred mainly by dielectric loss, such as dipole polarization and interface polarization, between the filler and the matrix, resulting in the enhanced EMI absorption values. The contribution of absorption to the overall EMI SE was 85.1%, while the contribution of reflection was only 14.9%. Ameli *et al.*^47^ used injection-molding to prepare solid and foamed polypropylene/carbon fiber composites containing 0–10 vol% of CF. The effects of foaming on the microstructure, percolation threshold, in-plane and through-plane electrical conductivity, dielectric permittivity and EMI SE were investigated. The results showed that the application of foaming reduced the volume fraction of percolation threshold from 8.5 to 7 vol% of CF, significantly enhanced the through-plane conductivity, increased the dielectric permittivity and resulted in the enhancement of the overall EMI SE up to 65%. Moreover, the uniformity of the through-plane and in-plane conductivities, as well as the shielding effectiveness along the injection-molded composites were greatly improved by foaming. Li *et al.*^190^ prepared lightweight EMI shielding cellulose foam/carbon fibers composites by blending cellulose foam solution with long carbon fibers (LCF) and acid treated short carbon fibers (SCF) followed by freeze-drying. It was shown that carbon fibers form conductive networks, where SCF were oriented in the bubble cell wall and LCF penetrated through the bubbles. LCF/cellulose foams exhibited higher electrical conductivity and higher EMI SE. The foaming process significantly enhanced the overall EMI SE from 10 to 60 dB within the tested frequency range of 30–1500 MHz. Authors of work^191^ evaluated the shielding effectiveness of composites based on polyamide resin matrix containing steam activated carbon fibers in 2–18 GHz frequency range. They found out that the activation of CF leads to the improved EM absorption characteristics of composites.

Carbon nanofibers have also been reported as suitable materials for the preparation of composites for EMI shielding applications.^192,193^ Chauhan *et al.*^194^ examined EMI SE of carbon nanofiber-reinforced poly(ether-ketone) composites fabricated using a corotating twin screw extruder. Nanocomposites filled with 14 vol% of CNF exhibited up to −40 dB of EMI SE (>99.99% attenuation) within the Ka-band frequency range (26.5–40 GHz). The contribution of the absorption to the overall EMI SE was about −37 dB with very low reflection loss of approximately −3 dB. Moreover, the prepared nanocomposites showed high thermal stability and mechanical strength, which points out to their utilization as efficient EMI absorbers in aerospace, defense, as well as space applications. Nayak *et al.*^195^ introduced lightweight EMI shielding nanocomposites prepared by a simple solution mixing process of carbon nanofibers with the polysulfone (PSU) matrix. The results demonstrated that the application of CNF resulted in the increase of the thermal stability of nanocomposites, which may be explained due to the barrier effect of the well-dispersed carbon nanofibers to the thermal transfer in the PSU matrix. The investigation of the EMI SE revealed that the contribution of absorption to the overall shielding effectiveness plays an important role when compared to reflection. With the increase in thickness of the nanocomposites from 0.1 to 1 mm, the EMI SE was found to increase linearly from around 18 to 45 dB in the X-band frequency range. Formation of conductive network (mesh) by CNF in the PSU matrix was reported to be the key reason for the improvement of the shielding effectiveness and conductivity. The influence of the melt mixing conditions on the rheological, morphological, electrical characteristics, EMI SE and tensile properties of 7.5 vol% vapor grown carbon nanofiber/polyethylene (VGCNF/PE) composites was evaluated in the study made by Al-Saleh^196^ 7.5 vol% loading of VGCNF provides satisfactory EMI SE for the equivalent polymer composites. The composites were prepared by melt mixing, and the parts were fabricated by hot-compression molding. The increase of the mixing energy (mixing time and/or rotation speed) resulted in better dispersion and distribution of VGCNF within the polymer matrix. The effect of the mixing energy on the electrical properties and EMI SE was shown to be a function of the rotation speed (shear stress). The increase in the mixing energy from 70 to 2300 J ml^−1^ caused the decrease in EMI SE from 29.5 to 23.9 dB for composites compounded at 20 rpm. For composites prepared at 100 rpm, the increase of the mixing energy from 600 to 1700 J ml^−1^ decreased the overall EMI SE from 25.4 to 18.6 dB. No considerable influence on Young's modulus, yield stress and strain at break was observed for various processing conditions.

##### 5.3.4.3 Carbon nanotubes (CNT)

**Fig. 13:**
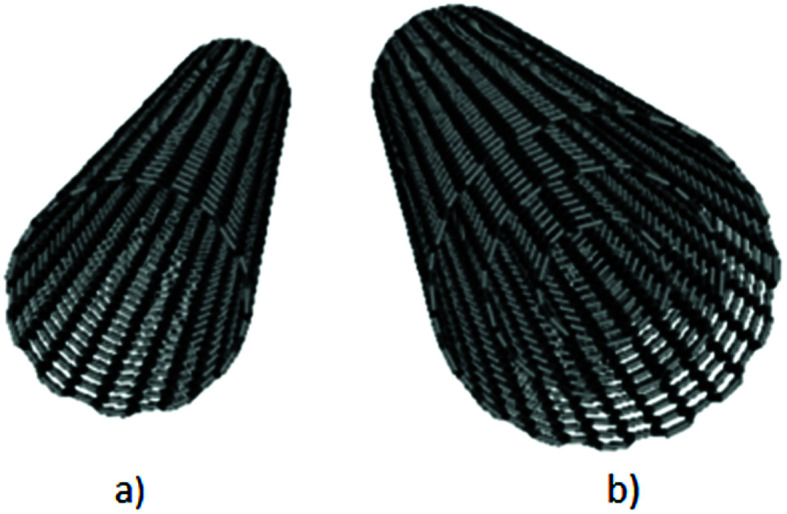
Structure of SWCNT (a) and MWCNT (b).

Carbon nanotubes (CNT) are allotropes of carbon with a cylindrical or tubular nanostructure containing six-membered carbon rings. These cylindrical carbon molecules have unusual properties, which are valuable for nanotechnology, electronics, optics and other fields of materials science and technology. In addition, owing to their extraordinary thermal conductivity, mechanical and electrical properties, carbon nanotubes find applications as additives to various structural materials. Carbon nanotubes are categorized as single-walled nanotubes (SWCNT) (Fig. 13a) and multi-walled nanotubes (MWCNT) (Fig. 13b). The electrical properties of SWCNT are different from MWCNT due to their smaller diameter and higher aspect ratio. As a result of this, both types exhibit different EMI SE characteristics when incorporated in polymer composites. The main drawbacks of SWCNT are the complicated fabrication process, extremely high conductivity and poor magnetic characteristics. These features limit the utilization of SWCNT as excellent microwave absorption materials. MWCNT are the more preferred types of carbon nanotubes. MWCNT consists of multiple layers of graphite superimposed and rolled into a tubular shape. The structural disorders, which appear in MWCNT during the production process, are responsible for the unique optic, electrical properties and also other features. These structural disorders can be atomic defects, Stone–Wales defects or in the form of vacancies and bonding defects.^48^ In general, carbon nanotubes are the strongest and stiffest materials yet discovered in terms of tensile strength and elastic modulus. Due to its low weight, small diameter, high aspect ratio, easy percolation, exceptional conductivity and good mechanical strength, CNT has significant advantages over conventional carbon-based fillers, and high EMI SE can be easily achieved at relatively low contents in polymer matrices.^59,197^

Polley *et al.*^198^ studied the complex conductivity and EMI SE of single-walled carbon nanotubes distributed in polyvinyl alcohol matrix in the THz frequency range. Terahertz time-domain spectroscopy (THz-TDS) was performed in transmission geometry within 0.3–2.1 THz frequency range. The transmittance spectra demonstrated a possibility of applications of PVA/SWCNT composite materials as low-bandpass filters in the THz frequency region. Shielding effectiveness of composites showed a linear relationship with increasing content of SWCNT in the polymer matrix at a particular probing frequency. The results of experiments performed by Li *et al.*^199^ demonstrated that composites based on epoxy resin and SWCNT can be used as effective, lightweight EMI shields. The highest EMI SE was recorded for the composite filled with 15 wt% of SWCNT, reaching 49 dB at 10 MHz and showing 15–20 dB over the frequency range of 500 MHz to 1.5 GHz. The shielding efficiency was found to correlate with the dc conductivity, and this frequency range was observed to be dominated by reflection. Peng *et al.*^200^ used a theoretical model based on logarithmic rule to investigate the relative complex permittivity and complex conductivity of SWCNT-filled poly(ethyl methacrylate) composite films in the frequency range of 0.3–18 GHz. The intrinsic electrical properties of the polymer matrix and SWCNT, as well as the particle size and shape were taken into account. The real and imaginary parts of the composite conductivity were both found to increase dramatically with the increasing content of SWCNT at microwave frequencies, while the former was more sensitive. The results also revealed that the application of a small amount of SWCNT in the polymer matrix resulted in the explicit increase of the complex permittivity, complex conductivity and dielectric loss tangent of the composites. This can be attributed to the generation of the resistance–capacitance network and introduction of conducting filler paths to the composites. Zhang with his co-workers^201^ demonstrated simultaneously improved EMI shielding performance and mechanical characteristics in a segregated composite based on polypropylene and carbon nanotubes. This was achieved *via* a facile and eco-friendly technical approach that included melt mixing, pre-coating and injection molding. The segregated CNT/PP composite loaded with 3.5 wt% of CNT exhibited EMI SE of 32 dB, which means 130% and 30% improvement in comparison to the corresponding PP/CNT composites fabricated by conventional injection molding (14 dB) or compression molding (25 dB), respectively. The segregated composite prepared by eco-friendly approach also exhibited a higher elongation at break, and comparable Young's modulus and tensile strength when compared to conventionally prepared composites. Feng *et al.*^202^ investigated both single-walled carbon nanotubes and multi-walled carbon nanotubes as the fillers in cellulose composite films, which were fabricated by a green route using sodium hydroxide/urea aqueous solution. It was observed that carbon nanotubes formed layered conductive networks in the cellulose matrix during the process of drying. The results revealed that composite films filled with SWCNT were found to be more effective for EMI shielding in 12–18 GHz frequency range when compared to MWCNT/cellulose films. The composite with 10 wt% of SWCNT reached the overall EMI SE of 32.5–40 dB. Considering the density and the thickness of the material, the SWCNT/cellulose film showed an extremely high specific EMI SE of around 7678 dB cm^2^ g^−1^, which can be attributed to the unique layered structures of the carbon nanotubes.

**Fig. 14:**
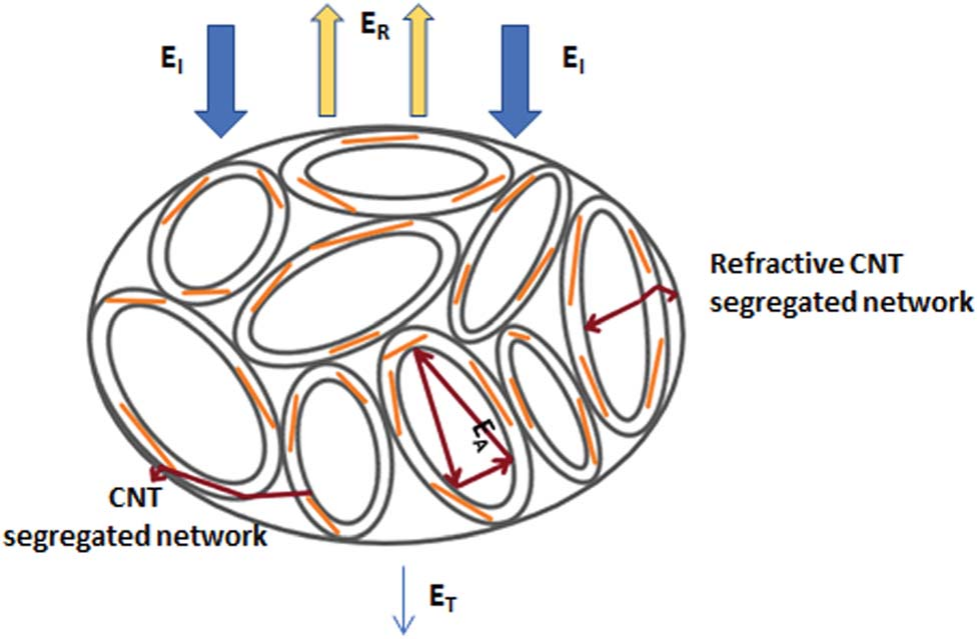
Schematic illustration of the EMI shielding mechanisms for the segregated network of PE/CNT composites (modified from ref. 208 with permission from MDPI, copyright 2020).

**Fig. 15:**
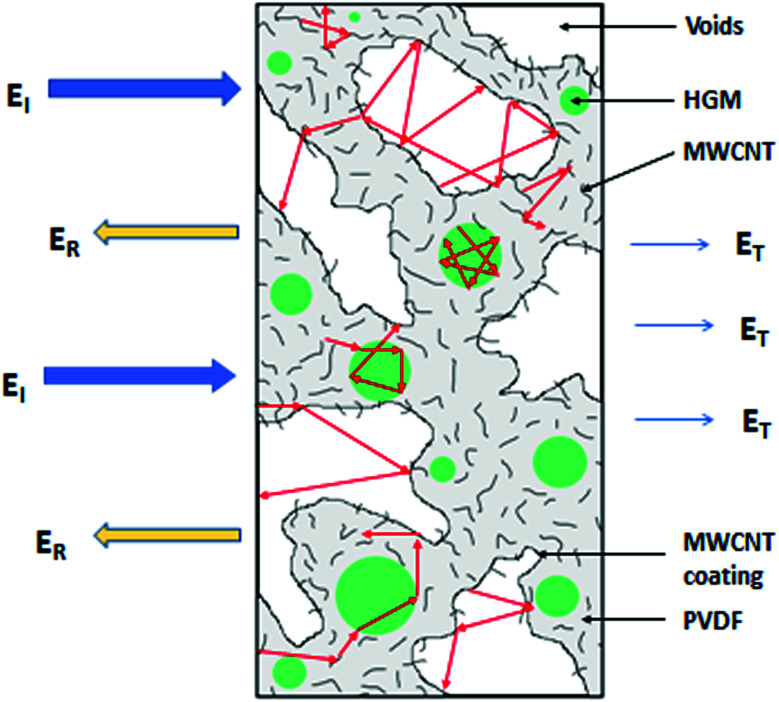
Schematic illustration of the EMI shielding mechanisms for porous PVDF/HGM/MWCNT composites (modified from ref. 212 with permission from Springer, copyright 2018).

MWCNT has been tested and examined in many conductive and EMI shielding materials research studies, owing to their outstanding electrical conductivity.^203^ The concentric multiple tube structures of MWCNT are the main reason for the reinforcement possessing high conductivity. Authors of the research work^204^ utilized the concept of preferential distribution and double percolation to prepare conductive polymer composites that absorb EM waves. Poly(ethylene-*co*-methyl acrylate) and ethylene–octene copolymer EMA/EOC (50/50) wt% binary blend was filled with MWCNT as a functional filler. The composites exhibited excellent EMI SE (composite filled with 3 wt% of MWCNT showed EMI SE ∼−18.66 dB, while the composite containing 15 wt% of MWCNT exhibited EMI SE ∼−34.06 dB in the X-band frequency range), combined with good thermal and mechanical properties. The results also revealed that MWCNT were preferentially distributed in the EMA phase. The co-continuous morphology of the EMA/EOC blend composite systems and preferential distribution of the filler-facilitated double percolated the conductive network, which thus enhances electrical conductivity. In the work done by Chizari and his co-workers,^205^ 3D printing was introduced to fabricate a light, semi-transparent and conducting structure to be tested for EMI shielding. Highly conducting 3D printable inks showing electrical conductivity up to ∼5000 S m^−1^ were prepared from nanocomposites based on polylactic acid filled with carbon nanotubes. The results demonstrated a significant enhancement of the specific EMI SE for PLA/CNT nanocomposites printed as 3D scaffolds in comparison to CNT/PLA nanocomposites hot-pressed in solid form (∼70 dB g^−1^ cm^−3^*vs.* ∼37 dB g^−1^ cm^−3^). The light transmittance of the scaffolds varied from ∼0% to ∼75% by changing the inter-filament spacing, number of layers and printing patterns. The conductivity of the fabricated ink was reported to be the highest among the other 3D printable polymer composite inks reported so far, and this was the first published study on EMI shielding by application of the 3D printing approach. The results also revealed the possibility for the preparation of EMI shields having light and transparent structures with utilization in applications such as portable electronic devices, aerospace systems or smart fabrics. Otero-Navas *et al.*^206^ investigated the effect of carbon nanotubes on the morphology, rheology and broadband dielectric properties of polypropylene/polystyrene composites with different polymer ratio (PP/PS – 10 : 90, 50 : 50 and 90 : 10). It was revealed that MWCNT were situated at the interface and inside the polystyrene phase, regardless of the polymer composite ratio. It was proposed that MWCNT situated at the interface acted as bridges between the PS/MWCNT domains, supporting the coarsening of the PS/MWCNT domains. The results also revealed that with increasing MWCNT content, double percolation was observed in the composite with PP/PS ratios of 50 : 50 and 90 : 10. This provided the possibility to tune the dielectric properties of the prepared nanocomposites. At the end, it was stated that manipulating the blend morphology paves the way for the fabrication of composites with capacitive or dissipative features. Arjmand *et al.*^207^ carried out a comparative study of the electrical conductivity, permittivity and EMI shielding performance of the compression-molded and injection-molded PS/MWCNT composites. The results showed that injection-molded nanocomposites, in which MWCNT were aligned, exhibited lower EMI shielding characteristics in comparison to compression-molded nanocomposites with a random distribution of MWCNT. This was attributed to the lower probability of MWCNT to make contact with adjacent MWCNT particles in the injection-molded nanocomposites. The compression-molded composites exhibited higher electrical conductivity and lower percolation threshold when compared to corresponding injection-molded composites. The lower shielding efficiency of the injection molded composites can be attributed to the lower electrical conductivity, imaginary permittivity (ohmic loss) and real permittivity (polarization loss). Finally, the authors stated that EMI shielding does not require filler connectivity. However, the filler connectivity increases EMI SE. Polyethylene-based composites with segregated carbon nanotubes network, prepared by hot compression of a mechanical mixture of PE and CNT powders, were investigated for EMI shielding in the study performed by Vovchenko *et al.*^208^ It was shown that the formation of a segregated filler network in the polymer composites resulted not only in a substantial decrease of percolation threshold concentration, but was also responsible for the outstanding electrical properties of the composites. This means 10^−1^ S m^−1^ at 0.5–1 wt% and 10 S m^−1^ at 6–12 wt% of CNT. The investigation of the complex impedance in the frequency range of 1 kHz to 2 MHz revealed that the real part of the dielectric permittivity changed from positive to negative in the electrically percolated composites, suggesting the metal-like behavior of the filler segregated network. However, in contrast to the reflective metals, the high EMI SE of the PE-based composites within the 40–60 GHz frequency range was due to absorption coursed by multiple reflection on every PE/CNT segregated network interface, followed by EMI absorbed in each isolated PE granule enclosed by conductive CNT shells (Fig. 14). Composite materials with segregated conductive filler networks exhibit high conductivity, resulting in the efficient attenuation of EM radiation by both reflection and absorption processes.^209,210^ Cui *et al.*^211^ developed a segregated polylactic acid PLA/CNT composite with high electrical conductivity and improved shielding effectiveness of 35.5 dB in the X-band frequency range at CNT content of 1.0 wt%. In the study,^212^ hollow glass microspheres (HGM) were first introduced to prepare functional polymer composites based on poly(vinylidene fluoride) and MWCNT. Porous PVDF composites with MWCNT and hollow glass microspheres were prepared by molten mechanical mixing, hot-pressing and selective etching procedure. Inorganic hollow microspheres were found to be low-cost materials with good chemical stability, low weight and high-temperature resistance. They are ideal raw materials for the preparation of absorbing polymer composites as their micron size promotes the ease of combination with the polymer matrix. The HGM embedded into the polymer matrix play an important role in reflecting and multiple scattering of the incident EM waves (Fig. 15). The synergistic effect between the HGM and the MWCNT conductive networks, governed by a dominant absorption mechanism, resulted in a significant improvement to the EMI SE of the porous composites. When compared to the composite with the absence of HGM (25.27 dB of EMI SE), the composite containing 10 wt% of MWCNT and 2 wt% of HGM exhibited an improved EMI SE of ∼43.03 dB within the X-band frequency range and enhanced specific EMI SE of ∼61.47 dB cm^−3^ g^−1^. The application of HGM resulted in the decrease of the thermal conductivity of the composites by lowering the active surface area that facilitates efficient heat transfer. By introduction of 2 wt% HGM, the thermal conductivity of the porous composite fell by almost 47%, from 0.305 to 0.162 W m^−1^ K^−1^. The study^213^ was focused on the development of MWCNT-filled polypropylene composites and carbon nanofiber composite mats. MWCNT/CNF composite mats were designed by centrifugally spinning mixtures of MWCNT suspended in poly(vinyl alcohol) solutions. The generated nanofibers were subsequently dehydrated under sulfuric acid vapors, followed by heat treatment. The inter-layered composites were prepared using a nanoreinforced PP matrix, and then filled with CNF composite mats. The developed inter-layered structures exhibited improved electrical conductivity and shielding effectiveness. In an eight-layer flexible composite, which was composed of 18 wt% carbon content, 12 wt% of MWCNT and 70 wt% of PP, the electrical conductivity was found to be 6.1 S cm^−1^, while the EMI SE was 52 dB at 900 MHz for the sample with a thickness of 0.65 mm. The study provides new opportunities for the preparation of novel lightweight materials, which can be used in communication systems. Al-Saleh *et al.*^45^ investigated the microstructure, complex permittivity, electrical conductivity and EMI SE of the nanocomposites based on acrylonitrile–butadiene–styrene (ABS) terpolymer and three different types of carbon nanostructures, namely multi-walled multiwalled carbon nanotubes, carbon nanofibers and high structure carbon black. The composites were prepared by solution blending and characterized by the uniform dispersion of the fillers within the ABS matrix. It was revealed that at the same filler content, permittivity, electrical conductivity and EMI SE of composites decreased in the following order: MWCNT > CNF > HS-CB. Composites filled with MWCNT were found to reach the lowest percolation threshold and the highest overall EMI SE, which can be attributed to the higher aspect ratio and conductivity of MWCNT in comparison with CNF and HS-CB. At 5 wt% of the filler loading, the EMI SE of MWCNT based composite was 2 times higher than that of the CNF-based composite and 7 times higher than that of the HS-CB based composite. Moreover, at MWCNT content of 2 wt%, the composite exhibited EMI SE of 20 dB in the X-band frequency range. By increasing the MWCNT content to 15 wt%, the EMI SE increased up to 50 dB. It was also reported that the EMI SE of the tested composites was absorption-dominated, regardless of the type of filler.

##### 5.3.4.4 Graphite (GR)

**Fig. 16:**
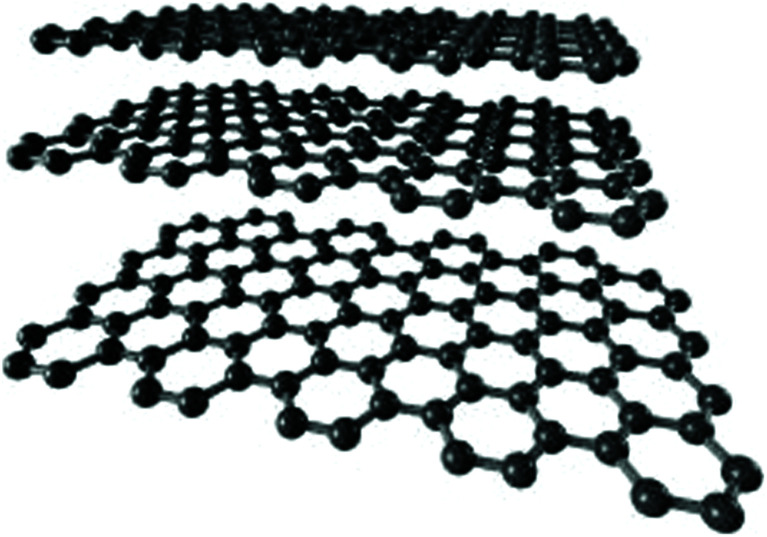
Structure of graphite.

Graphite (GR) has a layered lattice structure consisting of sheets of carbon atoms arranged in a regular hexagonal pattern (Fig. 16). It is a crystalline material, in which the sheets are stacked parallel to one another in regular fashion. The intermolecular attractive forces between the sheets are relatively weak physical van der Waals bonds, making graphite soft and brittle. In the lattice, carbon atoms are bonded together by covalent carbon–carbon bonds. The low price, good electrical conductivity, good thermal and mechanical stability make it an attractive filler in optic, electronic and energy devices. Over the last years, more and more attention has been paid to the expanded graphite (EGR). Expanded graphite, obtained by heat treatment, consists of a small stack of graphite layers and exhibits high mechanical stability, low resistivity and low cost. The biggest disadvantage of graphite and expanded graphite is their poor magnetic characteristics, which restricts their utilization in EMI shielding applications.

Panwar *et al.*^214^ investigated the dielectric properties and EMI shielding characteristics of composites based on polypropylene and graphite in the low and radio frequency ranges. The PP/GR composites were prepared by blending and hot compression mold technique. The dielectric constant and electrical conductivity of the composites followed the power law model of percolation theory. The percolation threshold concentration of GR was found to be 5 wt%. The results revealed that the main mechanism of shielding of composite materials was due to reflection. The maximum EMI SE of the composites (44.12 dB) was reached at 2.76 GHz. The proposed PP/GR composites showed high dielectric constant and dissipation factor in the low and radio frequency ranges. Finally, the authors stated that PP/GR composites could be used in decoupling capacitors, charge storing devices and EMI shielding applications. Microcellular polylactic acid PLA/GR nanocomposite foams, prepared by injection molding, with enhanced electrical and mechanical properties for ultra-efficient EMI shielding applications were deeply examined in the study.^215^ A microcellular PLA/GR nanocomposite foam with a thickness of 2.0 mm exhibited excellent EMI shielding performance with the overall EMI SE of up to 45 dB, which was reported to be absorption-dominated. The graphite reorientation during foaming resulted in a dramatic increase in the electrical conductivity of the microcellular PLA/GR nanocomposite foam by almost six orders of magnitude when compared to the unfoamed composite. Moreover, the microcellular PLA/GR nanocomposite foam showed outstanding mechanical properties (very high modulus and specific strength, as well as super-ductile fracture behavior). Kenanakis *et al.*^216^ used a solution process to synthesize polystyrene PS/GR composite films with a thickness of 200 μm, and investigated them for EMI shielding in the C-band frequency range (3.5–7 GHz). It was found out that the composite containing 39.1 wt% of graphite is a suitable candidate for practical applications, as it exhibited higher stiffness compared to plain PS and adequate tensile strength (>35 MPa), in line with the efficient EMI shielding performance (overall EMI SE = 10–15 dB, absorbance ∼80%). In the scientific study,^217^ the high performance composites based on polyphenylene sulphide (PPS) and graphite flakes (GRF) were fabricated by a hot pressing technique. The percolation threshold was obtained at 5 wt% of GRF, and the SEM analysis showed a 3D conductive network of GRF within the PPS matrix. The dissipation factor and dielectric constant (evaluated in the 100 kHz to 15 MHz frequency range) of composites above the percolation threshold concentration significantly increased, but decreased with an increase in frequency. Significant enhancement in the electrical properties suggests that the prepared composites may be useful for EMI shielding applications, particularly at high temperatures. Portes *et al.*^41^ investigated the EMI SE of composites based on silicone rubber and natural graphite flakes (NGRF) in the X-band frequency range. The authors claim that the concentration, variation and combination of the particle sizes of NGRF showed a considerable impact on its electromagnetic characteristics. Composites containing NGRF with larger particle sizes exhibited slightly better performance on the SE_T_ when compared to the polymer composites produced with the smaller ones. The variation and combination of the particle sizes demonstrated a promising influence on the composite EMI SE, which allows for controlling the overall shielding performance within the 8.2–12.4 GHz frequency range. Vovchenko *et al.*^218^ performed a comparative study of epoxy resin filled with thermo-exfoliated graphite (TEGR) of various dispersities to investigate the influence of filler particle morphology on the EMI shielding characteristics of the composites within the 25.86–37.5 GHz frequency range. The achieved results showed that the composites containing TEGR exhibited improved electrical conductivity and EMI SE when compared to the composites with sonicated TEGR. Gogoi *et al.*^219^ investigated EMI SE of double layer microwave composite absorbers, designed and developed with a paired combination of 5–10 wt% of expanded graphite/novolac phenolic resin (EGR/NPR) in the X-band frequency range. The maximum −25 dB and −30 dB absorption bandwidths of 2.47 GHz and 1.77 GHz, respectively, were recorded for the double layer structures with (5–8 wt%) EGR/NPR composites having thickness of 3.2 mm, while −10 dB bandwidth covers the whole X-band frequency range.

##### 5.3.4.5 Graphene (GN)

**Fig. 17:**
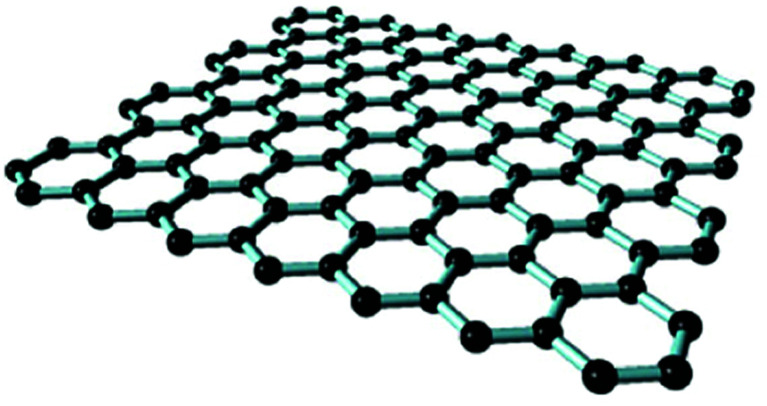
Structure of graphene.

Graphene (GN), a two-dimensional allotrope of carbon consisting of a single layer of sp^2^ hybridized bonded carbon atoms, is a durable planar nanostructure, in which carbon atoms are arranged in the form of a honeycomb hexagonal crystal lattice (Fig. 17). Due to the atomically thick 2D structure and ultrahigh specific surface area, the percolation threshold can be reached at very low content. The exceptional charge carrier mobility, excellent thermal and electrical conductivity, as well as unique mechanical, optical and magnetic properties make graphene a very promising candidate for electronics, optoelectronics, sensors, energy storage, structural materials and EMI shielding applications.^220^ Graphene/polymer composites have been residing at the frontier of functional shielding materials with great potential for overwhelming the challenges related to the physical, chemical and mechanical performance, durability and functionality in the field of electromagnetic shielding. On the other hand, the excellent high carrier mobility and lack of surface functionalities can be detrimental for EMI absorption, forming impedance mismatching between the material and air. Therefore, derivatives of graphene, such as graphene oxide (GO) and reduced graphene oxide (RGO), have been widely used as alternatives to graphene in practical applications.

Ha *et al.*^221^ developed multi-functional polydimethylsiloxane/graphene composites (PDMS/GN) with EMI shielding characteristics and de-icing properties by three-roll milling. The filler was homogeneously dispersed within the polymer matrix as a result of which, the electrical conductivity of the composites increased up to 10^2^ S m^−1^, and EMI SE was enhanced to the level required for applications in the EMI shielding systems (25 dB in the frequency range of 1–3 GHz). Due to the high electrical conductivity, the composites could be heated rapidly from room temperature up to 200 °C in 50 s by electrical heating with low electric power. Moreover, the composite films were suitable for applications in de-icing units because of their fast and homogeneous heating performance. Shen *et al.*^222^ fabricated strong, flexible sandwich-like structure composite films based on polyester non-woven fabric as a reinforcing interlayer and thermoplastic polyurethane with high graphene content as a conductive coating layer. The film containing 20 wt% of GN with a total coating thickness of 50 μm exhibited outstanding strength and mechanical flexibility, as well as qualified bandwidth of EMI SE ≥ 20 dB as wide as 49.1 GHz within a broadband 5.4–59.6 GHz frequency range. The results also revealed that the 3D saw-tooth folding of the composite films efficiently increased the EMI shielding performance and reduced the reflection loss. Multifunctional cotton fabrics with electrical and EMI shielding characteristics fabricated *via* layer-by-layer electrostatic self-assembly approach were examined in the work.^223^ Chitosan (CS) was used as a polycation with GN introduced by solution blending, and poly(sodium 4-styrenesulfonate) (PSS) as polyanion was deposited on the fabric substrate, followed by the CS/GN layer alternatively. The results revealed that the 10-layer-deposited fabric achieved an electrical conductivity of 1.67 × 10^3^ S m^−1^, and a very high EMI SE of 30.04 dB in the tested frequency range of 30 MHz to 6 GHz. The absorption-dominated shielding performance originated from the multiple reflection and scattering from the graphene network structure. It was also found that the prepared fabrics exhibited good physical–mechanical properties and electro-heating behavior. Gao *et al.*^224^ fabricated nacre-mimetic PDMS/GN composites with extremely low graphene content for high-performance EMI shielding. The unique bi-directional freezing technique was introduced to prepare nacre-mimetic 3D conductive GN network showing biaxial aligned lamellar structure. PDMS/GN composites with a nacre-mimetic, highly aligned network exhibited anisotropic conductivity, mechanical characteristics and therefore excellent EMI shielding efficiency at a very low graphene content. It was demonstrated that biomimetic composites containing only 0.42 wt% of GN showed an improved EMI SE of 65 dB after annealing the GN aerogels at 2500 °C, which is very close to the copper foil. Moreover, the specific shielding effectiveness of these low-density composites was even higher when compared to that of metal foils or solid materials filled with a high content of conductive fillers.

**Fig. 18:**
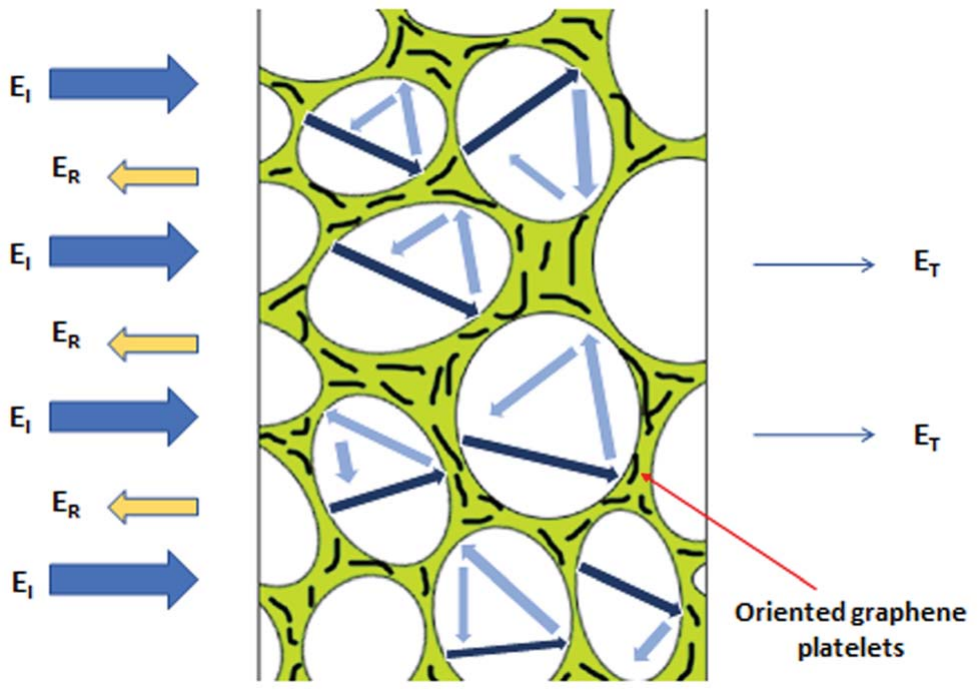
Schematic illustration of the EMI shielding mechanisms for the PEI/GN composite foams (modified from ref. 227 with permission from the American Chemical Society, copyright 2013).

Polymer foams containing conductive fillers have been widely applied in a range of applications such as in lightweight and flexible actuators, electrostatic discharge, and EMI shielding. In the work,^225^ compressible and ultralightweight PU/GN composite foams were developed by solution dip-coating of graphene on polyurethane sponges having a network structure with high porosity. The composite foam with extremely low density of 0.027–0.030 g cm^−3^ exhibited absorption-dominated EMI shielding performance of 39.4 dB at 10 wt% of GN and 60 mm of composite thickness. It was reported that the conductive dissipation, as well as multiple reflection and scattering of EM radiation within the 3D conductive graphene network, contributed to the enhanced absorption shielding. Chen *et al.*^226^ reported that the PDMS/GN composite foams with 3 mm thickness synthesized *via* the CVD technique showed EMI SE of 21 dB at 8 GHz, and the specific EMI SE was found to be 500 dB cm^−3^ g^−1^ at 0.8 wt% of GN. The results also revealed that the composite foams maintain stable EMI SE characteristics even when exposed to repeated bending for around 10 000 times. Ling *et al.*^227^ reported a facile method to fabricate microcellular, lightweight polyetherimide (PEI)/graphene composite foams with a density of 0.3 g cm^−3^ using a phase separation approach induced by water vapor, with the absence of any foaming agent. It was demonstrated that the strong extensional flow formed during cell growth stimulated the orientation of GN on the cell walls (Fig. 18). Moreover, the foaming process considerably increased the specific EMI SE from 17 to 44 dB cm^−3^ g^−1^, which was reached at very low GN content. PEI/GN nanocomposite foams also showed high Young's modulus and low thermal conductivity.

##### 5.3.4.6 Graphene oxide (GO)

**Fig. 19:**
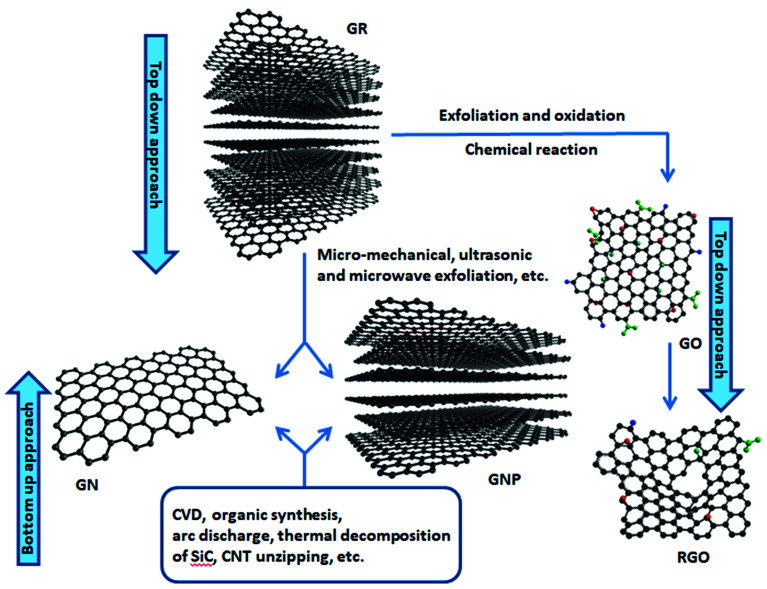
Schematic illustration of the methods for synthesis of graphene oxide and reduced graphene oxide (modified from ref. 55, with permission from Elsevier, copyright 2018).

Graphene oxide (GO) is the 2D oxidized form of graphene decorated with various functional oxygen groups, such as epoxy, carboxyl, carbonyl, hydroxyl, diol or ketone, preferentially on the basal planes (bottom and top) and graphitic edges.^137,138^ It is a single-atomic layered material, which is produced by oxidation of graphite using strong oxidizing agents. Due to the polar oxygen groups, it is hydrophilic, which enables it to extract GO after water sonication. It is dispersible in water and in a variety of inorganic and organic solvents, in which the functional groups of GO can be easily modified by other materials. It is also considered easy to process. Graphene oxide is not a good conductor, but its heat and light treatment or chemical reduction can restore most properties of pristine graphene. It can be reduced in solutions, as well as in the form of thin films using a variety of reducing conditions, and reduction converts GO into the excellent electrical conductive material. For the synthesis of graphene oxide, four basic methods are generally applied, *i.e.*, Hofmann, Brodie, Staudenmaier and Hummers, which are thermal or mechanical exfoliation, chemical vapour deposition (CVD) or epitaxial growth (Fig. 19). The effectiveness of the oxidation process is often determined by the oxygen/carbon ratios of GO. Graphene oxide has become one of the most popular 2D materials available and has attracted much interest thanks to its outstanding properties, such as large surface area, excellent mechanical stability, tunable electrical, optoelectronic and transport properties, which hold great promise for versatile applications in the field of photocatalysis, memory devices, solar cell, energy storage/conversion, chemical sensors, optoelectronics, drug delivery, as well as composite materials. The excellent compatibility of graphene oxide with polymers makes it an attractive filler for the preparation of composites with significantly enhanced electrical and thermal conductivity, tensile strength or elasticity. The excellent mechanical and reinforcement properties of polymer composites arise from the oxygen-containing functional groups of GO, which help to increase the interfacial bonding and to transfer stresses from the polymer matrix to the filler and *vice versa*.^68,228,229^

Kumar *et al.*^230^ studied a self-aligned asymmetric conductive composite film fabricated *via* casting of poly(vinylidene-hexafluoropropylene)/graphene oxide dispersion, followed by hydriodic acid reduction at low temperature. It was found out that the composite film showed high orientation of GO along the direction of the composite surface. On the other hand, graphene sheets were asymmetrically dispersed along the film thickness direction. The asymmetric surface properties of the film resulted in the difference of the surface resistivity response. The bottom surface resistivity (5 ohm) is about 4 times lower than the top surface resistivity (21 ohm). The asymmetric highly electric conductive composite thin film revealed an efficient overall EMI SE of 30 dB in the X-band frequency range. In the study,^231^ graphene oxide was synthesized from graphite powder by Hummer's method, and it was subsequently functionalized by silanized silica nanoparticles. A solution method was used to prepare polyetherimide (PEI)/functionalized graphene oxide (FGO) nanocomposites, in which FGO was dosed in a concentration scale of 0.5–3 wt%. The results showed that the nanocomposites filled only with 1.5 wt% of FGO exhibited EMI SE of 21–23 dB in the frequency range of 8–12 GHz. The dynamic-mechanical analysis revealed the improvement in the storage modulus and the glass transition temperature of the PEI/FGO nanocomposites. In comparison with pure polyetherimide, the dielectric constant of the nanocomposite containing 1.5 wt% of FGO increased seven times and the dielectric loss decreased 14 times. The nanocomposite films also demonstrated good mechanical and dielectric characteristics, and excellent EMI shielding efficiency over a wide range of frequency and temperature.

Owing to the flexible 3D structure, the high electrical conductivity and great potential for outstanding EMI shielding performance, graphene sponges have attracted considerable interest over last years. However, their EMI shielding performance suffers from poor durability and low elasticity. Hu *et al.*^232^ demonstrated the potential of self-assembled polyurethane/graphene oxide sponge composites, synthesized using a two-step hydrothermal chemical reduction process, for EMI shielding. The PU/GO lightweight and durable composite foam showed superior absorption-dominated EMI SE of 38 dB in the X-band frequency region, and high specific EMI SE of 969–1578 dB cm^−2^ g^−1^. The excellent EMI shielding characteristics originate from the superconductivity of GO and highly porous structure of the PU/GO sponge. It was reported that the polyurethane sponge behaves as a sturdy scaffold for graphene to shape its 3D structure.

##### 5.3.4.7 Reduced graphene oxide (RGO)

Reduced graphene oxide (RGO) has a heterogeneous structure consisting of graphene-like basal plane, which is decorated with additional structural defects and populated with places containing oxidized chemical groups and heteroatoms.^233^ It exhibits similar mechanical, conductive and optoelectronic characteristics to pristine graphene. RGO has become a more appealing material not only for its unusual electric and thermal conductivity, good flexibility, mechanical and barrier properties, but also for its more attractive pricing and manufacturing processes. The graphene-like properties make RGO a highly desirable material to be used in sensors, environmental, biological or catalytic applications, as well as in optoelectronics, storage devices and composite materials. Moreover, the functional groups and structural defects within the RGO sheet help to improve the impedance mismatch, electronic dipole polarization and defect polarization relaxation. Those features of the reduced graphene oxide shift towards the penetration and absorption of the electromagnetic radiation waves, rather than reflection. When compared to graphite or carbon nanotubes, RGO exhibits higher magnetic/dielectric loss by means of microwave absorption.^234^ The mentioned attributes have recently made RGO one of the most studied materials for EMI shielding applications.^235–238^ There have been several reduction processes developed for the synthesis of reduced graphene oxide from graphene oxide based on the thermal, chemical or electrochemical approaches. Reduction of GO to produce RGO is an extremely vital process as it has a significant impact on the quality of RGO produced. Some of the reported processes include:

- treatment of GO with hydrazine hydrate at 100 °C for 24 hours

- exposure of GO to hydrogen plasma for several seconds

- exposure of GO to powerful pulsed light from xenon flashtubes

- heating of GO with urea as an expansion–reduction agent

- direct heating of GO in a furnace to very high temperature.

Kumar and Kaur^239^ reported a comparative study based on the influence of different novel reduction techniques for the preparation of devices for EMI shielding within the X-band frequency range. They implemented hydrazine hydrate, thermal annealing, swift heavy ion irradiation and low energy ion beam reduction method. The results showed that the room temperature electrical dc conductivity was in good correlation with the extent of reduction of GO. The sample treated with swift heavy ion irradiation of Ag^8+^ at 100 MeV and hydrazine hydrate exhibited the highest dc electrical conductivity, which resulted in higher EMI SE of 55.29 dB and skin depth of 0.019 cm. It was also shown that the skin depth decreased with an increase in conductivity. This points to a strong potential of this RGO for high-performance EMI shielding applications. Nanocomposites prepared by application of 0.5–2.5 wt% of reduced graphene oxide, synthesized by the Hummers method, into the polyetherimde matrix were investigated for EMI shielding within the X-band frequency range in the work done by Sawai *et al.*^240^ It was shown that the EMI SE of the composites was frequency-dependent, and it increased with the increase in frequency. The electrical conductivity, thermo-mechanical properties and shielding effectiveness were enhanced with increasing content of RGO. The nanocomposite filled with 2.5 wt% of RGO exhibited EMI SE of 22–26 dB over 8–12 GHz. Gao and his co-workers^241^ synthetized acrylonitrile–butadiene–styrene copolymer/reduced graphene oxide nanocomposites through a latex technology to form conducting ABS with high efficiency at low RGO loading. Owing to π–π interactions between polystyrene fragments in ABS and reduced graphene oxide, particles of RGO were homogeneously distributed on the ABS microsphere surface to generate a conducting network after compression. The ABS/RGO nanocomposites exhibited significant enhancement in electrical properties (electrical conductivity of 0.09 S m^−1^) at a very low percolation concentration of 0.062 vol% in comparison with reported ABS composites fabricated *via* solution methods or melting. In addition, physical properties, such as the mechanical strength and thermal conductivity of nanocomposites were enhanced as well. Ahmad *et al.*^242^ used a melt blending method for the fabrication of RGO reinforced poly(lactic acid)/poly(ethylene glycol) nanocomposites. The improved thermo-mechanical characteristics of the composites were attributed to the good adhesion between the PLA/PEG matrix and RGO nanoparticles. It was reported that RGO behaves as a heat barrier, improving the overall thermal stability. The increasing content of RGO resulted in a significant increase of dielectric properties of the composites. The nanocomposites reached an EMI SE value higher than 20 dB in the X-band frequency range. The effective absorbance of the composites increased with filler loading increase. The results also revealed increased power loss with the increase in frequency, and conversely decreased with the increase in RGO content. Ultrathin, highly aligned cellulose nanofiber (CNF)/reduced graphene oxide composite films with remarkable EMI shielding characteristics and strong anisotropy in thermal conductivity were prepared by vacuum-assisted filtration, followed by subsequent hydroiodic acid reduction.^243^ The obtained CNF/RGO (50 wt%) composite film with only 23 μm in thickness exhibited excellent conductivity of 4057.3 S m^−1^ and great EMI SE of 26.2 dB, owing to self-alignment and uniform dispersion into the RGO layered structure. In the scientific work,^244^ flexible composite films based on poly(vinyl alcohol)/reduced graphene oxide coated activated carbon (PVA/RGO/AC) with extremely low graphene content were fabricated by using AC as substrates and segregators. Decoration of AC with graphene to form an individual RGO-sheet-coated AC structure resulted in a dramatic increase in the AC conductivity and prevented the agglomeration and restacking of graphene. The percolation threshold of the PVA/RGO/AC composites was reached at only 0.17 wt% of RGO/AC. The high electrical conductivity of 10.90 S m^−1^, excellent absorption-dominated EMI SE of 25.6 dB and the specific EMI SE of 17.5 dB mm^−1^ were achieved for the composite containing only 1.0 wt% of RGO. The outstanding electrical properties and impressive EMI shielding performance were attributed to the internal three-dimensional well-constructed RGO–AC–RGO interconnected conducting network. The prepared composites showed a stable shielding efficiency even after 1000 bend release cycles.

##### 5.3.4.8 Graphene nanoplatelets (GNP)

Graphene nanoplatelets (GNP) are unique nanoparticles comprising small stacks of 10–30 graphene sheets having a platelet shape, which are identical to those found in carbon nanotubes, but in a planar form. The unique size and platelet morphology of GNP make these nanoparticles especially effective for providing barrier properties, while their pure graphitic composition imparts them with excellent thermal and electrical characteristics. When they are added at 2–5 wt% to the polymer matrices, they form polymer composites that are not only thermally or electrically conductive and less permeable to gases, but simultaneously improve the physical–mechanical properties like the tensile strength, stiffness, surface toughness or design flexibility. The outstanding increase in the thermal conductivity of the GNP-based polymer composites can be attributed to the GNP 2D planar structure, which minimizes the phonon scattering on the polymer/nanofiller interface. The edges of the nanoparticles can be occupied with functional groups, such as carboxyls, ethers or hydroxyls. Those functional sites show the possibility of the surface modification and functionalization of GNP with other suitable materials, providing hydrogen or covalent bonding capability, as well as enable higher interactions with polymer matrices. Graphene nanoplatelets can be produced from natural graphite *via* liquid phase chemical exfoliation without further centrifugation steps. Other methods include shear-exfoliation, microwave radiation of acid-intercalated graphite, ball-milling or wet-jet milling. These synthesis techniques enable the production of a large variety of nanofillers in terms of thickness, aspect ratio or defect concentrations.^245^ Due to the easy processability, graphene nanoplatelets are economically viable and more preferred when compared to carbon nanotubes. In addition, they are more easily dispersed in polymer matrices in comparison with CNT. The multifunctional property improvements offered by GNP make them ideal additives in a variety of applications, such as sensors, actuators, aeronautics, electronics, and photovoltaics, as well as polymer composites for EMI shielding.^246–250^

Li *et al.*^251^ used a solvent-free latex technology to fabricate polyacrylate (PA)/graphene nanoplatelet composites with favorable build-up segregated GNP architecture stacked in the PA matrix. Owing to the unique nanostructured architecture of GNP with high aspect ratio, the room temperature percolation threshold for electrical conductivity was reached at remarkably low GNP content (0.11 wt%). The unexpectedly high complex permittivity evoked by induction of a strong Maxwell–Wagner–Sillars polarization at the PA/GNP interface was observed. The EMI SE of the tested composites showed an increasing trend with increasing GNP content, reaching the total shielding effectiveness of 66 dB over the X-band frequency range for the composite containing 6 wt% of GNP. It was found that the EMI SE resulted from the pronounced dielectric relaxation, conduction loss and multi-scattering. Bregman *et al.*^252^ developed non-perfect electrical conductor backed, porous composites based on poly-lactic acid/graphene nanoplatelets, and examined the effect of the pore geometry and GNP aspect ratio on the EMI shielding performance. The application of periodically arranged cylindrical pores resulted in a reduced fraction of the reflected power and enhanced fraction of the absorbed power within the majority of the X-band frequency range. Finally, the authors stated that a new strategy for the development of modern microwave absorption polymer composites is provided by tuning the shielding characteristics *via* the preparation of composite materials with periodically arranged pores. 3D lightweight, porous graphene-based aerogels for electromagnetic wave absorption were constituted by incorporation of GNP into the polyvinylidene fluoride matrix.^253^ It was shown that the electrical, thermal and mechanical properties of the aerogels can be adjusted by the selection of proper controlled processing temperature, at 65 °C or 85 °C. The tested aerogels are characterized by outstanding EMI characteristics, allowing the fabrication of absorbers with 9.2 GHz and 6.4 GHz and reflection coefficients under −10 dB and −20 dB in the frequency range of 8–18 GHz. In addition, the aerogels exhibited exceptional thermal conductivity without any considerable change in volume after temperature variations. Finally, the controlling of the process production parameters allows for obtaining water-repellent GNP-based aerogel polymer composites, which prevent their thermal and electromagnetic characteristics from being affected by humidity, and allowing the development of electromagnetic absorbers with a stable response. Liang *et al.*^254^ developed a template method for the preparation of 3D porous graphene nanoplatelets/reduced graphene oxide foam/epoxy (GNP/RGO/EP) nanocomposites, in which 3D RGO foam embedded with GNP builds a 3D thermal and electrical conductive network in the epoxy polymer matrix. The 3D RGO framework prevents the agglomeration of GNP, and behaves as an efficient cluster of tunnels for electrical transport and attenuates the EM waves. The GNP/RGO/EP composite containing 0.1 wt% of RGO and 20.4 wt% of GNP exhibited an EMI SE of 51 dB over the X-band frequency range. This means almost 292% enhancement in comparison with RGO/EP composites (∼13 dB) and 240% improvement when compared to GNP/EP composites with an absence of 3D microstructures (∼15 dB). The tested hybrid nanocomposites also showed an impressive electrical conductivity of 179.2 S m^−1^ and thermal conductivity of 1.56 W m^−1^ K^−1^. The study introduced a new strategy for the architecture of multi-functional EP nanocomposites for efficient heat dissipation and EMI shielding. Dul *et al.*^255^ reported the preparation and characterization of hybrid nanocomposites based on acrylonitrile–butadiene–styrene terpolymer with incorporated graphene nanoplatelets and carbon nanotubes, convenient for fused filament fabrication (FFF). The increase in the modulus and decrease in the tensile strength of nanocomposites were recorded with the increase in GNP content, while the presence of CNT led to the increase of electrical conductivity. The EMI SE of the compression-molded ABS-based nanocomposites filled only with CNT and dual hybrid GNP/CNT nanofillers reached higher than −20 dB in the X-band frequency range, while the FFF parts exhibited slightly lower EMI SE (in the range of −12 to 16 dB), depending on the building conditions.

##### 5.3.4.9 Graphene nanoribbons (GNR)

**Fig. 20:**
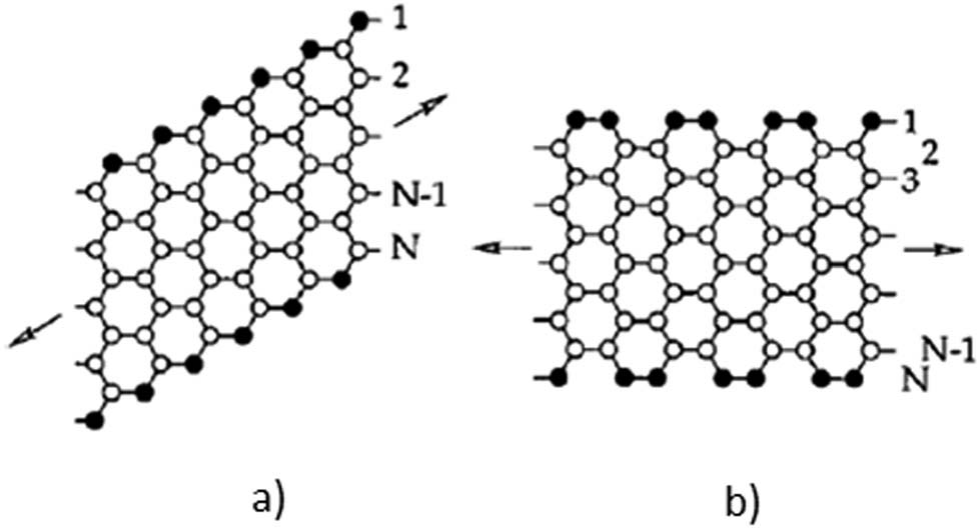
Structure of graphene nanoribbons, zigzag (a), armchair (b).

Graphene nanoribbons (GNR) are thin elongated graphene strips of sp^2^-bonded carbon atoms with a narrow width of 12–20 nm. Ideal ribbons exhibit simple edge orientation and termination. Due to a high symmetry, the most studied GNR are those with armchair and zigzag edges. Zigzag nanoribbons possess a honeycomb network oriented in such a way that the edge is made of the triangular edges of the hexagons (Fig. 20a). Armchair ribbons are oriented at 30° (or equivalently at 90°) from the zigzag orientation. In this case, the edge is formed from the hexagonal sides (Fig. 20b).^256^ GNR having frequent edge sides, ultra-high aspect ratio and more effective surface area than CNT, exhibit exceptional charge-transfer and electronic properties. In line with good mechanical, chemical, thermal and magnetic properties, GNR have become very promising nanofillers for a wealth of applications, such as energy storage, transistors, polymer nanocomposites, high strength materials, and EMI shielding.^257^ Graphene nanoribbons can be synthesized by bottom-up (epitaxial growth, molecular precursor-based growth, CVD method) and top-down (lithographic pattering methods, graphene cutting with catalytic particles) approaches.^256^ The third method of GNR fabrication is based on a longitudinal opening, or unzipping of multi-walled carbon nanotubes. Graphene nanoribbons synthesized *via* the longitudinal unzipping of MWCNT have been reported to be the best-suited nanofillers for polymer composite materials owing to their large surface area.^258^ In addition, under stringent controlled unzipping conditions, GNR can contain only a few ribbon layers, which facilitates the ease in their exfoliation in the polymer matrix when compared to GNP.

Joshi and co-workers^259^ developed a lightweight polyvinyl alcohol/graphene nanoribbons composite film, which exhibited excellent absorption-dominated EMI SE of 60 dB over the X-band frequency range at very low GNR content. By application of 0.0075 wt% of GNR in PVA matrix, the electrical conductivity increased by 14 orders due to the formation of an interconnected GNR network at a very low percolation threshold. Arjmand *et al.*^260^ made a comparative study of melt-compounded polyvinylidene fluoride nanocomposites filled either with graphene nanoribbons or multi-walled carbon nanotubes at filler loadings ranging from 0.3 to 2 wt%. The results revealed that despite the lower intrinsic filler conductivity and roughly comparable dispersion state, PVDF/MWCNT nanocomposites reached a lower percolation threshold than PVDF/GNR nanocomposites. This was attributed to the improved interlocking ability and thus stronger conducting network of MWCNT. At low filler loadings with no formed conductive filler network within the polymer matrix, PNDF/GNR nanocomposites exhibited higher EMI SE owing to the higher intrinsic filler conductivity and larger interacting surface area of GNR. On the other hand, MWCNT-based composites manifested higher electrical conductivity and EMI shielding performance at higher nanofiller contents, where the conductive filler network was formed. For example, PVDF/MWCNT nanocomposites containing 2 wt% of the nanofiller exhibited conductivity of 0.77 S m^−1^ and EMI SE of 11.60 dB in the X-band frequency range, which was eight orders of magnitude and twofold higher in comparison with equivalent PVDF/GNR counterparts. Having similar molecular structure, but various geometries of GNR and MWCNT, the higher EMI shielding characteristics of PVDF/MWCNT nanocomposites were attributed to the higher network formation ability and interlocking ability of the tubular nanostructured MWCNT than the ribbon-like nanostructured GNR.

#### 5.3.5 Nanostructured polymer composites filled with hybrid inorganic and carbon based fillers

The combination of carbon based fillers with metallic particles, metal oxides, ferrites and other ferroelectrics offers a great opportunity to adjust not only excellent physical–mechanical properties of polymer composites originating mainly from the presence of carbon based fillers, but also provides the possibility to tune permeability, permittivity, thermal and electrical conductivity or thickness to obtain improved EMI performance. Efficient electromagnetic impedance matching can be achieved by reaching a good balance between the relative complex permittivity *ε*_r_ and relative complex permeability *μ*_r_. This means that the suitable dielectric and magnetic loss tangent tan *δ*_E_ or *δ*_M_, respectively, are required to obtain the desired EM waves attenuation. The variations of the inorganic metal-based fillers with carbon fillers can provide a promising attitude to couple the magnetic characteristics of the magnetic materials and dielectric properties of the carbon inclusions to adjust the EM wave attenuation. A suitable combination of these different materials can result in better impedance matching at composite interfaces with reduced EMI reflection. In hybrid composites, both tan *δ*_E_ and tan *δ*_M_ contribute to the energy consumption, which shift towards improved shielding effectiveness *via* absorption mechanisms.^261,262^ Thus, this strategy has become very popular for the design of efficient EMI absorbers. Along with interfacial polarization and dipole polarization, carbon fillers with a large specific surface area and high aspect ratio contribute to the tan *δ*_E_ by formation of the network to scatter the charges. The tan *δ*_M_ is attributed to the eddy current loss, natural resonance and exchange resonance (occurring usually at higher frequencies) of the magnetic materials. Moreover, both magnetic and carbon based fillers can regulate *ε*_r_ and *μ*_r_, which is favorable for the impedance match. The balance can be achieved by controlling the weight ratio between the magnetic and dielectric materials.^55^

##### 5.3.5.1 Hybrid composites based on carbon-based fillers and metallic fillers

The segregated poly(vinylidene fluoride)/carbon black composites with an inclusion of magnetic carbonyl iron particles PVDF/CB/CI were first fabricated by simply compounding and hot compaction.^263^ The results showed that the application of carbonyl iron caused a remarkable increase in the magnetic characteristics and EMI shielding performance. The composite containing 3 wt% of CB and 20 wt% of CI with a thickness of 2 mm exhibited a saturation magnetization of 35.2 emu g^−1^ and overall shielding efficiency of 20–27 dB within the broadband 8–18 GHz frequency range. The thermal, thermo-mechanical, magnetic, electromagnetic and X-band frequency microwave absorption properties of graphite/cobalt filled polystyrene composites PS/GR/Co, synthesized by melt compounding and injection molding approach, were studied by Ansari *et al.*^264^ The minimum reflection loss (or maximum absorption loss) for the PS/GR/Co composites with a thickness of 1.8 mm and excess loading of GR flakes was recorded to be −32.02 dB (99.94% attenuation) at 10.13 GHz. The lightweight multicomponent PS/GR/Co composites possessing high absorption loss and low thickness are reported to be promising candidates for EMI shielding and stealth applications. AlZoubi *et al.*^265^ introduced a facile and cost-effective hydrothermal process to fabricate polyvinyl alcohol/graphite sheets/nickel oxide (PVA/GR/NiO) nanocomposites with high purity. The composite containing 15 wt% of NiO presented the best morphology and EMI SE of 32 dB in the 0–6 GHz frequency range. The authors stated that the improved EMI SE could be attributed to the percolation network formed through the introduction of conductive GR/NiO hybrid fillers in the polymer matrix, and the formation of higher amount of magnetic and electrical dipoles. In the scientific research,^266^ a simple chemical method with the introduction of hydrazine hydrate as a reductant was used to uniformly anchor silver nanoparticles on the surface of poly(acrylonitrile) (PAN) microspheres. The PAN/Ag hollow microspheres were combined with reduced graphene oxide and dispersed in epoxy resin to generate lightweight absorbers with improved microwave absorption performance. The minimum reflection loss was achieved at 8.8 GHz (−28.1 dB) with a composite thickness of 2 mm, and the effective absorption bandwidth (reflection loss less than −10 dB) ranged from 7.9 GHz to 9.8 GHz. However, the content of PAN/Ag and RGO was only 3 wt% and 1 wt%, respectively. As the amount of the PAN/Ag powder decreased to 1 wt%, the EP/PAN/Ag/RGO composite still retained favorable microwave absorption performance with the optimal reflection loss of −14.7 dB. Wan *et al.*^267^ proposed a creative multi-dimensional, level-by-level assembly approach to design sandwich-like nano-hetero-structures comprising flexible cotton-derived carbon fibers (CCF), dandelion-like graphene (DGN), and conductive and magnetic nickel nanoparticles (Ni) through a high-precision magnetron sputtering plasma-improved chemical vapor deposition. The multiple spatial-scale CCF/DLG/Ni composites exhibited outstanding conductivity of 625 S m^−1^ and remarkable EMI SE of 50.6 dB in the X-band frequency range. Moreover, the ultralight and ultrathin composites can be easily bent, folded and twisted, showing their simple and excellent processability for commercial applications. The highly conductive 3D graphene network was fabricated by vapor chemical deposition on a 3D nickel fiber network, followed by etching process.^268^ Subsequently, the vacuum infiltration method was introduced to prepare the flexible, lightweight, polydimethylsiloxane PDMS/GN composite by using the graphene network as a template. The composite with a GN content of only 1.2 wt% and thickness of 0.25 and 0.75 mm exhibited remarkable EMI SE of 40 and 90 dB, respectively, within the X-band frequency range and electrical conductivity of 6100 S m^−1^. Moreover, the tensile strength of the composite was significantly improved by incorporation of only 1.2 wt% GN. Liang *et al.*^269^ introduced the sol–gel template method to prepare 3D reduced graphene oxide/silver platelets foam (RGO/AgPS) with numerous spherical hollow structures. The combination of the prepared foam with epoxy resin resulted in the fabrication of 3D EP/RGO/AgPS nanocomposites showing strongly regular segregated structures. Due to the interconnected hollow conducting networks of RGO/AgPS and the interfacial synergy between EP and RGO/AgPS, the 3D EP/RGO/AgPS composites containing 0.44 vol% of RGO and 0.94 vol% of AgPS revealed the maximum EMI SE of 58 dB in the X-band frequency range (99.99% shielding of EM waves). This represents 274% enhancement when compared to that of the 3D EP/RGO nanocomposites (21 dB). In their work,^270^ Choudhary *et al.* stated that the EMI shielding efficiency of the composites based on polyvinylidene fluoride and carbon nanostructures embedded with cobalt nanoparticles is strongly dependent on the concentration of graphitic carbon and the magnetic properties of the Co particles. Co nanoparticles encapsulated by graphitic carbon and incorporated in the PVDF matrix were prepared by the one-pot pyrolysis process at two different temperatures, *i.e.*, 1000 °C (Co-1000) and 800 °C (Co-800). It was shown that the pyrolysis temperature plays a significant role in determining the morphology, structure and magnetic characteristics of the polymer matrix, graphite layer and cobalt nanoparticles. The Co-1000 sample exhibited a high saturation magnetization and higher content of graphitic carbon when compared to that of the Co-800 sample. This resulted in a higher EMI SE of PVDF/C/Co-1000 nanocomposite. The higher impedance mismatch and dielectric loss of the PVDF/C/Co-1000 nanocomposite originates from its more inhomogeneous dielectric medium. Finally, it was reported that the highly magnetic particles and higher degree of graphitization shift towards more effective EMI shielding of the polymer nanocomposites. The same group of authors investigated the X-band frequency range EMI shielding behavior of the flexible PVDF composites containing tubular- and globular-shaped carbonaceous nanostructures decorated with mono-metallic (Ni), as well as bi-metallic (CoNi, FeNi, MnNi) alloy nanoparticles.^271^ Pyrolysis was carried out at 800 °C and 1000 °C to fabricate carbon-structured materials having two different morphologies. Carbon nanotubes were mostly observed in the samples synthesized at 800 °C, while carbon globules (CG) were formed in the samples prepared at 1000 °C. The PVDF/CNT composites showed better microwave-shielding behavior than the PVDF/CG composites, which was attributed to the improved absorption of the EM microwaves through the interfacial polarization loss and ohmic conduction. The 1D structure of CNT provided the required conduction pathways for the electrons, and generated the network to trap the EM microwaves within them through multiple scattering. It was reported that the microwave absorption performance of the composites resulted mainly from the graphitic walls of CNT, the metallic nature of the embedded nanoparticles, and the graphitic layer encapsulating the metallic nanoparticles. The authors further demonstrated the direct correlation of the EMI shielding performance of the tested nanocomposites with the morphology of the carbon-structured nanomaterials and the conductivity of the implemented metallic nanoparticles. The influence of the nanocomposite preparation methods on the electromagnetic parameters, electrical conductivity and EMI SE of the polypropylene/nickel-coated carbon fiber PP/Ni-CF composites was investigated in the work performed by Lee *et al.*^272^ The composites were prepared by using an internal mixer, injection molding machine, and screw extruder. The highest electrical conductivity (17.5 S cm^−1^) and EMI shielding ability (48.4 dB at 10 GHz) exhibited composites prepared by injection molding. This can be attributed to the increased CF length when the PP/Ni-CF nanocomposite was fabricated by injection molding, which led to the formation of a conducting network within the composite. It was also reported that the major electromagnetic absorption mechanism was dielectric loss, namely the dipole and interface polarization and polarization between the filler and the polymer matrix, which resulted in the enhanced EMI absorption values.

##### 5.3.5.2 Hybrid composites based on carbon-based fillers and magnetic fillers

**Fig. 21:**
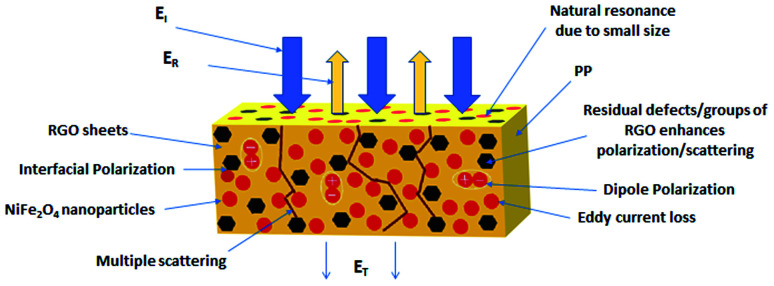
Schematic illustration of the EMI shielding mechanisms for the PP/RGO/NiFe_2_O_4_ composites (modified from ref. 275 with permission from the American Chemical Society, copyright 2019).

**Fig. 22:**
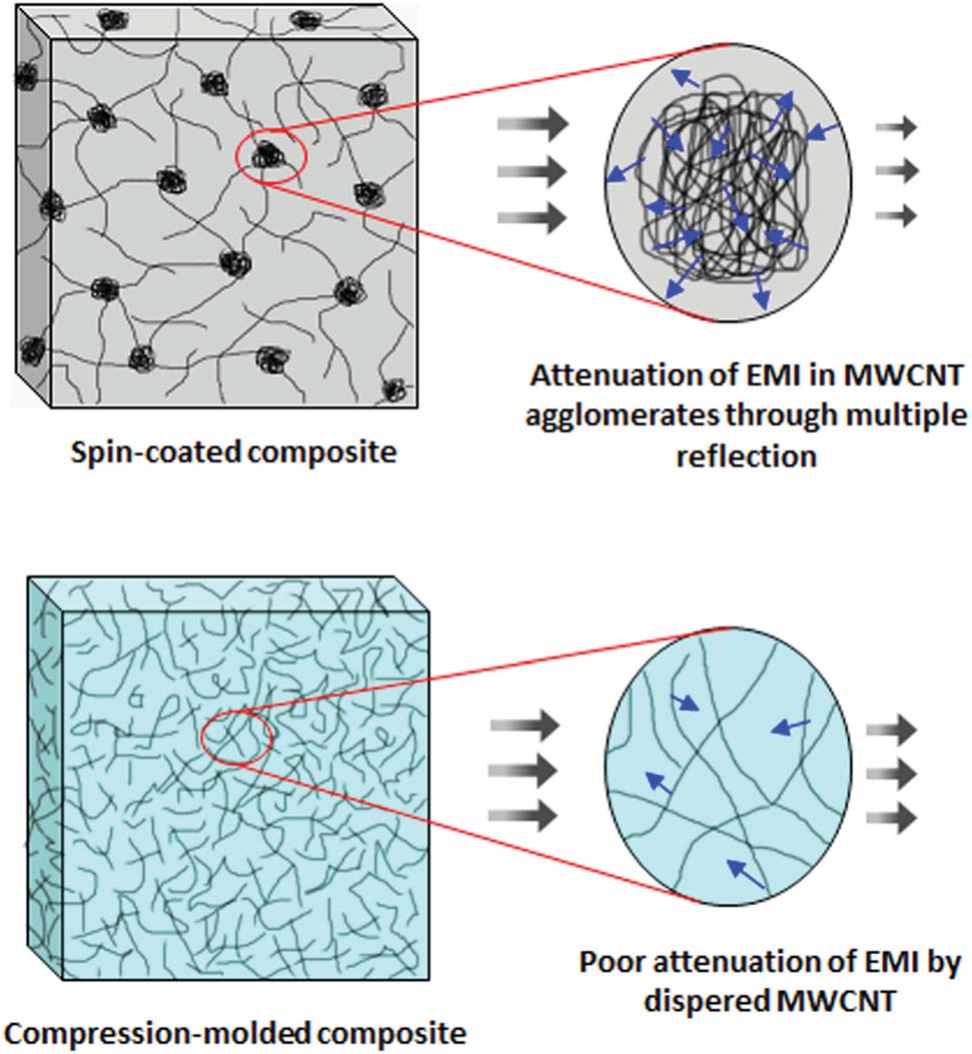
Schematic illustration of the EMI shielding mechanisms for the spin-coated and compression molded PDMS/MWCNT composites (modified from ref. 282 with permission from the American Chemical Society, copyright 2019).

Novel composites consisting of polyvinylidene fluoride, conductive carbon black and ferromagnetic Sr_3_YCo_4_O_10+*δ*_ (SYCO), prepared by the solution blending and coagulation method, were deeply studied for the first time in the work.^273^ It was revealed that during the nucleation process of PVDF, CB and SYCO facilitated the crystallization of semipolar γ and polar β phases, as well as the nonpolar α phase in PVDF matrix. The electrical dc conductivity of PVDF significantly increased from 1.54 × 10^−8^ to 9.97 S m^−1^ with application of 30 wt% CB, and it was almost constant with respect to the content of SYCO. The thorough analysis of the shielding efficiency within the 8.2–18 GHz frequency range showed that in addition to the magnetic and conductive losses due to SYCO and CB, respectively, the synergy among PVDF, CB and SYCO facilitates shielding by input impedance matching, improving the multiple internal reflection from SYCO followed by subsequent CB absorption, dielectric damping losses, eddy current losses and interfacial polarization losses. The proposed shielding mechanisms contributed to the excellent EMI SE of 50.2 dB, from which 41.2 dB was the contribution of absorption. Bhattacharyya *et al.*^274^ reported the research on the *in situ* synthesis of graphene oxide GO/Ni_0.5_Zn_0.5_Fe_2_O_4_ ferrite nanoparticles framework by gel-combustion method, followed by the preparation of ultrathin (100–120 μm), structurally stable, hybrid polyurethane coating on the aluminum substrate, and its application on EMI shielding within the microwave frequency region. The microstructure study revealed that small sized ferrite particles (17 nm) were homogeneously grafted onto the GO layers. The nanocomposite coating showed excellent broadband absorption characteristics with an absorption ability higher than 90% within the 6 GHz bandwidth. As the GO content in the composites increased, the absorption frequency bandwidth range increased with improved absorptivity. Moreover, the hybrid nanocomposite coating exhibited ultrahigh absorptivity over 8–12 GHz frequency band. In the research work,^275^ a dextrin-mediated sol–gel combustion processing method was introduced to synthesize the NiFe_2_O_4_ nanoparticles, which were subsequently annealed at 600, 800 and 1000 °C. The temperature of annealing played a significant role in the tuning of the physical characteristics and dimensions of the NiFe_2_O_4_ nanoparticles (the average crystallite size was observed to be 20.6, 34.5 and 68.6 nm for nanoparticles annealed at 600, 800 and 1000 °C, respectively). The prepared NiFe_2_O_4_ nanoparticles were dispersed in polypropylene matrix engineered with reduced graphene oxide. The overall EMI SE for the prepared nanocomposites with 2 mm thickness was recorded to be 45.56, 36.43, and 35.71 dB over the X-band frequency regime for nanoparticles annealed at 600, 800 and 1000 °C, respectively. The specific EMI SE was 38.81, 32.79, and 31.73 dB cm^−3^ g^−1^, while the absolute EMI SE was 388.1, 327.9, and 317.3 dB cm^−2^ g^−1^ for nanoparticles annealed at 600, 800 and 1000 °C, respectively. It was also revealed that the main contribution to the shielding efficiency originated from dielectric loss, magnetic loss, synergetic effect, good impedance matching and high attenuation constant (Fig. 21). The same collective of authors also performed a similar research study, the valuable results of which were published in the work.^276^ Ahmed *et al.*^277^ fabricated hybrid PVDF/GNP/NSF composites by dispersion of few layered graphene nanoplatelets and nickel spinel ferrite (NSF) into a polyvinylidene fluoride matrix. The content of GNP was kept on constant level 3 wt%, while the amount of NSF varied from 15–30 wt%. The EMI SE measured in the frequency range of 1–12 GHz increased up to the level of 25–45 dB for PVDF/GNP composites, when compared to ∼0 dB for pure PVDF. With the addition of NSF up to 15 wt%, the absorption-dominated EMI SE was improved to 30–53 dB, clearly indicating the effective network formation and interactions of GNP and NSF in the PVDF matrix. With increasing content of NSF up to 30 wt%, the EMI SE was reduced to 12–43 dB due to the agglomeration of nickel ferrite particles. In the scientific study performed by Zhan *et al.*,^278^ anisotropic polymer composites were synthesized by combining the layer-by-layer filtration (LBL) method with the alternative assembling of the hexagonal boron nitride (BN) flakes and carbon nanotubes on natural rubber (NR) latex particles. The layered composites showed anisotropic electrical and thermal conductivity, which were tailored *via* the layer formulations. The NR/CNT/BN composite consisting of four NR layers with 8 phr (7.4 wt%) of CNT (phr – parts per hundred rubber) and four alternate layers containing 12 phr (10.7 wt%) of BN exhibited the specific EMI SE of 22.41 dB mm^−1^ at 10.3 GHz and thermal conductivity coefficient of 0.25 W m^−1^ K^−1^. A multiscale approach was adapted to improve the EMI shielding performance of the carbon fiber veil (CFV) epoxy resin based composites.^279^ The Fe_3_O_4_ nanoparticles were surface-treated with silane coupling agent, and subsequently dispersed in the epoxy matrix. The EP/CFV/Fe_3_O_4_ multiscale composites were fabricated by impregnating the CF veils with an EP/Fe_3_O_4_ mixture to form prepreg with a following vacuum bagging process. The overall shielding efficiency showed an increasing trend with increasing loading of Fe_3_O_4_, and the maximum EMI SE of 51.5 dB within the X-frequency band was reached at a low composite thickness of 1 mm. The increase in the absorption loss was attributed to the improved dielectric, as well as magnetic loss generated by Fe_3_O_4_, and the multilayer construction of the composites. The ternary polymer/MWCNT/flaky Fe–Si–Al alloy (Sendust) composites were fabricated by introducing a twin-screw internal compounder and a roll-milling device.^280^ The application of MWCNT increased the dielectric loss of the composite by generation of conductive paths and improved dipolar, as well as interfacial polarization. With the application of 5 wt% MWCNT, the composite exhibited the reflection loss of −17 dB at 4.5 GHz. However, in the near field, the power loss increased more rapidly with increasing content of MWCNT. The absorption efficiency and overall EMI SE of the ternary polymer/MWCNT/Sendust composite increased more significantly when compared to the binary Sendust/polymer composite owing to the dielectric property improvement by MWCNT. In the study done by Wang *et al.*,^281^ durable and flexible EMI shielding cotton fabric was prepared by a layer-by-layer assembly of MWCNT and nickel ferrite nanoparticles (NiFe_2_O_4_), followed by subsequent poly(dimethylsiloxane) coating. Benefiting from the excellent interfacial compatibility and interactions between NiFe_2_O_4_ and MWCNT, the efficient magnetic, electrical and thermal conductive paths were successfully generated on the cotton fabric. The tested composite fabric with the thickness of 0.96 mm exhibited high electro-magnetic properties, ultrahigh EMI SE of 84.5 dB within the X-band frequency range, and significantly improved the thermal conductivity (2.52 W m^−1^ K^−1^). Moreover, the external PDMS coating not only endowed the composite with water-resistant capability, but also enhanced the structural stability, while preserving the desirable air permeability. The role of the two-polymer processing methods, namely compression molding and spin coating, was investigated for the preparation of hybrid elastomer composites based on poly(dimethylsiloxane) and MWCNT for EMI shielding applications in the Ku-band.^282^ In comparison with well-dispersed MWCNT in the composite films prepared by compression-molding, individual agglomerates of MWCNT in the spin-coated composites attenuated incident EM radiation more effectively through extensive interactions of electromagnetic waves with MWCNT due to dense packing in the formed agglomerates (Fig. 22). Thus, PDMS composites were compounded with MWCNT and Fe_3_O_4_ nanoparticles *via* the spin coating, and subsequently stacked with a gradient of filler concentration. The hybrid composite film having 0.9 mm in thickness exhibited a maximum EMI SE of −28 dB (99% of the incident EM radiation). Kar *et al.*^283^ reported tailor-made properties and EMI shielding behavior in the co-continuous polymer blend (PVDF/ABS) embedded with multi-walled carbon nanotubes and ferrite (Fe_3_O_4_) or barium titanate (BaTiO_3_). To enhance the dispersion state, the MWCNT particles were surface-modified with 3,4,9,10-perylenetetracarboxylic dianhydride through π–π stacking. For efficient EMI shielding, carbon nanotubes were conjugated with either Fe_3_O_4_ or BaTiO_3_ nanoparticles *via* suitable modifications. The hybrid nanoparticles were selectively localized predominantly in the PVDF polymer phase owing to its polarity, and showed outstanding microwave attenuation. The microwave parameters were deeply assessed in order to gain insight into the magnetic and dielectric features. The achieved results proposed the polymer blend to be a very promising candidate for designing thermally stable, lightweight microwave absorbing materials. The application of a segregated microstructure technique leads to the higher dispersion of magnetic fillers within the polymer matrices, and improvement of the electrical conductivity of the polymer composites. Sharif *et al.*^284^ reported the fabrication of composites based on poly(methyl methacrylate)/reduced graphene oxide with and without decorated magnetite particles having a segregated structure. For that purpose, an emulsifier-free emulsion polymerization was used. The PMMA/RGO nanocomposite with 2.6 vol% of RGO exhibited electrical conductivity of 91.2 S m^−1^ and EMI SE of 63.2 dB (with 2.9 mm of nanocomposite thickness). It was also revealed that decorating RGO with magnetite nanoparticles resulted in a significant increase of the shielding effectiveness. For example, the PMMA/RGO nanocomposite having 1.1 vol% of RGO showed EMI SE of 20.7 dB, while the application of 0.5 vol% magnetite nanoparticles improved EMI SE up to 29.3 dB. The outstanding electrical properties of nanocomposites were attributed to the superiorities of the segregated conductive structure on the one hand, and to magnetic characteristics of the magnetite nanoparticles on the other hand.

##### 5.3.5.3 Hybrid composites based on carbon-based fillers and MXenes

Despite the fact that MXenes are a relatively new class of materials, the advancement in the research studies of EMI shielding materials has made a great progress leading to the development of hybrid polymer composites based on MXenes and carbon-based fillers, which represent the high level of engineering polymer materials with the effects of EMI shielding.

Cao *et al.*^285^ fabricated a flexible and ultrathin cellulose nanofibrils/CNT/Ti_3_C_2_T_*x*_ MXene composite paper with a sandwich and gradient structure by introduction of a vacuum-assisted filtration approach. The prepared composite paper exhibited an outstanding combination of high toughness (2.1 MJ m^−3^), tensile strength (97.9 MPa), fracture strain (4.6%) and electrical conductivity (2506.6 S m^−1^). The gradient structure made a great difference to the contributions from absorption and reflection, resulting in the overall EMI SE of 38.4 dB in the X-band frequency range. The authors claimed that the new type of polymer composite paper with excellent EMI shielding performance will significantly broaden the practical applications in the fields of portable and wearable electronic devices. Weng *et al.*^286^ introduced a spin spray layer-by-layer advanced nanocomposite preparation approach to fabricate poly(vinyl alcohol) and poly(sodium 4-styrene sulfonate) PVA/PSS/CNT/Ti_3_C_2_T_*x*_ MXene composite films having excellent conductivity up to 130 S cm^−1^ and high specific EMI SE of 58 187 dB cm^−2^ g^−1^ over the X-band frequency range. Those outstanding features can be attributed to the high electrical conductivity of MXene and CNT, and improved absorption of the layer-by-layer architecture of the films. The authors stated that the spin spray layer-by-layer technique allows for unique combinations of polymers and nanostructured materials, offering a notable platform for development of hierarchical structures with desirable multi-functionalities including excellent conductivity, controllable thickness and transparency, as well as flexibility and high stability. PVDF/GN/Ti_3_C_2_T_*x*_ MXene flexible, thin, low-density composites were synthetized by a spray coating and an effective solvent casting approach.^287^ The composites exhibited outstanding conductivity of 13.68 S cm^−1^ with 3.1 Ω sq^−1^ sheet resistance, excellent thermal stability and restricted the weight changes until 400 °C. The excellent EMI shielding efficiency of 53.8 dB (99.99%) with absorption of 43.38 dB and reflection of 13.10 dB in the X-band frequency range was recorded for the tested composites. The single coated carbon fabric composite showed an absolute shielding efficiency of remarkable 35 370 dB cm^−2^ g^−1^, which points to the utilization in perspective applications such as radars, aeronautics, air travels, military applications, mobile phones or handy electronics. In the study,^288^ structural RGO was constructed by using an Al_2_O_3_ honeycomb template, and Ti_3_C_2_T_*x*_ MXene was subsequently self-assembled on RGO through electrostatic adsorption. The structural honeycomb RGO/Ti_3_C_2_T_*x*_ MXene was then compounded with epoxy resin to obtain 3D EP/RGO/Ti_3_C_2_T_*x*_ MXene composites with a synergistic effect of Ti_3_C_2_T_*x*_ MXene, and RGO resulting in excellent electrical conductivity and EMI shielding effectiveness. The composite film with the thickness of 0.5 mm and composition 3.3 wt% of Ti_3_C_2_T_*x*_ MXene + 1.2 wt% of RGO showed outstanding electrical conductivity of 387.1 S m^−1^ and EMI SE of 55 dB in the X-band frequency range. Wang *et al.*^289^ fabricated porous 3D Ti_3_C_2_T_*x*_ MXene/C hybrid foam/epoxy nanocomposites by thermal reduction and vacuum-assisted impregnation with subsequent curing treatment. The composite exhibited the electrical conductivity of 184 S m^−1^ and EMI SE of 46 dB within the X-frequency band. Moreover, Young's modulus reached the value of 3.96 GPa, while the hardness of the composite was 0.31 GPa. The formed conductive networks provide space for dissipation and attenuation of EM waves by multi-reflection and re-absorption with dominant absorption shielding mechanism.

#### 5.3.6 Multicomponent polymer composite systems

Since the development and fabrication of polymer composite structures for EMI shielding applications, a lot of approaches have been implemented in ordered to fulfill different requirements, depending on the application field. As the development in the current field of interest has progressed in an enormous speed, novel and modern attitudes have appeared with respect to new advancements. Multicomponent structures present hybrid materials that combine different constituents contributing to the electromagnetic shielding efficiency into one structural unit, presenting a top-level of EMI shielding multitasking polymer composites.

Novel EMI absorbers based on the epoxy matrix containing graphite nanosheets GRN/Fe_3_O_4_ composites decorated comb-like MnO_2_ were successfully fabricated *via* a two-step approach, and examined for EMI shielding effectiveness in the study.^290^ The ternary EP/GRN/Fe_3_O_4_/MnO_2_ composites exhibited improved microwave absorption performance owing to the complementary effects of the magnetic material (Fe_3_O_4_), dielectric component (MnO_2_), and electrically conductive material (GRN). The maximum reflection loss of the tested composites was observed to be −31.7 dB at 5.85 GHz and absorbing thickness of 4.5 mm. The efficient frequency range below −10 dB reached up to 4.47 GHz (11.87–16.34 GHz) with 2 mm matching thickness. Zhang *et al.*^291^ fabricated multilayered structured composites consisting of carbon fibers, multi-walled carbon nanotubes, carbonyl iron powder and epoxy resin. The bottom side of the composites was bonded with copper foil, which served as a reflective layer. The unique architecture of the multilayered structural design and outstanding interlayer interface integration resulted in multiple shielding peaks over the 1–18 GHz frequency range due to the multiple loss mechanisms. The maximum EMI SE of 73.8 dB at 2.3 GHz was recorded for the tested composites. In addition, the composites exhibited more balanced shielding efficiency within the tested frequency range when compared to the composites having only one type of filler. Hierarchical composites based on epoxy resin/MWCNT/Fe_3_O_4_/Ag were obtained from the acyl–amine reaction between carboxylation of Fe_3_O_4_/Ag–COOH nanoparticles and amino-functionalized MWCNT–NH_2_, followed by blending with the epoxy matrix.^292^ The prepared composites exhibited high electrical conductivity of 0.280 S cm^−1^, excellent thermal stability, outstanding Young's modulus of 4.60 GPa and hardness value of 0.26 GPa, and satisfactory EMI SE of 35 dB in the X-band frequency range. The introduction of Fe_3_O_4_/Ag nanoparticles not only facilitated the formation of conductive networks, leading to higher thermal and electrical conductivity and higher EMI SE, but also contributed to the hysteresis loss of the EM waves. It also offered more interfaces to re-absorb and reflect the EM radiation, resulting in significantly enhanced attenuation of EM waves. Pawar *et al.*^293^ fabricated flexible, lightweight and easy-to-process microwave absorbers by dispersing conductive multi-walled carbon nanotubes and two-dimensional reduced graphene oxide sheets decorated with silver nanoparticles (RGO/Ag) in poly(ε-caprolactone). The application of MWCNT resulted in the formation of the conductive pathways for charge transfer within the PCL matrix, while the attenuation was adjusted by multiple scattering owing to the high specific surface area of RGO/Ag. It was found out that this strategy resulted in a markedly improved microwave attenuation of PCL/MWCNT/RGO/Ag nanocomposites, in contrast to the incorporation of individual particles. The maximum EMI SE was −37 dB with an impressive absorption capability of 91.3%. Sun *et al.*^294^ constructed the carbonized melamine foam CMF/PDMS/GN/Fe_3_O_4_/Au nanoparticles multifunctional 3D hierarchical architecture, which demonstrated high conductivity (81.3 S m^−1^), excellent superparamagnetism (*M*_s_ = 22.6 emu g^−1^), low density (116 mg cm^−3^), high specific surface area (708 m^2^ g^−1^) and good compressive strength (110 KPa), altogether leading to the substantial EMI shielding performance. The EMI SE of the multicomponent composite film was reported to reach 30.5 dB within the X-band frequency range, which is equal to a specific EMI SE of 263 dB cm^−3^ g^−1^. The work done by Nguyen^295^ reports the fabrication of a unique 3D porous GN network decorated with the Fe_3_O_4_/Ti_3_C_2_T_*x*_ MXene hybrids in the PDMS matrix with excellent EMI shielding characteristics and pressure sensing performance. The composite with a thickness of only 1 mm exhibited a high porosity of 47%, outstanding conductivity of 630 S m^−1^ and remarkable EMI SE of 80 dB over the X-band and 77 dB over the Ka-band frequency range. The application of Fe_3_O_4_/Ti_3_C_2_T_*x*_ MXene hybrids in the PDMS/GN composite, resulting in the improvement of absorption shielding efficiency owing to the abundance of conductive layers, interfacial polarization and magnetic loss. Finally, the authors stated that flexible, lightweight, highly conductive multicomponent composite systems can open the new possibility for applications of the complex EMI shielding skin in robotics and wearable electronics.

## 6 Conclusion and future outlooks

The current study provides detailed insight into conductive polymers and polymer composites filled with different inorganic metallic and magnetic fillers, as well as organic carbon-based fillers and their combinations, which are able to shield harmful electromagnetic radiation. After a brief introduction of the theoretical fundamentals, the relationships between the morphology, structure, functionalization of the materials with respect to their magnetic, dielectric, conductive and EMI shielding characteristics are closely described. Polymers and polymer composites are versatile and widely used materials. In contrast to heavily-massed ceramics, concretes and metals, they exhibit the ease of processability, low density, good corrosion resistance or high design flexibility, tunable mechanical and structural properties. In addition, depending on the composition, type and amount of the filler, they exhibit small matching thickness, high absorption, and wide frequency bandwidth, pointing to their potential for various EMI shielding applications.

The development in the field has progressed in enormous speed over the last several years, and many challenges have been still demanding. The shielding by absorption has become the most desirable, since the absorbed electromagnetic wave is efficiently attenuated and not emitted back to the surrounding. Although many fabricated materials exhibit high absorption ability, their absorption shielding is limited to the narrow frequency bandwidth or high shield thickness. Thus, the development of multifunctional polymer composite systems seems to be the future trend to reach the requirements, where the suitable correlation among permittivity, permeability, conductivity, frequency and thickness can be achieved. Another factor that must be taken into account is the weight, physical–mechanical and functional properties of the final materials. The main mechanism of shielding of metallic fillers is mostly based on reflection, which is not desirable. However, the utilization of metallic inclusions as the back side of the shield can provide the solution for EMI to be re-reflected to the body of the shield, in which it can be efficiently absorbed. Magnetic fillers possessing electric and/or magnetic dipoles exhibit good absorption ability. The disadvantage of inorganic fillers is that their incorporation into the polymer matrices is usually connected with the deterioration of the physical–mechanical properties and thermo-oxidative stability of the composites. High-density metals and ferrites also increase the weight of the composites. On the other hand, carbon-based fillers show high permittivity, excellent conductivity, low density, as well as outstanding physical properties. Thus, they contribute not only to the good shielding efficiency, but also to the improved physical–mechanical properties of the corresponding composites. Moreover, due to the high aspect ratio, the percolation threshold can be reached at lower filler content. It is believed that the suitable combination of the fillers in a proper content and ratio, together with the proper selection of polymer matrix/matrices might be the key for the development of high-tech materials, demonstrating not only excellent shielding performance, but also applicable physical–mechanical and structural features. The recently introduced novel ultra-efficient EMI shielding composite foams, hydrogels, aerogels or advanced multicomponent or multilayer 3D composite architectures present the cutting-edge materials with high application potential in different areas of practical, scientific, military and commercial applications.

## Conflicts of interest

The authors declare no conflict of interest.

## Supplementary Material
